# The human health effects of unconventional oil and gas development (UOGD): A scoping review of epidemiologic studies

**DOI:** 10.17269/s41997-024-00860-2

**Published:** 2024-03-08

**Authors:** Amira M. Aker, Michael Friesen, Lisa A. Ronald, Mary M. Doyle-Waters, Tim K. Takaro, Willow Thickson, Karen Levin, Ulrike Meyer, Elyse Caron-Beaudoin, Margaret J. McGregor

**Affiliations:** 1 Université Laval, CHU de Quebec – Université Laval, Québec, QC Canada; 2 Faculty of Health Sciences, Simon Fraser University, Burnaby, BC Canada; 3 Centre for Clinical Epidemiology and Evaluation, Vancouver Coastal Health Research Institute, Vancouver, BC Canada; 4 Department of Family Practice, Faculty of Medicine, University of British Columbia, Vancouver, Canada; 5 Emerald Environmental Consulting, Kent, OH USA; 6 Department of Health and Society and Department of Physical and Environmental Sciences, University of Toronto Scarborough, Toronto, ON Canada; 7 Dalla Lana School of Public Health, University of Toronto, Toronto, ON Canada

**Keywords:** Hydraulic fracturing, Fracking, Unconventional oil and gas development, Indigenous, Environmental justice, Fracturation hydraulique, fracturation, exploitation pétrolière et gazière non conventionnelle, Autochtones, justice environnementale

## Abstract

**Objective:**

Unconventional oil and gas development (UOGD, sometimes termed “fracking” or “hydraulic fracturing”) is an industrial process to extract methane gas and/or oil deposits. Many chemicals used in UOGD have known adverse human health effects. Canada is a major producer of UOGD-derived gas with wells frequently located in and around rural and Indigenous communities. Our objective was to conduct a scoping review to identify the extent of research evidence assessing UOGD exposure–related health impacts, with an additional focus on Canadian studies.

**Methods:**

We included English- or French-language peer-reviewed epidemiologic studies (January 2000–December 2022) which measured exposure to UOGD chemicals directly or by proxy, and where health outcomes were plausibly caused by UOGD-related chemical exposure. Results synthesis was descriptive with results ordered by outcome and hierarchy of methodological approach.

**Synthesis:**

We identified 52 studies from nine jurisdictions. Only two were set in Canada. A majority (*n* = 27) used retrospective cohort and case–control designs. Almost half (*n* = 24) focused on birth outcomes, with a majority (*n* = 22) reporting one or more significant adverse associations of UOGD exposure with: low birthweight; small for gestational age; preterm birth; and one or more birth defects. Other studies identified adverse impacts including asthma (*n* = 7), respiratory (*n* = 13), cardiovascular (*n* = 6), childhood acute lymphocytic leukemia (*n* = 2), and all-cause mortality (*n* = 4).

**Conclusion:**

There is a growing body of research, across different jurisdictions, reporting associations of UOGD with adverse health outcomes. Despite the rapid growth of UOGD, which is often located in remote, rural, and Indigenous communities, Canadian research on its effects on human health is remarkably sparse. There is a pressing need for additional evidence.

**Supplementary information:**

The online version contains supplementary material available at 10.17269/s41997-024-00860-2.

## Résumé

**Objectif:**

L’exploitation pétrolière et gazière non conventionnelle (EPGNC, parfois appelée « fracturation » ou « fracturation hydraulique ») est un processus industriel d’extraction du méthane et/ou de gisements de pétrole. De nombreux produits chimiques utilisés dans l’EPGNC ont des effets indésirables connus sur la santé humaine. Le Canada est un grand producteur de gaz dérivé de l’EPGNC, dont les puits sont souvent situés à l’intérieur et autour de communautés rurales et autochtones. Nous avons mené une étude de champ pour déterminer l’étendue des données de recherche évaluant les effets sur la santé de l’exposition à l’EPGNC, en nous concentrant plus particulièrement sur les études canadiennes.

**Méthode:**

Nous avons inclus des études épidémiologiques en anglais ou en français évaluées par les pairs (janvier 2000 à décembre 2022) qui mesuraient l’exposition directe ou indirecte aux produits chimiques de l’EPGNC et dans lesquelles les résultats cliniques étaient plausiblement causés par l’exposition aux produits chimiques liés à l’EPGNC. La synthèse des résultats est descriptive, et les résultats sont ordonnés selon les résultats cliniques et l’approche méthodologique.

**Synthèse:**

Nous avons identifié 52 études menées dans neuf juridictions. Deux seulement étaient canadiennes. La majorité (n = 27) faisaient appel à des cohortes rétrospectives ou étaient des études cas-témoins. Près de la moitié (n = 24) portaient sur les issues de la grossesse, et la majorité (n = 22) déclaraient une ou plusieurs associations indésirables significatives entre l’exposition à l’EPGNC et : l’insuffisance de poids à la naissance; la petite taille du bébé pour son âge gestationnel; la naissance avant terme; et une ou plusieurs anomalies congénitales. D’autres études faisaient état d’effets indésirables, dont l’asthme (n = 7), les troubles respiratoires (n = 13), les troubles cardiovasculaires (n = 6), la leucémie aiguë lymphoblastique infantile (n = 2) et la mortalité toutes causes confondues (n = 4).

**Conclusion:**

Il existe dans différents pays un corpus croissant d’études qui font état d’associations entre l’EPGNC et des résultats sanitaires indésirables. Malgré la croissance rapide de l’EPGNC, souvent présente dans des communautés éloignées, rurales et autochtones, la recherche canadienne sur ses effets sur la santé humaine est remarquablement clairsemée. Il y a un besoin urgent de recueillir d’autres données probantes à ce sujet.

## Introduction

Unconventional oil and gas development (UOGD, sometimes referred to as “fracking” or “hydraulic fracturing”) is an industrial process to extract methane gas and/or oil deposits primarily from shale or “tight” rock (Environmental Protection Agency, 2023). The technique first involves a pad preparation phase with clearing land and transporting material to the site. Next, a shaft is drilled vertically down 3–4 km into the ground—past the fresh and saline water aquifers, and horizontally for a further thousands of metres (“the spud” or drilling phase) (Rasmussen et al., 2016). This is followed by the hydraulic fracturing or “fracking” phase. In this phase, large amounts of fluid—most commonly water mixed with sand and chemical additives—are pumped along the well shaft under high pressure creating micro-fractures of the shale or tight rock, thereby freeing trapped oil and gas and starting the final production phase (US EPA, 2013; Water Resources Mission Area, 2019). During the production phase, the internal pressure of the rock formation causes fluid to return to the surface through the wellbore. This fluid is known as “flowback” or “produced water” and may contain the injected chemicals plus naturally occurring materials such as brines, metals, radionuclides, and hydrocarbons (Brown, 2014; Srebotnjak, 2018). The flowback is typically stored on site in tanks or open pits or surface impoundments before treatment, disposal, or recycling (Brown, 2014).

Public health concerns about UOGD include demonstrated carcinogenic, mutagenic, and endocrine-disrupting chemicals in fracking fluid (Colborn et al., 2011; Elliott et al., 2017; Horwitt, 2021; Kassotis et al., 2016; Xu et al., 2019). Environmental chemical release has been well documented from spills, and disruption of well and wastewater pond integrity (Bonetti et al., 2021; Wisen et al., 2020). Air pollution from diesel trucks, compressor and separation station engines, and methane release are additional concerns (Garcia-Gonzales et al., 2019). These pollutants, including volatile organic compounds (VOCs), nitrogen oxides, particulate matter, non-methane hydrocarbons, and hydrogen sulfide (Gilman et al., 2013; Macey et al., 2014; Moore et al., 2014), have known adverse human health impacts (Manisalidis et al., 2020). A further concern is the flowback of fracking fluids containing heavy metals, carcinogens, other toxicants (Crosby et al., 2018), and naturally occurring radioactive materials (NORMS) (Lauer et al., 2016).

Multiple jurisdictions have imposed UOGD bans or moratoria out of concerns for environmental and health impacts (AIDA, 2019). Within Canada, there has been substantial inter-provincial policy variation, with some provinces declaring moratoria or bans (Minkow, 2017), and others investing heavily in expansion (Schmunk, 2018). Canada is a major methane gas producer globally, with Alberta and British Columbia as the largest producers (Statista, 2014). Notably, most UOGD in Canada occurs in remote and rural communities (Natural Resources Canada, 2016) where Indigenous people are more likely to reside (Government of Canada, 2022). Indigenous communities living in rural and remote locations also rely on the land for food and traditional medicines, and land and water are embedded into peoples’ livelihood and identity (Poirier & Neufeld, 2023). Disparities in environmental exposures among Indigenous, Black, and other racialized communities have been documented in numerous settings (Hoover et al., 2012; Johnston et al., 2020; Kaufman & Hajat, 2021; Waldron, 2020), including in the context of UOGD, and there is growing recognition of environmental racism and environmental injustice as determinants of health (Waldron, 2020).

Systematic (Bamber et al., 2019) and scoping reviews (Deziel et al., 2020; Wright & Muma, 2018) have been published on the human health effects of UOGD over the past 5 years. However, as a relatively new area of research, more studies are being published annually. Our primary objective was to conduct a scoping review to update the available evidence on the health effects of UOGD-related chemical exposures and to identify knowledge gaps (Munn et al., 2018). Considering the rapid growth of UOGD in Canada (Atkinson et al., 2016), we additionally sought to identify Canadian studies. We limited our approach to a scoping review (Tricco et al., 2018) without meta-analysis or systematic assessment of study bias based on the substantial heterogeneity of exposures, outcomes, and methodological approaches.

## Methods

### Data sources and searches

We defined UOGD as directional vertical and horizontal drilling for long distances combined with the injection of fluids containing chemicals and proppants (for example, silica) with enough pressure to fracture shale formations thereby releasing oil or gas or both. We excluded coal seam gas studies because the extraction technique is often too different to make meaningful comparisons between exposures.

A biomedical librarian (MDW) conducted comprehensive searches in MEDLINE, and Embase (OVID) for all published studies in English or French from January 2000 through December 2022, with the most recent search completed on 10 January 2023. Our search concept included the various terms deployed for UOGD *AND* (population health *OR* pregnancy *OR* physical health *OR* Indigenous) and was combined with a search for UOGD-related toxicology studies (to be reported elsewhere). The search strategy is detailed in Online Resource 1. We also hand-searched the Physicians, Scientists, and Engineers for Healthy Energy citation database of oil and gas research health folder, to identify other eligible studies (PSE, 2023).

### Study selection

**Fig. 1:**
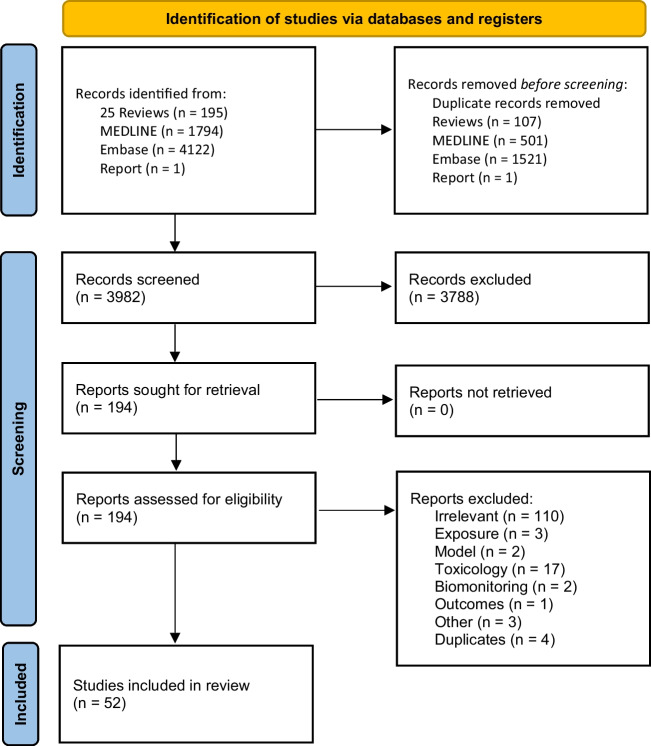
Summary of selection of studies included in scoping review

We included epidemiology studies which measured exposure to UOGD chemicals directly or by proxy. Using a similar approach to Bamber et al. (2019), we included studies where health outcomes were plausibly caused by UOGD-related chemical exposure. We excluded studies on UOGD and traffic accidents, occupational injury, anxiety, or depression where the hypothesized causal pathway for these outcomes was less likely related to chemical exposure and more likely related to indirect pathways such as income, industrial safety practices, and community disruption. We further excluded studies with no comparison group or reference population and studies that assessed the association of UOGD on climate change, seismicity, air/water/soil quality, animal health, community disruption, or socioeconomic impacts (Fig. 1).

Title and abstract screening and full text review were carried out by two pairs of reviewers (MMcG, MF, AA, TT) to determine eligibility for study inclusion. Disagreements at either of these stages were resolved through discussion with a fifth reviewer (LAR) until consensus was reached. Due to the wide heterogeneity of outcomes, exposures, and methodological approaches, we did not apply a formal bias tool to evaluate the quality of studies.

### Data extraction

Data for selected articles were independently extracted in duplicate by two pairs of reviewers (MMcG, MF, AA, TT) into an electronic data capture form designed for this purpose (REDCap) (Harris et al., 2009). Extracted data included the following: first author, publication year, journal, funding source, study objective, study design, geographic location, study dates, details of exposed and reference populations (sampling method, sample sizes, % response rate), exposure measurements (types, data sources, dates), covariables (types, data sources, dates), health outcome measurements (types, data sources, dates), statistical analysis methods, and effect estimates (including 95% confidence intervals (CI) or *p*-values) as reported by authors in their respective publications. When an element of a study was unclear, the corresponding author was contacted for clarification.

### Data synthesis

Given the variation in exposure and outcome definitions, data synthesis was descriptive. We followed PRISMA guidelines for scoping reviews (Tricco et al., 2018). First, we produced a high-level summary of all reviewed studies (first author, study setting, study population and time period, population source and sampling method, exposure measures, outcome measures, and study findings) grouped by hierarchy of epidemiologic study design (retrospective cohort, case–control, cross-sectional, and ecologic) and alphabetical order of first author. We identified a study as having a significant effect (harmful or protective) for a given health outcome when there was a statistically significant association reported between one or more exposure levels and one or more health outcomes (i.e., a reported *p*-value < 0.05 and/or effect estimate where the 95% CI did not cross one for relative risk or zero for absolute risk).

We further grouped studies by health outcomes, and calculated summary statistics when two or more studies reported on the same health outcome. The direction of effect estimates for each outcome is summarized descriptively in Table 2 using arrows, including the proportion of studies reporting harmful and protective effects for a given outcome, and the number of distinct geographic settings in which these outcomes were studied. Supplementary materials provide detailed descriptions of each study’s exposure and outcome measurement, covariates, data sources, analytic approach, main study results, and conclusions as reported by authors in the abstract (Online Resources 2–3). For the sake of brevity, outcomes less extensively studied were also described in more detail in supplementary material (Online Resource 4).

## Synthesis

After screening 3980 titles and abstracts, 52 studies met our inclusion criteria (Fig. 1). Two studies (Cairncross et al., 2022; Caron-Beaudoin et al., 2021) were set in Canada (British Columbia, Alberta), and the remainder were from US states including California, Colorado, New York, Ohio, Oklahoma, Pennsylvania, and Texas, with two spanning multiple states (Hu et al., 2022; Li et al., 2022). One study was industry-funded (Fryzek et al., 2013), and the majority (*n* = 38, 73%) were published after 2017 (Table 1).

**Table 1:** Characteristics of included epidemiologic studies, by study design

	Author, publication year	Country (state or province) and study population size	Study population and time period	Population source and sampling method	Exposure measure(s)	Health outcome(s)	Study findings of exposed vs unexposed
Cohort studies							
1	Apergis et al., 2019	US (OK) 556,794	Newborns 2006–2017	Birth records	Well count	Birth outcomes	Decreased TBW Increased LBW Poorer infant health index
2	Cairncross et al., 2022	Canada (AB) 26,193	Maternal-infant pairs in rural AB 2013–2018	Health services records	Well density, well count, distance to nearest well	Birth outcomes	Increased spontaneous PTB, SGA, major congenital anomalies, fetal infant mortality (suggestive)
3	Caron-Beaudoin et al., 2021	Canada (BC) 5018	Maternal-infant pairs in Fort St. John 2006–2016	Birth records	IDW	Birth outcomes	Decreased TBW Increased PTB No effect, SGA, head circumference
4	Casey et al., 2016	US (PA) 10,496	Maternal-infant pairs 2005–2013	Electronic health record files including labor and delivery notes, and labor and delivery database maintained by nursing personnel	IDW incorporating phase	Birth outcomes	Increased PTB No effect SGA, TBW, Apgar
5	Currie et al., 2017	US (PA) 1,125,748	Newborns 2004–2013	Birth records	Proximity to well	Birth outcomes	Decreased TBW Increased LBW Poorer infant health index
6	Cushing et al., 2020	US (TX) 23,487	Maternal-infant pairs in rural areas 2004–2013	Department of State health services records	Number of nightly flares, IDW sum of flares, total flared area, well count	Birth outcomes	Flares: Increased PTB Shorter gestation No effect SGA TBW Wells: Increased PTB Shorter gestation Decreased TBW No effect SGA
7	Hill, 2018	US (PA) 21,610	Maternal-infant pairs 2003–2010	Birth records (restricted-access version)	Proximity to well	Birth outcomes	Decreased TBW, Apgar, poorer health index Increased LBW, PTB, SGA No effect gestation period, PTB, congenital anomaly
8	Hill and Ma, 2022	US (PA) 325,439	Maternal-infant pairs residing within 10 km of active UNGD drilling 2003–2015	Birth records	Number of wells drilled during gestation period at different distances from the community water source	Birth outcomes	Decreased TBW, gestational length Increased LBW, PTB
9	Janitz et al., 2019	US (OK) 476,600	Newborns 1997–2009	Birth defects registry	IDW	Birth outcomes	Increased neural tube defects (suggestive) No effect critical congenital heart defects, oral clefts
10	Li et al., 2022	US—all UOGD regions 5,198,496	Seniors 65 + years 2001–2005	US Medicare beneficiary data	IDW of active wells and downwind exposure	All-cause mortality	Increased mortality
11	McKenzie et al., 2014	US (CO) 124,842	Newborns in rural CO 1996–2009	CO Responds to Children with Special Needs birth registry; hospital records; the Newborn Genetics Screening Program; the Newborn Hearing Screening Program; laboratories, physicians, and genetic, developmental, and other specialty clinics	IDW	Birth outcomes	Increased TBW Decreased LBW Decreased PTB Increased neural tube defects, congenital heart defects No effect oral clefts
12	Stacy et al., 2015	US (PA) 15,451	Maternal-infant pairs in 3 counties 2007–2010	Birth records	IDW	Birth outcomes	Decreased TBW Increased SGA No effect PTB
13	Tran et al., 2020	US (CA) 2,918,089	Maternal-infant pairs 2006–2015	Birth records	Inactive well count & well production volume	Birth outcomes	For rural population: Decreased TBW Increased LBW, SGA Increased PTB (suggestive)
14	Tran et al., 2021	US (CA) 979,961	Maternal-infant pairs in 8 counties	Birth records	Well density during stimulation (fracking) phase	Birth outcomes	For rural populations: Increased LBW, SGA Decreased TBW No effect PTB Urban populations: Increased SGA Decreased TBW (suggestive) Decreased PTB Decreased LBW (suggestive)
15	Whitworth et al., 2017	US (TX) 158,894	Maternal-infant pairs 2010–2012	State health services records	IDW	Birth outcomes	Increased PTB, fetal death No effect SGA, TBW
16	Willis et al., 2021	US (TX) 2,598,025	Maternal-infant pairs 1996–2009	Birth records	Distance to nearest active well	Birth outcomes	Decreased TBW No effect SGA
17	Willis et al., 2022	US (TX) 2,845,144	Maternal-infant pairs 1996–2009	Birth records	Distance to nearest active well	Maternal health outcomes	Increased gestational hypertension and eclampsia for women
18	Willis et al., 2023	US (TX)	Maternal-infant pairs 1996–2009	Vital Statistics, & Birth Defects Registry	IDW of active wells, oil, gas, and wastewater production monthly average and distance to nearest well within 10 km	Birth outcomes Birth defect—9 categories, any anomaly, and 1 + anomaly	Increased for any birth defects, strongest increase for cardiac and circulatory defects
	Case control studies						
19	Clark et al., 2022	US (PA)	Children ages 2–7 years with acute lymphocytic leukemia matched to healthy controls on birth year	Cancer registry (cases) and birth records (controls)	IDW and a water pathway-specific proximity metric	Acute lymphocytic leukemia	Increased acute lymphocytic leukemia
20	Elser et al., 2021	US (Northern CA)	Prevalent migraine cases matched to controls 2014–2018	Sutter Health electronic health records database	IDW from 60 methane super-emitters, and active oil and gas, average annual and PM_2.5_, NO_2_ emissions	Migraine and migraine severity	Increased migraine with methane emissions, no effect with oil and gas wells Increased severity of migraines with PM_2.5_
21	Koehler et al., 2018	US (PA) 35,508	All asthmatic patients 2005–2012	Geisinger Health System electronic medical records: mild asthma exacerbations (cases) matched to no exacerbations (controls)	Proximity to well IDW incorporating phase, compressors, impoundments and flares	Asthma exacerbation	Increased asthma exacerbations
22	McAlexander et al., 2020	US (PA) 12,330	All heart failure patients 2008–2015	Geisinger Health System electronic medical records for heart failure: hospital admissions for heart failure (cases) matched to no hospital admissions (controls)	IDW, phase specific	Heart failure hospital admissions	Increased heart failure admissions
23	McKenzie et al., 2017	US (CO) 665	Children aged 0–24 years diagnosed with cancer 1991–2013	Central Cancer Registry: lymphocytic leukemia or non-Hodgkin’s lymphoma diagnosis (cases) matched to other cancer diagnosis (controls)	IDW	Childhood cancer	Increased acute lymphocytic leukemia No effect non-Hodgkin’s lymphoma
24	McKenzie et al., 2019a	US (CO) 3329	Newborns 2005–2011	Colorado Responds to Children with Special Needs registry: newborns with 3 types of congenital heart defects (cases), matched to birth records of healthy newborns (controls)	IDW	Birth outcomes	Increased congenital heart defects, pulmonary artery valve defects Rural population: Increased aortic artery and valvular defects, aortic artery and valve defects, conotruncal defects, tricuspid valve defects
25	Rasmussen et al., 2016	US (PA) 60,373	Asthma diagnosis 2005–2012	Geisinger Health System electronic medical records: asthma exacerbation (cases) matched to no exacerbations (controls)	IDW, phase specific	Asthma exacerbation	Increased asthma exacerbations
26	Tang et al., 2021	US (TX) 695,354	Newborns 1999–2011	Birth defects registry (cases) matched to birth records of healthy newborns (controls)	Well density	Birth outcomes (neural tube defects, congenital heart defects)	Increased spina bifida, anencephaly, aortic valve stenosis, hypoplastic left heart syndrome, pulmonary valve atresia or stenosis, gastroschisis No effect oral clefts
27	Walker Whitworth et al., 2018	US (TX) 81,294	Pregnant women 2010–2012	Birth records: PTB (cases) matched to term births (controls)	IDW, phase specific	Birth outcomes	Increased PTB
	Cross-sectional studies						
28	Blinn et al., 2020	US (PA) 104	Residents in southwestern PA 2012–2017	Self-reported health convenience sample survey	IDW, cumulative well density, annual well emissions concentration	Self-reported health symptoms	Increased total symptoms (mainly upper respiratory; neurological and muscular)
29	Brown et al., 2019	US (PA) 104	Residents in southwestern PA 2012–2017	Self-reported health convenience sample survey	Air emission of CO, NOx, PM2.5, VOCs and number of air emission sources	Self-reported health symptoms	Increased cough and any respiratory symptom with emission sources No association with air emissions
30	Elliott et al., 2018	US (OH) 66	Residents in highly fracked areas 2016	Interviewer-administered questionnaire, volunteer participants	Proximity to well, IDW; presence of volatile organic compounds, disinfection by-products, gasoline 7 diesel range organics in drinking water	Self-reported health symptoms	Increased general health symptoms (including stress, fatigue)
31	Johnston et al., 2021	US (CA)	Individuals surveyed from 2 communities Jan 2017–Aug 2019	Volunteers recruited using community health workers	Residence in community with active (vs inactive) wells, proximity to and downwind to active wells	Self-reported respiratory symptoms	Increased wheeze Decreased forced expiratory volume in the first second, forced vital capacity
32	Mayer et al., 2021	US (CO) 890	Individuals surveyed from 3 communities with no UNGD; a lot of UNGD; and permitted wells with little active drilling 2015–2016	Paper-based survey mailed to individuals	Communities with varying levels of UNGD activity (none, some, high)	Self-rated health	Decreased self-rated health
33	McKenzie et al., 2019b	US (CO) 97	Adults living in Northeastern CO 2015–2016	Interviewer-administered questionnaire, biometric and lab testing volunteer participants	IDW	Cardiovascular outcomes	Increased augmentation index, systolic blood pressure, diastolic blood pressure, interleukin-1β, tumour necrosis factor-α
34	Rabinowitz et al., 2015	US (PA) 492	Residents in rural PA 2013	Interviewer-administered health survey, random selection of sample frame	Proximity to well	Self-reported health symptoms	Increased total, skin, and upper respiratory symptoms No effect other respiratory, neurological, cardiovascular, or GI symptoms
35	Steinzor et al., 2013	US (PA) 108	Residents in 55 households 2011–2012	Self-reported health survey, snowball and network sampling	Proximity to well, compressor, or impoundment structure	Self-reported health symptoms and conditions	Increased upper respiratory, cough, skin, headaches, anosmia, joint swelling, and epistaxis symptoms No effect other musculoskeletal system, shortness of breath, GI, fatigue, or memory symptoms
36	Tustin et al., 2017	US (PA) 7785	Adults 2014	Geisinger Clinic Health System: Self-reported health survey, randomly sampled from source study population, oversampled for racial/ethnic minority status and higher likelihood of chronic rhinosinusitis	IDW incorporating phase	Self-reported health symptoms	Increased chronic rhinosinusitis plus migraine, chronic rhinosinusitis plus fatigue, migraine plus fatigue, all three outcomes together
	Ecologic studies						
37	Apergis et al., 2021	US (OK) 590,780	Residents in 76 counties 1998–2017	Birth records	Number of drilled unconventional oil and gas wells per county	Mortality, cancer, cardiac and heart disease rates, life expectancy	Increased mortality, cancer, heart failure, and respiratory disease rates Decreased life expectancy
38	Busby and Mangano, 2017	US (PA) 82,558	Newborns in 10 counties with most UNOG activity compared to all other counties 2003–2006 and 2007–2010	Birth records	Well count before and after UNOG expansion	Birth outcomes	Increased early infant mortality
39	Bushong et al., 2022	US (PA)	Residents in 62 counties 2001–2014	Department of Health hospital admission rates	Well density	Hospital admissions rates for asthma	Increased hospitalization rates for asthma
40	Denham et al., 2019	US (PA)	Residents in 67 counties 2003–2014	Inpatient records	Well count (recently drilled, cumulative) and well density	Hospital admissions	Increased hospital admissions for skin conditions, UTI, ureter stones, pyelonephritis in women aged 20–64 years No effect for other causes
41	Denham et al., 2021	US (PA and New York) 47 counties in Penn (w/ UNGD activity), and 24 counties in New York (w/o UNGD activity)	Residents of PA and New York, 2005–2014	Inpatient discharge records from the PA Health Care Cost Containment Council, all included	Well count (in 1 year and cumulative) and well density	Cardiovascular (AMI hospital admissions and deaths)	Increased hospital admissions for AMI Increased AMI-related deaths
42	Erickson et al., 2022	US (CO) 252,505	Maternal–infant pairs 1999–2019	Birth records	Well density and production volume	Births outcomes	Increased prematurity with production volume Increased birthweight with well density and production
43	Finkel, 2016	US (PA) 1,031,953	Residents in 6 counties living with high, moderate, and minimum UNOG activity 2000–2004 2004–2008 2008–2012 (respectively)	Cancer Registry	Well count	Cancer	Increased bladder cancer No effect thyroid cancer and leukemia
44	Fryzek et al., 2013	US (PA)	Children in 67 counties 1990–2009	Cancer Registry	Well count	Cancer	Increased central nervous system tumours No effect total childhood cancers or childhood leukemia
45	Hu et al., 2022	US (49 states) 2010 (40.3 million) 2019 (54.1 million)	Residents ≥ 65 years in 24 fracking and 25 non-fracking states 2010–2018	US Census, US Centers for Disease Control and Prevention	Regression modelled exposure (Annual Loss Expectancy)	> 65 years stroke-related mortality	Increased stroke mortality in most states
46	Jemielita et al., 2015	US (PA) Population 157,526	Residents in 3 counties, 2 with increased UNGD activity and 1 country with no UNGD activity, total (67 zip codes) 2007–2011	Inpatient records	Well count and well density	Hospital admissions (all-cause; 25 cause-specific health categories)	Increased cardiology, neurology No effect other cause-specific categories
47	Ma et al., 2016	US (PA) 1,401,813	Newborns 2003–2012	Birth records	Proximity to well by zip code and well density	Birth outcomes	No effect birth defects
48	Makati et al., 2022	US (VA)	Residents of Monongalia County, West Virginia 1990–2019	All individuals diagnosed with ANCA (anti-nuclear cytoplasmic auto-antibodies) vasculitis	Natural gas production before and after 2010	County prevalence of ANCA before and after 2010	Increased ANCA prevalence after 2010
49	Peng et al., 2018	US (PA)	Residents aged 5 + years in 39 counties with UNGD and 28 counties without 2001–2013	Inpatient records	Proximity to well and production volume	Hospital admissions for specific diagnoses	Increased pneumonia (elderly), AMII, COPD, asthma (younger age groups)
50	Schuele et al., 2022	US (28 states)	Maternal-infant pairs in counties with any gas production 2005–2018	Birth records	IDW gas-producing wells	Birth outcomes	Decreased TBW Increased LBW, SGA Increased gestational age, decreased PTB
51	Willis et al., 2018	US (PA) 15,837 hospital admissions	Children aged 2–18 years living in 571 rural zip codes fully located on the Marcellus Shale 2003–2014	Health services inpatient records	Proximity to well, recently drilled and cumulative well count, reported UNGD air pollutants	Pediatric asthma hospital admissions	Increased pediatric hospital admissions
52	Willis et al,, 2020	US (TX) 24,333 (unexposed) and 48,589 (exposed) hospital admissions	Children aged 1–17 years living in 1249 zip codes fully located on a shale play or basin 2000–2010	Health services inpatient records	Cumulative well density stratified by conventional and unconventional, gas production, flaring volumes	Pediatric asthma hospital admissions	Increased pediatric asthma hospital admissions with production volumes Inconsistent effect with flaring

^*^AB, Alberta; *BC*, British Columbia; *CA*, California; *CO*, Colorado; *OH*, Ohio; *OK*, Oklahoma; *PA*, Pennsylvania; *TX*, Texas; *US*, United States; *WV*, West Virginia

^**^Outcome reported as having an effect if effect estimate is statistically significant (i.e., a reported *p*-value < 0.05 and/or effect estimate where the 95% CI did not cross one for relative risk or zero for absolute risk); and reported as being suggestive of an effect when there is evidence of a trend but not statistically significant (i.e., a* p*-value was between 0.05 and 0.1, and/or the lower CI was between 0.9 and 1.0, or authors reported a large effect magnitude)

*AMI*, acute myocardial infarction; *COPD*, chronic obstructive pulmonary disease; *GI*, gastrointestinal; *IDW*, inverse distance weighting; *LBW*, low birthweight; *NO*_*2*_, nitrogen dioxide; *PM*, particulate matter; *PTB*, preterm birth; *SGA*, small for gestational age; *TBW*, term (average) birthweight; *UNGD*, unconventional natural gas development; *UOGD*, unconventional oil and gas development; *UTI*, urinary tract infection; *vs*, versus

Many studies used a cumulative exposure measure of UOGD activity based on number of active wells weighted by proximity to residence (inverse distance weighted [IDW]) within defined geographic radius/buffer zones (Table 1; Online Resource 2). More recent studies further refined the IDW measure by development phase, well depth, and production volumes (Elliott et al., 2018; Koehler et al., 2018; McAlexander et al., 2020; Rasmussen et al., 2016; Tang et al., 2021; Tustin et al., 2017; Walker Whitworth et al., 2018). Several studies incorporated upwind/downwind or uphill/downhill directionality for air and water exposure measurement (Hill & Ma, 2022; Johnston et al., 2021; Li et al., 2022). Some researchers included flaring events (Cushing et al., 2020; Koehler et al., 2018; Willis et al., 2020), compressor engine activity (Koehler et al., 2018), and conventional oil and gas extraction as separate exposure covariates (Apergis et al., 2021; Elser et al., 2021; Schuele et al., 2022; Willis et al., 2020), or examined annual air (Apergis et al., 2021; Blinn et al., 2020; Brown et al., 2019; Hill, 2018; Hill & Ma, 2022; Hu et al., 2022; Li et al., 2022; McKenzie et al., 2017, 2019a; Tran et al., 2021; Willis et al., 2018) and water contamination (Hill, 2018; Hill & Ma, 2022) alongside UOGD exposure metrics and/or model covariates. Two studies directly measured drinking water and/or air pollutants at participants’ residences (Elliott et al., 2018; Steinzor et al., 2013) (Table 1; Online Resource 2).

Outcome and covariate data were drawn from secondary administrative/clinical health records, registries, laboratory markers, biometrics, and self-report (Table 1; Online Resource 3). Health outcomes for epidemiologic studies included birth-related (fetal growth, preterm birth, birth deformities, etc.), respiratory (predominantly asthma), and cardiovascular outcomes, cancer, self-reported symptoms, all-cause/cause-specific hospitalizations, and mortality (Tables 1 and 2; Online Resource 3).

### Birth outcomes

#### Fetal growth

Fetal growth measures were the most studied outcomes in relation to UOGD exposure. Many studies reported lower average birthweight (as a continuous variable) (Apergis et al., 2019; Caron-Beaudoin et al., 2021; Currie et al., 2017; Cushing et al., 2020; Hill, 2018; Hill & Ma, 2022; Schuele et al., 2022; Stacy et al., 2015; Tran et al., 2020, 2021; Willis et al., 2021) and low birthweight (as a categorical variable; term birthweight < 2500 g) (Apergis et al., 2019; Currie et al., 2017; Hill, 2018; Hill & Ma, 2022, 2022; Schuele et al., 2022; Tran et al., 2020, 2021) (Table 2). Almost all fetal growth studies applied cohort or case–control study designs and are described in greater detail below (Tables 1 and 2; Online Resource 3).

**Table 2:**
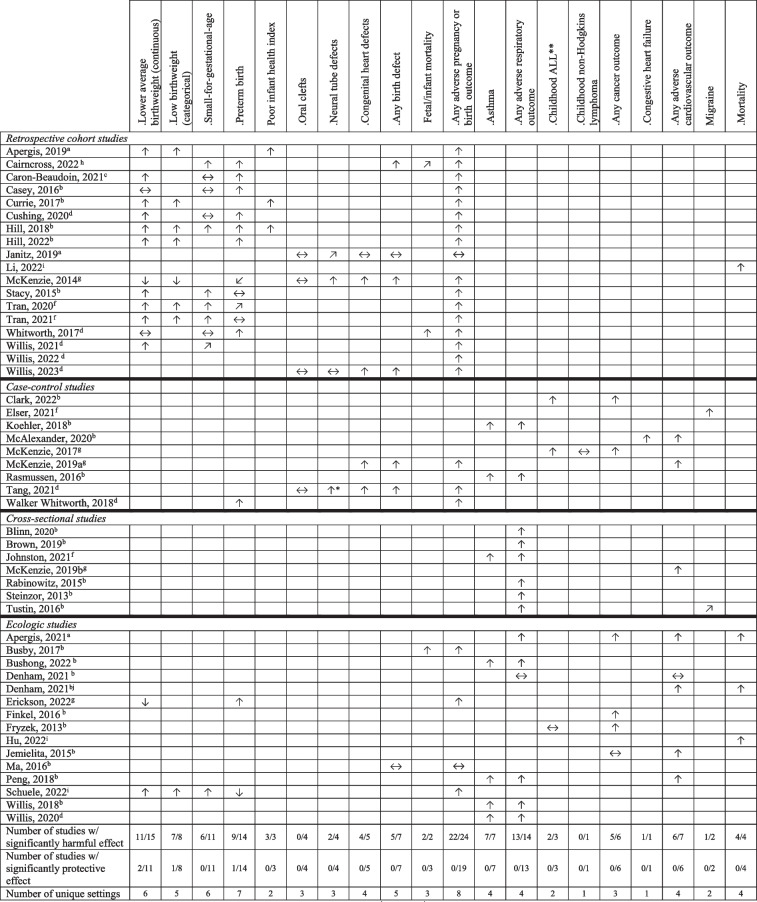
UNGD activity and health outcomes grouped by similar outcome measures and design

^a^Oklahoma; ^b^Pennsylvania; ^c^British Columbia; ^d^Texas; ^e^Ohio; ^f^California; ^g^Colorado; ^h^Alberta; ^i^US states; ^j^New York

^†^All studies are survey of self-reported symptoms, grouped by symptom type (except McKenzie et al., 2019b)

^§^Hospitalization rates grouped by diagnostic categories

^*^Anencephaly and spina bifida examined

^**^ALL: acute lymphoblastic leukemia

^***^ “Any adverse” includes study outcomes included in the columns and others not included and falling under the same category

↑ = statistically significant increased risk; ↓ = statistically significant decreased risk; ↗/↘ = suggestive of an effect but non-significant (i.e., a *p*-value was between 0.05 and 0.1, and/or the lower CI was between 0.9 and 1.0, or authors reported a large effect magnitude); ↔  = nonsignificant

A cohort study in Oklahoma (> 500,000 newborns) reported a progressive decrease in birthweight the closer an individual lived to UOGD wells (Apergis et al., 2019). Similarly, a large study in Pennsylvania (> 1,000,000 newborns) reported a 38 g decrease in birthweight associated with residence within 1 km of a well during pregnancy (Currie et al., 2017). These results were consistent with another study in Pennsylvania that reported an inverse association of birthweight with UOGD-related contamination of public drinking water sources (Hill & Ma, 2022), after adjustment for individual-level socioeconomic status (SES) variables, smoking status, month and year of birth, and child sex (Hill & Ma, 2022). A cohort study in British Columbia detected lower birthweights with increased exposure to oil and gas wells; the associations were only significant in the second or third quartiles (and not the fourth quartile) compared to the first (Caron-Beaudoin et al., 2021). The authors discuss this pattern as a possible non-linear non-monotonic dose response related to endocrine disruption. This study did not include SES as a covariate which could have led to some bias in effect estimates due to confounding.

Casey et al. (2016) used electronic clinical health records to study fetal growth outcomes in Pennsylvania and adjusted for a wide range of potential confounders (including primary care provider status, smoking status during pregnancy, pre-pregnancy BMI, parity, antibiotic orders during pregnancy, and receipt of medical assistance). The authors detected a decrease in birthweight in the highest quartile of exposure compared to the first (Q4 versus Q1, *β* − 31 g, 95% CI − 57, − 5) that lost statistical significance after adjusting for year of birth (Q4 versus Q1, *β* − 20 g, 95% CI − 15, 16). Whitworth et al. (2017) examined these outcomes in Texas also adjusting for a wide range of clinical confounders (including pre-pregnancy BMI, adequacy of prenatal care utilization, and previous poor pregnancy outcome) and reported no effect between exposed and unexposed in adjusted models.

There were methodological differences in the treatment of gestational age. For example, while some only included birthweight data for term births (≥ 37 weeks gestation) in their models (Caron-Beaudoin et al., 2021; Casey et al., 2016; Currie et al., 2017; Stacy et al., 2015; Tran et al., 2020; Willis et al., 2021), others adjusted for gestational age as a covariate (Cushing et al., 2020; Erickson et al., 2022; Hill, 2018; McKenzie et al., 2014; Schuele et al., 2022; Willis et al., 2021), while others did not account for gestational age in their models (Hill & Ma, 2022; Whitworth et al., 2017). Despite these differences in exposure metrics, control groups, and statistical models, a majority of studies identified a decrease in birthweight with UOGD exposure.

Proximity to UOGD activity was also associated with low birthweight (< 500 g) in a majority of studies examining this outcome (Table 2). In contrast, one study set in Colorado reported an increase in average birthweight and decreased odds of low birthweight associated with higher UOGD exposure (McKenzie et al., 2014). The authors of the study pointed to the lack of adjustment of SES, prenatal care, and pregnancy complications that may explain these discordant results.

Small for gestational age (SGA) (birthweight < 10th percentile for gestational age) was another frequently examined outcome. In a cohort study in rural Alberta, Canada, living within 10 km of one or more wells was associated with an increased risk ratio (RR) of SGA (RR 1.12, 95% CI 1.03–1.23) (Cairncross et al., 2022). To better account for well density, the study also examined the risk of living within 10 km of > 100 wells compared to 1–24 wells and reported a higher risk ratio of SGA (RR 1.65, 95% CI 1.10–2.48). However, certain individual-level factors such as smoking and SES were not included as covariates because these variables were unavailable in the provincial administrative dataset. Five other studies (Hill, 2018; Schuele et al., 2022; Stacy et al., 2015; Tran et al., 2020, 2021) reported an association between UOGD proximity and SGA (Table 2). Cohort studies in British Columbia (with > 5000 women) (Caron-Beaudoin et al., 2021) and Pennsylvania (with > 10,000 women) (Casey et al., 2016) and two studies in Texas with > 23,000 (Cushing et al., 2020) and > 150,000 (Whitworth et al., 2017) women reported no association between UOGD exposure and SGA.

#### Preterm birth

Several studies reported a significant association with UOGD and preterm birth (Cairncross et al., 2022; Caron-Beaudoin et al., 2021; Casey et al., 2016; Cushing et al., 2020; Erickson et al., 2022; Hill, 2018; Hill & Ma, 2022; Walker Whitworth et al., 2018; Whitworth et al., 2017) (Tables 1 and 2; Online Resource 3). The magnitude of adjusted ORs ranged between 1.11 and 2.00 with trend-tested (Cushing et al., 2020; Walker Whitworth et al., 2018) and descriptive evidence of positive linear trends across increasing exposure categories (Casey et al., 2016).

In rural Alberta, spontaneous preterm birth (Cairncross et al., 2022) was associated with living within 10 km of > 100 wells compared to women living within 10 km of 1–24 wells (OR 1.64, 95% CI 1.04–2.60). UOGD activity exposure was also associated with increasing OR of preterm birth by increasing exposure levels in Pennsylvania (quartile (Q) 2: OR 1.2, 95% CI (0.9–1.6); Q3: OR 1.3, 95% CI (1.0–1.7); Q4: OR 1.4, 95% CI (1.0–1.9)) (Casey et al., 2016) and Texas (tertile (T) 1: OR 1.02, 95% CI (0.96–1.08); T2: OR 1.13, 95% CI (1.06–1.20); T3: OR 1.15, 95% CI (1.08–1.22), within 10 mile buffer) (Whitworth et al., 2017). Preterm birth was also associated with water quality compromised by UOGD-implicated chemicals (Hill & Ma, 2022). In British Columbia, there was an increased odds of preterm birth among women living in the second quartile of UOGD exposure (OR 1.60, 95% CI 1.30–2.43), but not in the third or fourth exposure quartiles (Caron-Beaudoin et al., 2021).

In contrast, McKenzie et al. reported a decreased odds of preterm birth, in line with a protective effect on low birthweight (McKenzie et al., 2014). This study did not adjust for SES (described earlier). A large ecologic study across 28 US states also reported a decreased risk of preterm birth with exposure to UOGD (Schuele et al., 2022). Exposure to wells was not significantly associated with preterm birth in rural populations in California (OR 1.17, 95% CI 0.64–2.12), and was associated with a decrease in preterm birth in urban populations (OR 0.65, 95% CI 0.48–0.87) (Tran et al., 2021). Notably, while UOGD does take place in California, most wells are a result of conventional oil and gas production.

One study looking at preterm birth by UOGD phase reported that the drilling phase–specific IDW yielded stronger associations with preterm birth compared to the production-specific phase (Walker Whitworth et al., 2018). The authors further found that the greatest risk for extreme preterm birth (< 28 weeks) was associated with residence in the top third of UOGD activity (OR 2.00 (1.23–3.24) and 1.53 (1.03–2.27)) for drilling and production, respectively (Walker Whitworth et al., 2018). A study in Texas (Cushing et al., 2020) reported the association between UOGD flaring and preterm birth was only significant with high (OR 1.50, 95% CI 1.23–1.83) but not low flaring (OR 0.82, 95% CI 0.61–1.04). A strength of this study was its subgroup analysis by race; a disproportionate exposure to flaring was identified in Hispanic populations.

#### Other maternal/infant health outcomes

One cohort study (McKenzie et al., 2014) and one case–control study (Tang et al., 2021) found increased odds of neural tube defects associated with UOGD exposure. Another case–control study reported increased odds of congenital heart defects (McKenzie et al., 2019a). A large retrospective study reported increased risk of all congenital abnormalities (Willis et al., 2023), while another reported no effect of UOGD on birth defects (Ma et al., 2016) (Tables 1 and 2). One study reported an association of gestational hypertension and eclampsia with residential proximity to UOGD activity using a difference in differences approach allowing for counterfactual comparisons (Willis et al., 2022). Further details on these studies are provided in Online Resource 4.

### Asthma and other respiratory outcomes

Several studies reported significant associations between UOGD activity and asthma (Table 2). A case–control study in Pennsylvania examined the association of phase-specific exposure activity metrics and asthma exacerbations by severity level (mild, moderate, and severe) (Rasmussen et al., 2016). The authors reported higher risk of all types of asthma exacerbation irrespective of exposure phase. The magnitude of OR for mild asthma exacerbations and the production phase was 4.4 (95% CI 3.8–5.2) and most of the models described linear dose–response patterns across increasing exposure quartiles. Another case–control study in Pennsylvania (using the same dataset but incorporating the four phases of well development and UOGD-related compressor engines) reported an adjusted OR (95% CI) of 3.69 (3.16–4.30) for asthma mild exacerbations and residential location in the highest UOGD activity quartile compared to the lowest, after adjustment by several individual-level covariates, compressor station activity (air pollution surrogate), weather estimates, and community-based socioeconomic measures (Koehler et al., 2018).

A cross-sectional study in California compared lung capacity measures and self-reported wheezing between residents living within 1000 m of an active oil well versus an idle well, and living near (< 200 m) an active well versus further away (> 200 m) (Johnston et al., 2021). The odds of wheezing increased among those living near an active versus idle well (OR 2.58, 95% CI 1.19–5.59), but was not significantly increased for those living near compared to those living further away. However, there was a consistent decrease in forced expiratory volume during first second (FEV1) and forced vital capacity (FVC) measures regardless of the reference group. The study was also unique in that it examined the impact of living upwind or downwind of a drilling well, showing a decrease in FEV1 and FVC among those living downwind and less than 200 m compared to those living upwind and more than 200 m from wells.

In addition to asthma, two ecological studies reported significantly higher pneumonia hospitalizations among seniors (Peng et al., 2018) and asthma-related hospitalization rates (Bushong et al., 2022). Other outcomes from cross-sectional symptom survey studies include self-reported upper (Blinn et al., 2020; Brown et al., 2019; Rabinowitz et al., 2015; Steinzor et al., 2013; Tustin et al., 2017) and lower respiratory symptoms (Steinzor et al., 2013) associated with UOGD exposure.

### Cancer outcomes

Two case–control studies examined acute childhood lymphocytic leukemia (Clark et al., 2022; McKenzie et al., 2017) (Tables 1 and 2). Both studies reported increased effect estimates associated with UOGD exposure (OR 4.3, 95% CI 1.1–16 (McKenzie et al., 2017) and OR 2.80, 95% CI 1.11–7.05 (Clark et al., 2022)). The latter study suggested that the preconception to birth exposure window may be especially important. Details of other ecologic studies examining UOGD and cancer outcome (Apergis et al., 2021; Finkel, 2016; Fryzek et al., 2013; Jemielita et al., 2015) are provided in Tables 1 and 2 and Online Resources 2–4.

### Cardiovascular and cerebrovascular outcomes

A case–control study with 12,330 participants in Pennsylvania (McAlexander et al., 2020) reported a significant association with heart failure hospitalizations. Additionally, a cross-sectional study (McKenzie et al., 2019b) and ecological studies reported associations with cardiovascular (Apergis et al., 2021; Denham et al., 2021; Jemielita et al., 2015; Peng et al., 2018) and cerebrovascular outcomes (Hu et al., 2022). Further details on these studies are provided in Online Resource 4.

### Self-reported symptoms

Cross-sectional survey studies from Ohio, Colorado, and Pennsylvania identified associations between residential UOGD proximity and self-reported health symptoms, including respiratory, dermal, and neurological symptoms (Blinn et al., 2020; Elliott et al., 2018; Johnston et al., 2021; Mayer et al., 2021; Rabinowitz et al., 2015; Steinzor et al., 2013; Tustin et al., 2017). Further details on these studies are provided in Online Resource 4.

### Hospital admissions

In addition to respiratory, oncologic, and cardiovascular outcomes, UOGD proximity was associated with higher hospitalization rates for neurologic (Jemielita et al., 2015), urologic (Denham et al., 2021; Jemielita et al., 2015), dermatologic (Denham et al., 2021; Jemielita et al., 2015), and auto-immune conditions (Makati et al., 2022). The studies reporting these outcomes were ecological in nature and therefore more subject to internal bias and confounding.

### Mortality

Evidence from cohort and ecological studies suggests increased mortality rates among populations living proximal to various UOGD exposure measures (Apergis et al., 2021; Denham et al., 2021; Hu et al., 2022; Li et al., 2022). Further details on these studies are provided in Online Resource 4.

## Discussion

This review includes 52 studies of which over half were not included in previous reviews (Bamber et al., 2019; Deziel et al., 2020). Almost one half examined the association of living in proximity to UOGD and birth outcomes, with many using cohort and case–control study designs in a variety of settings. Other studies examined respiratory outcomes, cardiovascular outcomes, cancer, self-reported symptoms, hospitalizations, and mortality. Studies are set in a growing number of diverse geographic regions in the United States and two regions in Canada. Overall, the studies suggest evidence of detrimental health effects related to living in proximity to UOGD. However, some knowledge gaps remain.

To the best of our knowledge, this review is the first published on this topic with a stated goal to focus on Canadian studies. Despite a number of published biomonitoring studies (Caron-Beaudoin et al., 2018, 2019, 2022; Claustre et al., 2023), we only identified two Canadian epidemiologic studies that met our study inclusion criteria. This “evidence of absence” is concerning given the country’s almost 20-year history of UOGD, the industry’s continued expansion, and the wells’ frequent location on the territories of Indigenous communities already disproportionately impacted by health and economic disparities due to the ongoing effects of colonization (FNHA, 2018).

Our review builds on prior published reviews on this topic (Bamber et al., 2019; Deziel et al., 2020). Like Bamber et al., we restricted our search to outcomes more likely related to chemical causal pathways. Those authors reviewed 20 studies and concluded that despite study limitations, there were modest findings of adverse health impacts with several studies focusing on birth outcomes. In a scoping review published one year later, Deziel et al. (2020) reviewed 29 articles, excluding outcomes based on self-report but including outcomes related to non-chemical causal pathways (sexually transmitted infections (STIs) and mental health outcomes). They concluded that the available research points to a growing body of evidence of health effects in communities living in proximity to oil and gas development (Deziel et al., 2020).

Our review identified a number of studies reporting adverse effects of UOGD exposure on birth outcomes, most of which were retrospective longitudinal cohort or case–control studies, reducing concerns of reverse causation. Both impaired fetal growth and preterm birth have been associated with adverse cardiovascular, metabolic, neurodevelopmental, and respiratory sequelae in later life (Crump, 2020). Although fewer in number, other cohort and case–control studies identified higher rates of neural tube, congenital heart defects, any congenital anomaly, lower infant health index, and fetal/infant mortality.

In addition to birth outcomes, an increasing number of studies report higher rates of asthma exacerbation. Considering the irritant gas emissions from UOGD, this association is not surprising. Further investigation of the most prevalent airway disease, chronic obstructive pulmonary disease (COPD), is warranted. Some evidence from case–control and cohort studies also suggest an increased risk of childhood acute lymphocytic leukemia, hospital admission for heart failure, and mortality. The relatively large effect estimates observed in case–control studies on cancer warrant further investigation in future studies despite the difficulties in examining the latent effects of UOGD exposure and cancer outcomes.

Most of the reviewed studies used surrogate exposure metrics, most commonly the IDW, due to the challenges of direct monitoring over large rural areas where UOGD is most common. This approach has been criticized for potential exposure misclassification (Wendt Hess et al., 2019). However, a growing number of studies are reporting correlation between IDW metrics and regional annual air pollutant emissions and/or UOGD-implicated chemicals in household and community reservoir water sources (Caron-Beaudoin et al., 2022, 2023; Claustre et al., 2023; Elliott et al., 2018; Hill & Ma, 2022). A recently published study linking water contaminants both to UOGD activity measures and to adverse birth outcomes (Hill & Ma, 2022) strengthens the evidence of a direct effect of UOGD exposure on adverse health outcomes. Cumulative exposures, as measured by the IDW approach, may be more reflective of “real life” exposure since these metrics capture aggregate exposure routes integrated over time. Exposure measurement is becoming increasingly sophisticated with more studies incorporating phase-specific metrics, flaring events, air and water pollution directional indicators, and adjustment for non-UOGD oil and gas development exposures in their models. Future exposure measurement should build on this multi-dimensional approach and also consider potential impacts of abandoned wells which have been identified as a growing concern (DiGiulio et al., 2023; Gross, 2023). Further examination of phase-specific contamination could better inform policies and regulations to protect communities from UOGD and other oil and gas development activities. Additionally, exposures associated with wildfire-triggered ignition of UOGD facilities (Cox, 2023; Gonzalez et al., under review – preprint available at https://eartharxiv.org/repository/view/6253/) and radiation exposure from NORMs (found in wastewater brine) need to be examined.

A majority of studies used health services administrative data sources and included all individuals (versus sampling) to define a study population. Reference populations for cohort studies were usually defined as those in the lowest UOGD exposure category compared to other higher levels of exposure categories. For case–control studies that examined potentially rarer events, the study populations were typically nested within a cohort of individuals with a defined condition (for example, heart failure or asthma exacerbations). Cases were defined as case events and compared with non-exacerbation control events. Most studies adjusted for demographics, SES, and relevant comorbidities: individual-level SES was often measured using level of education and/or receipt of medical assistance, and community-level SES was measured using various community deprivation indices. Individual SES may be an important confounder, and the lack of its adjustment in some studies is a limitation. A majority of studies included smoking status as a covariable (*n* = 30; Online Resource 3), and with the exception of cross-sectional survey studies, a majority of studies conducted sensitivity analyses (*n* = 30, data not shown). Studies varied in their measurement of known potential confounding variables, such as clinical data not usually available in administrative health records (for example, body size), geographical settings (urban versus rural), and other exposure variables (for example, ambient temperature) (Online Resource 3). Last, many reviewed studies used retrospective data collected for purposes other than research, making results prone to bias from missing data or misclassification bias that may have spuriously driven effect estimates away from or towards the null. Future prospective studies can help overcome the limitations related to retrospective observational studies.

Most US studies included race/ethnicity as population descriptors and adjusted for race and SES in their modeling. Fewer described the distribution of racialized populations across UOGD activity exposure levels or reported on the independent effects of these variables in their models. Some studies described potentially disproportionate exposures to UOGD among racialized groups (Cushing et al., 2020; Tran et al., 2020, 2021). Others explored effect modification by race (Cushing et al., 2020; Tang et al., 2021), with some evidence of higher effect magnitudes in Hispanic (Cushing et al., 2020), Black, and Asian (Schuele et al., 2022) populations. Few studies integrated community engagement methods into their study designs (Johnston et al., 2021; Steinzor et al., 2013) despite the increasingly recognized importance of grounding research processes in community-lived experience of Indigenous and other communities disproportionately affected by UOGD (Caron-Beaudoin & Armstrong, 2019; Garvie & Shaw, 2014; Hayward et al., 2021; Wing et al., 2008). No studies examined differential effects of UOGD in Indigenous populations. Future studies should consider the impact in systemically excluded populations in their research aims and methodological approach, and ensure meaningful engagement of affected communities throughout the research process.

The pathways linking UOGD exposure and health outcomes are still unclear. One hypothesized pathway is via increased exposure to environmental contaminants such as carbon monoxide (CO), nitrogen dioxide (NO_2_), particulate matter (PM_2.5_, PM_10_) (Ezani et al., 2018), and VOCs proximal to and downwind to areas from UOGD operations. VOCs have been detected at elevated environmental levels in the indoor tap water and air samples taken in the homes of pregnant women living proximal to UOGD drilling operations (Caron-Beaudoin et al., 2022). Exposure to these chemicals is known to induce cellular inflammation, oxidative stress, and alterations of placental tissue (Ferguson & Chin, 2017; Saenen et al., 2019), and has been implicated in lower neonatal birthweight in multiple studies (Stieb et al., 2012). Similarly, air pollutants such as CO, NO_2_, and PM_2.5_ can induce airway inflammation (Silbajoris et al., 2011) and oxidative stress on human airways (Thangavel et al., 2022).

Another hypothesized pathway is through endocrine disruption, or the ability of certain chemicals, even at extremely low levels, to block and/or mimic sex and thyroid hormones (Kassotis et al., 2016; Vandenberg et al., 2012), thereby potentially disrupting normal gestational age and labour onset. Endocrine disruption as a potential causal pathway has resulted in growing awareness of non-linear and non-monotonic dose–response relationships and the need for researchers to proactively recognize and characterize these in reporting of results (Vandenberg et al., 2012). Only one of the studies in our review made mention of non-linear dose response (Caron-Beaudoin et al., 2021).

A third hypothesized pathway is related to the mutagenic and carcinogenic properties of certain frack fluid chemicals (e.g., benzene, ethylene oxide), heavy metals (arsenic, beryllium), NORMs (Colborn et al., 2011; Xu et al., 2019), and air pollutants. These substances have been reported in higher concentrations in the air and water proximal to UOGD operations (Caron-Beaudoin et al., 2022; Garcia-Gonzales et al., 2019; Hill & Ma, 2022). Adding evidence for this pathway, three biomonitoring studies in British Columbia reported higher levels of a metabolite of benzene, a known carcinogen (Caron-Beaudoin et al., 2018), multiple trace toxicants (Claustre et al., 2023), and particulate air pollutants (Caron-Beaudoin et al., 2023) among pregnant women living close to UOGD activity.

Our review had several limitations. First, we only included health studies where the likely pathway was related to chemical exposure. Numerous studies looking at the effects of UOGD activity on other health outcomes (e.g., traffic accidents, sexually transmitted infections, mental health) were not included. Similarly, our review did not include studies focused on the social or economic changes related to UOGD and their impacts on health. Second, due to the heterogeneity of outcomes and exposure measurements, we did not conduct a systematic review or meta-analysis or systematically apply a formal bias assessment tool. Third, due to restricted resources, this review was limited to English- and French-language publications. Given that the top UOGD-producing countries after the USA are Russia, Iran, Qatar, and China, with Canada in sixth place (Statista, 2014), we may have missed studies published in other languages. Another limitation is possible publication bias given the potential for our review to amplify reporting and publication of positive versus negative findings. We have attempted to mitigate this by characterizing both harmful and protective effects and limiting these to reported effect estimates that reach statistical significance.

## Conclusion

There is a growing body of research, across multiple jurisdictions, reporting adverse effects of unconventional oil and gas development exposure on human health, with an accumulating weight of evidence particularly in relation to birth outcomes and asthma. There is some evidence of disproportionately greater impacts in racialized populations with relatively little research focused on the differential exposure levels and effect modification by systemically disadvantaged populations. The absence of Canadian published research on health effects of UOGD is notable given the geographic relationship between UOGD and Indigenous communities, the considerable time over which UOGD has taken place, and a policy of continued expansion of this activity in several provinces. There is a pressing need for future research focused on the following: prospective and community-based studies; a focus on Indigenous, racialized, rural, and disproportionately disadvantaged populations; improved exposure assessment including measurement of phase-specific UOGD, flaring, abandoned wells, and non-UOGD exposures; impacts of wildfires and NORMS; and characterization of both linear and non-linear nonmonotonic dose–response effects.

### Supplementary information

Below is the link to the electronic supplementary material.

Supplementary file1 (DOCX 257 KB)

## Data Availability

N/A.

## Code availability

N/A.
