# Correction: The human health effects of unconventional oil and gas development (UOGD): A scoping review of epidemiologic studies

**DOI:** 10.17269/s41997-024-00913-6

**Published:** 2024-06-21

**Authors:** Amira M. Aker, Michael Friesen, Lisa A. Ronald, Mary M. Doyle-Waters, Tim K. Takaro, Willow Thickson, Karen Levin, Ulrike Meyer, Elyse Caron-Beaudoin, Margaret J. McGregor

**Affiliations:** 1 Université Laval, CHU de Quebec – Université Laval, Québec, QC Canada; 2 Faculty of Health Sciences, Simon Fraser University, Burnaby, BC Canada; 3 Centre for Clinical Epidemiology and Evaluation, Vancouver Coastal Health Research Institute, Vancouver, BC Canada; 4 Department of Family Practice, Faculty of Medicine, University of British Columbia, Vancouver, Canada; 5 Emerald Environmental Consulting, Kent, OH USA; 6 Department of Health and Society and Department of Physical and Environmental Sciences, University of Toronto Scarborough, Toronto, ON Canada; 7 Dalla Lana School of Public Health, University of Toronto, Toronto, ON Canada

**Correction: Canadian Journal of Public Health (2024) 115:446-467**

10.17269/s41997-024-00860-2

This article was updated to correct Tim J. Takaro to Tim K. Takaro.

