# Supplementary material for: The human health effects of unconventional oil and gas development (UOGD): A scoping review of epidemiologic studies
Source: Can J Public Health. 2024 Mar 8;115(3):446–67. doi: 10.17269/s41997-024-00860-2 (PMC11133301; doi:10.17269/s41997-024-00860-2)
Supplement: Supplementary file 1 — Supplementary file1 (DOCX 257 KB) [file 41997_2024_860_MOESM1_ESM.docx]

**Electronic Supplementary Material for “The human health effects of unconventional oil and gas development (UOGD): A scoping review of epidemiologic studies”**

Contents

[Online Resource 1: Search terms for scoping literature review 3](#_Toc142923068)

[Online Resource 2: Exposure metrics (and data sources) for studies included in sCOPING review 7](#_Toc142923069)

[Online Resource 3: Characteristics and Results of included studies 12](#_Toc142923070)

[Online Resource 4: Further synthesis of health outcomes 43](#_Toc142923071)

# **Online Resource 1:** Search terms for scoping literature review

## Fracking Epi M8 Search Concepts and Limits


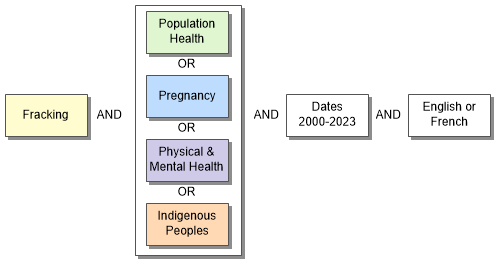


## MEDLINE Search – Fracking Epi M8 Update 2

**Database:** MEDLINE (OVID)

**Date:** Jan. 10, 2023

Database: Ovid MEDLINE(R) and Epub Ahead of Print, In-Process, In-Data-Review & Other Non-Indexed Citations, Daily and Versions <1946 to January 09, 2023>

Search Strategy:

----------------------------------------------------------------

1 Hydraulic Fracking/ (382)

2 (frack or fracking).mp. (584)

3 hydraulic fracturing.mp. (921)

4 hydrofracturing?.mp. (18)

5 hydrofracking.mp. (7)

6 fraccing.mp. (1)

7 UNGD.mp. (25)

8 unconventional natural gas.mp. (132)

9 gas well?.mp. (460)

10 (shale adj5 drill$).mp. (100)

11 (coal seam adj5 gas).mp. (97)

12 "Oil and Gas Industry"/ (644)

13 Natural Gas/ (1264)

14 (shale adj5 gas).mp. (902)

15 (unconventional adj5 (gas or oil or drilling)).mp. (612)

16 natural gas.ti,ab,kf. (3917)

17 "Oil and Gas Fields"/ (1658)

18 or/12-17 (6892)

19 (develop$ or drill$ or produc$ or extract$ or industry or explor$).mp. (9937291)

20 "Extraction and Processing Industry"/ (1289)

21 impact$.mp. (1494498)

22 industry/ (32735)

23 Water Pollutants, Chemical/ (126143)

24 Volatile Organic Compounds/ (12995)

25 or/19-24 (10846724)

26 18 and 25 (5386)

27 or/1-11,26 [Fracking broad] (6162)

28 or/1-11 [Fracking focused] (1688)

29 epidemiologic studies/ (9231)

30 health/ or adolescent health/ or women's health/ or men's health/ or mental health/ or occupational health/ (148733)

31 population health/ or rural health/ or suburban health/ or urban health/ (40605)

32 Public Health/ (93359)

33 Epidemiology/ (12569)

34 ep.fs. [Epidemiology] (2052458)

35 Environmental Health/ (14779)

36 Health Status/ (89339)

37 (health adj3 (impact? or effect? or risk? outcome?)).mp. (124206)

38 public health.ti,ab,kf. (353302)

39 (health adj3 (fetus or infant? or neonatal or child? or adolescen$ or maternal? adult?)).mp. (94008)

40 Risk Factors/ (941217)

41 ae.fs. [Adverse Effects] (1957562)

42 (adverse adj4 (health or outcome? or effect? or reaction?)).ti,ab,kf. (365842)

43 carcinogens/ or carcinogens, environmental/ (49929)

44 Residence Characteristics/ (37824)

45 incidence/ (297559)

46 Risk Assessment/ (303194)

47 Health Policy/ (71625)

48 Health Status Indicators/ (24074)

49 Health Equity/ (3225)

50 emergency service, hospital/ (84977)

51 emergency department?.mp. (120285)

52 hospitalization/ or patient admission/ (155363)

53 hospitali#ation.ti,ab,kf. (180875)

54 (hospital adj4 (admission? or admit$ or visit?)).mp. (129943)

55 (emergency adj4 (admission? or admit$ or visit?)).mp. (39036)

56 or/29-55 [Population Health] (5481137)

57 27 and 56 (1040)

58 health.mp. and sn.fs. [Statistics & Numerical Data] (488161)

59 28 and 58 (51)

60 57 or 59 [Fracking & Population Health] (1044)

61 exp Pregnancy/ (989377)

62 Pregnancy Outcome/ (56878)

63 Maternal Exposure/ (10908)

64 Premature Birth/ (19932)

65 Placenta/ (59303)

66 fetal hypoxia/ (3205)

67 infertility/ or infertility, male/ (40234)

68 exp reproduction/ (1231665)

69 "embryonic and fetal development"/ (22635)

70 exp Pregnancy Complications/ (468043)

71 Prenatal Exposure Delayed Effects/ (33526)

72 pregnan$.mp. (1112235)

73 exp embryonic structures/ (460036)

74 exp "congenital, hereditary, and neonatal diseases and abnormalities"/ (1346845)

75 exp Congenital Abnormalities/ (645166)

76 Neurodevelopmental Disorders/ (4267)

77 Birth Weight/ (43424)

78 Premature Birth/ (19932)

79 infant, low birth weight/ or infant, small for gestational age/ or infant, very low birth weight/ or infant, extremely low birth weight/ or infant, premature/ or infant, extremely premature/ (90335)

80 Neurodevelopmental Disorders/ (4267)

81 Learning Disabilities/ (14589)

82 birth.ti,ab,kf. (343728)

83 child health/ or infant health/ or reproductive health/ or maternal health/ (12154)

84 fetal mortality/ or infant mortality/ or perinatal mortality/ (31930)

85 gestational age/ (88329)

86 ((foetal or fetal or fetus) adj3 develop$).mp. (53727)

87 or/61-86 [Pregnancy & early childhood] (2987875)

88 27 and 87 [Fracking & Pregnancy & early childhood] (153)

89 exp Mental Disorders/ (1406182)

90 Occupational Stress/ (3311)

91 occupational exposure/ (59945)

92 exp "diseases (non mesh)"/ (16699720)

93 chronic disease/ (278813)

94 Neoplasm?.ti,ab,kf. (305559)

95 cancer$.mp. (2201013)

96 (Respiratory adj4 (disorder? or disease? or infection? or symptom? or health or exacerbation?)).ti,ab,kf. (152632)

97 asthma?.ti,ab,kf. (167527)

98 Inhalation Exposure/ (10285)

99 or/89-98 [Physical Health] (17764353)

100 27 and 99 [Fracking & Physical Health] (550)

101 indigenous peoples/ or alaska natives/ or inuits/ (5886)

102 american native continental ancestry group/ or indians, central american/ or indians, north american/ (15969)

103 oceanic ancestry group/ (12042)

104 maori$.mp. (4189)

105 first peoples.mp. (614)

106 indigenous.mp. (43016)

107 aboriginal.mp. (10029)

108 first nation$.mp. (6082)

109 (native adj3 people$).mp. (919)

110 (native adj3 (american$ or people$)).mp. (9776)

111 Metis.mp. (479)

112 indigenous canadians/ (103)

113 inuits/ (4029)

114 inuit$.ti,ab,kw,kf. (2086)

115 "american indians or alaska natives"/ or alaskan natives/ (1234)

116 indians, south american/ (3713)

117 ((American or Canadian) adj3 (Indian$ or Native$)).ti,ab,kw,kf. (14394)

118 or/101-117 [First Nations] (88336)

119 27 and 118 [Fracking & First Nations] (100)

120 or/60,88,100,119 [Search Results-1] (1288)

121 animals/ not (animals/ and humans/) (5046442)

122 120 not 121 (1135)

123 human health.ti,ab,kf. (71635)

124 27 and 123 [Search Results-2] (208)

125 health.ti,ab,kf. (2467243)

126 28 and 125 [Search Results-3] (342)

127 or/122,124,126 [All Search Results] (1271)

128 remove duplicates from 127 (1270)

129 limit 128 to yr="2021 -Current" (207)

130 limit 129 to (English or French) (205)

131 comment/ or editorial/ or letter/ or news/ (2321589)

132 130 not 131 (202)

133 "review"/ (3099151)

134 132 and 133 (18)

135 limit 132 to (systematic reviews pre 2019 or systematic reviews) (6)

136 134 or 135 [Reviews] (19)

137 132 not 136 [Remaining] (183)

# **Online Resource 2:** Exposure metrics (and data sources) for studies included in scoping review

|  | **Author, publication year** | **Main exposure metric(s)** | **Metric description**  **Exposure/outcome timing & time period** | **Data source** |
| --- | --- | --- | --- | --- |
|  | ***Cohort studies*** | | | |
| 1 | Apergis et al., 2019 | Well count within 4 geographic buffer zones from zip code of mother’s residence | Stratified into categories of 1, 5, 10 and 20 km measured as a continuous variable for number of UOGD wells within 1 km from the mother’s residence, or 1–5 km, or 5–10 km, and 10–20 km.  Exposure and outcome measured simultaneously. | The Oil and Gas Division of The Oklahoma Corporation Commission |
| 2 | Cairncross et al., 2022 | UOGD well density, well count, distance to nearest well | Buffer of 10 km.  Considered exposed if postal delivery point located within 10 km from at least 1 active well that was hydraulically fractured during preconception (1 year prior to conception date) and/or pregnancy (conception date to delivery date), and/or for each trimester. | Alberta Energy Regulator (AER)  GeoSCOUT  Developing Mathematical Thinking Institute |
| 3 | Caron-Beaudoin et al., 2021 | Inverse distance weighted (IDW) from 6-digit postal code of maternal residence at time of delivery | Three buffers employed: 2.5, 5 and 10 km.  Only wells that had a spud date earlier than the delivery date were included. Exposure and outcome measured simultaneously. | British Columbia Oil and Gas Commission |
| 4 | Casey et al., 2016 | Aggregate metric incorporating 4 phase-specific inverse distance weighted squared activity metrics (ID^2^W) of wells | Activity metric included latitude and longitude of each well; dates of well spudding, perforation, stimulation, and production; total well depth; volume of natural gas produced; and the number of producing days annually.  Exposure measured during the pregnancy. | PADEP  SkyTruth  Pennsylvania Department of Conservation and Natural Resources |
| 5 | Currie et al., 2017 | Presence of one or more active wells in proximity to maternal residence | A birth was determined to be ‘exposed’ if the mother lived within 0-1, 1-2, and 2-3 km vs 15 km and all of Pennsylvania (non-exposed).  Exposure measurement at or after estimated date of conception. | PADEP |
| 6 | Cushing et al., 2020 | Number of nightly flare events, total flared area from all flares, IDW sum of flares, number of oil and gas wells within 5 km | For flaring activity three metrics were used: nightly flare events within 5 km of maternal residence (split into low/high), total flared area from all flares occurring within 5 km of maternal residence (split into low/high) and ID^2^W of flares within 5 km of maternal residence (split into low/high).  Researchers also calculated trimester-specific estimates using the main estimate of number of flares within 5 km.  For O&G wells the exposure metric was number of permitted wells within 5 km of maternal residence, split into quartiles.  Exposure measurement averaged over the pregnancy and trimester specific. | Enverus  Visible Infrared Imaging Radiometer Suite |
| 7 | Hill, 2018 | Distance of maternal residence to nearest O&G well, number of active wells | Within 2.5 km of maternal address, births before and after drilling.  Active well within 2.5 km of maternal address and outcome measured simultaneously. | PADEP |
| 8 | Hill and Ma, 2022 | Shale gas well count w/in 10 km of its water source (or residence), drilled within the gestation period of an infant living within the radius. | Residence within 10 km radius of ground water-based community water system with any active drilling over gestational period.  Residing <1km vs 5-10km by # wells w/in ground water-based community water system.  UNGD chemicals present in ground water.  Exposure time varying by day and outcomes measured simultaneously (month of delivery). | PADEP  PADOH |
| 9 | Janitz et al., 2019 | IDW of wells of maternal residence | Three buffers employed: 2.5, 5 and 10 miles.  Exposure and outcomes measured simultaneously (month of delivery). | The Oklahoma Corporation Commission |
| 10 | Li et al., 2022 | IDW of wells linked to zip code | Proximity-based exposure metric IDW w/ 5 km buffer & down-wind exposure metric calculated using the IDW of all wells that fall within the downwind circular quadrant.  Monthly ZIP-code-level exposure metrics aggregated to annual ZIP-code-level exposures.  Zip codes divided into 4 exposure quartile and 2 downwind categories (8 categories).  Zip codes linked to ZIP code of individual Medicare beneficiary residing in a given UOGD area. | Enverus  Monthly prevailing wind direction data |
| 11 | McKenzie et al., 2014 | IDW of unconventional wells of maternal residence | Buffer of 16.2 miles used.  Unconventional exposure assumed based on time period examined. | COGIS |
| 12 | Stacy et al., 2015 | IDW of unconventional wells of maternal residence | Buffer of 10 miles used divided into exposure quartiles.  Exposure based on year of birth | PADEP |
| 13 | Tran et al., 2020 | Inactive well count; production volume from active wells | 1 km buffer used for analyses.  Inactive well count based on categories of: low (1), med (2-5) and high (6+).  Barrels of oil equivalent (BOE) per day metric employed to measure fracking production. BOE derived by combining monthly barrels of oil and barrels of oil equivalent of natural gas.  Exposure categories for low (no BOE/day) medium 1-100 BOE/day and high GT 100BOE/day.  Exposure majority conventional production (estimated 80%).  Exposure measured for entire pregnancy based on estimated date of conception and trimester specific. | CA DOGR |
| 14 | Tran et al., 2021 | Women w/ at least one well stimulated (hydraulically fractured) within 1 km of their residential address at any point during pregnancy | Buffer of 1 km.  Reference group was women w/ no stimulated wells within 1 km, but at least one oil/gas wells w/in 10 km.  Exposure measured by estimating period of gestation based on date of birth from birth certificate data. | CA DOGR  California Council on Science and Technology’s (CCST) well stimulation report |
| 15 | Whitworth et al., 2017 | IDW of unconventional gas wells | Three separate buffers employed: 0.5, 2, and 10 miles.  Women with no wells within 10 miles of their address used as a reference group, categorized into tertiles.  Exposure based on mother’s address of residence linked to matched trimester based on infant date of birth. | Enverus |
| 16, 17 | Willis et al., 2022, 2021 | Distance to nearest active oil or gas wells | Looked at 4 distance zones (0-1, >1-2 or >2-3 km) and 3-10 km from an active or future drilling site w/ latter being one geographic reference group.  The other temporal control group was women who gave birth and residing near drilling sites before and after drilling began.  Effect of exposure to oil, gas, conventional and unconventional analysed separately for 2022.  Exposure and outcomes measured simultaneously (month of delivery). | Enverus |
| 18 | Willis et al., 2023 | Distance to nearest active unconventional and conventional oil and gas wells and IDW of active wells based on av monthly produced oil, gas and water | 5 km buffer: Tertiles of inverse distance-squared weighting w/in 5 km for drilling site count, gas production, oil production, and produced water compared to maternal residence with future (but no active) drilling sites within 5 km.  10km buffer w/1km bins from 0-10 km, w/ reference set to 9-10 km group. | Enverus |
|  | ***Case-control studies*** | | | |
| 19 | Clark et al., 2022 | ID^2^W of active wells, & water pathway-specific metric linked to birth address | Buffers of 2, 5, and 10 km.  Unconventional O&G wells that were active and water pathway flow-direction metric based on land-surface topography based on the assumption that Unconventional O&G wells located upgradient of a residence contribute more to exposure than downgradient wells and presuming that consumption or contact with groundwater from domestic wells is a major exposure source.  2 exposure windows - 3 months prior to conception to 1 year prior to diagnosis based on date of diagnosis, (primary window) and 3 months prior to conception to birth based on date of birth (perinatal window). | PADEP  Land surface topography maps  Pennsylvania cancer registry  Pennsylvania birth records |
| 20 | Elser et al., 2021 | Average annual block group-level P<2.5 & N0_2_ concentrations, IDW from 60 methane super emitters & from active oil and gas wells | Buffer of 10 km.  IDW of emitted methane and active well w/in 10 km of residence.  Binary measure for any methane emission & active well w/in 10 km (yes/no).  Exposure and outcomes measured simultaneously (index date of diagnosis). | CARB  CA DOGR |
| 21 | Koehler et al., 2018 | Three distance-weighted, phase-specific approaches that incorporated wells, compressors, impoundments and flaring events | Three methods were employed: (1) the categorical distance to the nearest drilled well (DNDW); (2) the inverse distance metric based on the drilling phase (IDW1); and (3) the inverse distance-squared metric (IDW2) incorporating the four phases of well development and compressor engines (IDW24C).  Exposure measured one day prior to outcome. | PADEP |
| 22 | McAlexander et al., 2020 | IDW measure, phase specific | Adapted from Koehler et al. (2018).  Exposure measured over 30 days with a 1-day lag before hospitalization. | PADEP |
| 23 | McKenzie et al., 2017 | IDW for active wells | Buffer of 10 miles used. In sensitivity analyses, 2- and 5-mile buffers were employed.  Exposure measurement in year of outcome. | COGIS |
| 24 | McKenzie et al., 2019a | IDW measure for active wells; O&G facilities other than wells; intensity adjusted air pollution not associated with oil and gas | Buffer of 10 miles used. In sensitivity analysis, 2-mile buffer was employed.  Exposure measured over 3 months prior to conception to the second gestational month. | COGIS |
| 25 | Rasmussen et al., 2016 | IDW, phase specific | Index accounts for IDW, well depth, volume of gas produced.  Divided into quartiles w/ very low as the reference group.  Exposure measured one day prior to outcome. | PADEP  SkyTruth  Pennsylvania Department of Conservation and Natural Resources |
| 26 | Tang et al., 2021 | Well density | UNGD well densities were defined as the number of yearly-active wells in a geographic area, using areas of three different buffers: within 1, 3, and 7.5 km of maternal address at year of birth.  Exposure and outcomes measured simultaneously. | Railroad Commission of Texas (TXRRC) |
| 27 | Walker Whitworth and Symanski, 2018 | IDW, phase specific | Drilling phase metric incorporated number and distance of wells within 0.5 miles of maternal residence in an IDW fashion.  Production metric included the amount of gas production from wells within 0.5 miles and number and distance of wells within the 0.5-mile buffer in an IDW fashion.  Activity index divided into quartiles.  Mothers without an active well within 0.5 miles of their home address used as reference group.  Exposure measured across pregnancy period and was trimester specific. | Enverus |
|  | ***Cross-sectional/survey studies*** | | | |
| 28 | Blinn et al., 2020 | IDW metric and cumulative well density (# of wells/in 5 km radius around home/ area of the radius)  Annual emissions concentration (AEC) | 5 km buffers around residential addresses used for both metrics as continuous variables.  IDW and cumulative well density and outcome measured simultaneously, AEC modelled for the year corresponding to the respondents self-reported health assessment. | PADEP |
| 29 | Brown et al., 2019 | Number of sources per direction & median ambient air level of CO, NOx, PM 2.5, VOCs, and formaldehyde | Applied a “box” air pollution dispersion screening model to estimate the median ambient air level of CO, NOx, PM 2.5, VOCs, and formaldehyde at  the residence during the year health symptoms were reported. Used the model to calculate ambient air levels at seven unique distances ranging from 0.1 km (i.e., “fence line”) to 10 km from hypothetical source. Models included hourly cloud cover, wind speed, time of day, and wind direction  Sources and median emissions were categorized as north, south, east, or west of the residence to account for the effect of wind direction on dispersion and representing the number of sources in each quadrant within 2 km of the residence. Generated four additional variables (“north emissions”, “south emissions”, “east emissions”, and “west emissions”) which represented the median of ambient air levels of emissions from wells, processing plants, and compressor stations in the quadrant. | PADEP - data collected at the Allegheny County Airport in West Mifflin, PA |
| 30 | Elliott et al., 2018 | Distance of maternal residence to nearest well; IDW, presence of VOCs, DBPs, GROs and DROs in drinking water samples | Three metrics were calculated: distance to nearest active well, IDW/ID^2^W within a 5 km buffer of residence, and IDW for 1 and 2 km buffers and drilling and production phase specific activity metrics (for sensitivity analyses).  Presence of VOCs, DBPs, GROs and DROs measured in ground water.  Exposure and outcome measured simultaneously. | Ohio Department of Natural Resources  Direct measurement of drinking water by researchers |
| 31 | Johnston et al., 2021 | Distance to nearest well | Constructed a 4-level categorical exposure variable: living upwind and more than 200 m from O&G development wells (reference); living upwind and within 200 m; living downwind and more than 200 m; and living downwind and within 200 m (Model 3); models with distance modeled as a continuous variable using a lognormal transformation (Model 4).  One O&G development well site housed 21 wells which were idle during the study period. The second O&G development well site had 28 wells at the time of the study and was actively producing oil during the entire study period. | CalGEM |
| 32 | Mayer et al., 2021 | Communities with no, some and high UNGD activity | Community with no UNGO, a lot of UNGO, and with permitted wells and little active drilling (i.e., some UNGD). | American Community Survey  Publicly available information on respective policies of the three communities with respect to allowing UNGD |
| 33 | McKenzie et al., 2019b | IDW | Buffer of 16 km used; divided into low, medium, and high tertiles.  Metric incorporates all drilling stages, as well as well production and depth.  Exposure and outcomes measured simultaneously. | COGIS |
| 34 | Rabinowitz et al., 2015 | Distance of residence to nearest O&G well | Divided into tertiles of <1, 1-2, and >2 km.  Exposure and outcomes measured simultaneously. | PASDA |
| 35 | Steinzor et al., 2013 | Distance from residence to nearest facility | Participants self-reported the distance from their residence to three types of facilities (compressor and pipeline stations, gas-producing wells, and impoundment or waste pits). Testing in subset of households for VOCs/benzene, toluene, ethylbenzene and xylene, alpha/beta radiation, radon, and radium in water and air.  Exposure and outcomes measured simultaneously. | Self-report |
| 36 | Tustin et al., 2017 | IDW incorporating phase | Adapted from Casey (2016).  Exposure measured 90 days prior to the date of survey completion. | PADEP  SkyTruth  Pennsylvania Department of Conservation and Natural Resources |
|  | ***Ecologic studies*** | | | |
| 37 | Apergis et al., 2021 | Number of drilled oil and natural gas wells using fracking technologies per county | Number of fracked wells per county before and after fracking boom (2006).  Outcome and exposure measured concurrently (check). | Oil and Gas Division of the Oklahoma Corporation Commission |
| 38 | Busby and Mangano, 2017 | Number of wells within county, contrasting the 10 most heavily fracked counties with the rest of Pennsylvania | Measurement split into two time periods: pre (2003-2006) and post (2007-2010) fracking boom in Pennsylvania.  Number of violations and number of water wells per county also included.  Exposure and outcome measured simultaneously. | PADEP  Pennsylvania Department of Conservation and Natural Resources |
| 39 | Bushong et al., 2022 | Annual well density of active unconventional O&G wells by county | Active UNGD wells from the years 2001–2014 (7489 wells) were sorted by date and matched to their county to determine the number of wells spudded each year for every county.  Calculated the well density for active UNGD wells spudded during a calendar year in each county using the area of each county provided by the US Census Bureau through TIGERweb.  Exposure and outcome measured simultaneously. | PADEP  US Census Bureau (TIGERweb)  PASDA |
| 40 | Denham et al., 2019 | Number of new wells drilled; total number of wells; well density | Three exposure metrics were employed: a contemporaneous well measure (unconventional gas wells drilled in a year), cumulative well count (total number of unconventional gas wells drilled up to the end of a given year) and cumulative well density (at a county level).  Exposure and outcomes measured simultaneously. | The Carnegie Museum of Natural History's Pennsylvania Unconventional Gas Wells Geodatabase |
| 41 | Denham et al., 2021 | Number of new wells drilled; total number of wells; well density | Three county-level exposure metrics were employed: drilled wells in a quarter-year (contemporaneous drilled wells) and two cumulative well measures: well count (total number of wells drilled up to the end of the quarter-year) and well density (cumulative well count divided by county land area in square miles).  Exposure and outcome measured simultaneously. | The Carnegie Museum of Natural History’s Pennsylvania Unconventional Gas Wells Database |
| 42 | Erickson et al., 2022 | Hydraulic fracture well density and well production | Well density calculated by number of wells divided by the total surface area of the country annual well production log transformed | Colorado Department of Natural Resources, Oil & Gas Conservation Commission |
| 43 | Finkel, 2016 | Number of producing wells per county | Residence in 2 counties with high number of producing wells counties to 2 counties with medium and 2 with low number of producing wells, before and after drilling activity.  Exposure and outcome measured simultaneously. | PADEP |
| 44 | Fryzek et al., 2013 | Number of drilled wells within county | Researchers used spud data as a marker of before/after drilling commenced. Counties with no active wells used as control.  Exposure and outcome measured simultaneously. | PADEP |
| 45 | Hu et al., 2022 | Modelled exposure fracking activity risk based upon occurrence of pollutant leakage due to fracking (fracking density/sq km x annual rate of occurrence/fracking/sq km) | Concentration monitoring for Butadiene, Benzene, Formaldehyde, Acetaldehyde  Annual Loss Expectancy (ALE) = Annual Rate of Occurrence (ARO) of pollutant leakage due to fracking X Single Loss (SLE  ARO = fracking density per sq km (FD) X annual rate of occurrence per fracking sqm k (AROF)  SLE = population with negative health impacts expected from the occurrence of fracking accidents = population density per sq km X fracking exposure factor (EF)  Fracking ALE represents the average loss per year of environmental pollution cause by fracking on public health | Hazardous Air Pollutants (HAPS); EPA  CDC: Centres for Disease Control and Prevention  USCB; US Census Bureau; FF United States Frac Focus  United States Environmental Protection Agency |
| 46 | Jemielita et al., 2015 | Well counts; well density | Measurement conducted at zip code level.  Exposure and outcome measured simultaneously. | PADEP |
| 47 | Ma et al., 2016 | Presence of active well within zip code based on earliest date of unconventional drilling commencement and unconventional well density | Exposure measured at estimated time of conception and linked to maternal address at birth. | PADEP |
| 48 | Makati et al., 2022 | West Virginia Natural Gas Withdrawls (MMcf) per year | Before 2010 vs after 2010 period production volumes | U.S. Energy Information Administration |
|  |  |  |  |  |
| 49 | Peng et al., 2018 | Presence of active well within county  Volume of gas produced within county | Measurement at the country level.  Well current year, well previous year. | PADEP |
| 50 | Schuele et al., 2022 | IDW, ID^2^W of unconventional and conventional well production linked to maternal county population-adjusted centroid aggregated to county level | No buffer exposure measures evaluated as continuous.  Production exposure averaged over nine months period prior to birth. | Enverus/ DrillingInfo |
| 51 | Willis et al., 2018 | Newly drilled wells; cumulative wells ever drilled, tertiles of wells ever drilled and reported UNGD air pollutants | Three metrics were used at zip code level: a binary contemporaneous variable for a newly spudded (initially drilled) well within a specific quarter, a binary cumulative variable for ever-spudded wells, tertiles of cumulative count of the wells ever drilled, and reported air emissions by site.  Exposure and outcome were measured simultaneously. | PADEP  Pennsylvania Unconventional Natural Gas  Emission Inventory  DrillingInfo |
| 52 | Willis et al., 2020 | Cumulative well density stratified by conventional and unconventional, gas production, flaring volumes | Zip code level total, conventional and unconventional cumulative count of natural gas/ km sq, flaring, and production volumes by zip code and quarter, divided into tertiles, 2000 – 2010.  Exposure and outcome were measured simultaneously. | Enverus  DrillingInfo  Railroad Commission of Texas (TXRRC). |

AEC = annual emissions concentration; BOE = barrels of oil equivalent; CA DOGR = California Division of Oil, Gas and Geothermal Resources; CalGEM = California Geologic Energy Management; CARB = California Air Resources Board; COGIS = Colorado Oil and Gas Information System; DBPs = disinfection by-products; DNDW = distance to the nearest drilled well; DRO = diesel-range organics; GROs = gasoline-range organics; IDW = inverse distance weighted metric of wells unless otherwise stated; ID^2^W = inverse distance weighted squared activity metric; km = kilometres; NO_2_ = nitrogen dioxide; O&G = oil and gas; PADEP = Pennsylvania Department of Environmental Protection; PADOH = Pennsylvania Department of Health; PASDA = Pennsylvania Spatial Data Access; UNGD = unconventional natural gas development; UOGD = unconventional oil and gas development; US = United States; VOCs = volatile organic compounds; w/ = with.

# **Online Resource 3: Characteristics and results of included studies**

|  | **Author’s Name, Year** | **Study Population**  **Location, Size (n),**  **Years of data collection** | **Outcome(s),**  **Outcome data source(s)** | **Analytic approach** | **Covariates,**  **Data source(s)** | **Main effect estimates for the association of UOGD exposure measures with outcome** | **Authors’ reported results & conclusions** | |
| --- | --- | --- | --- | --- | --- | --- | --- | --- |
|  | ***Cohort Studies*** | | | | | | | |
| 1 | Apergis et al., 2019 | Newborns  US (Oklahoma)  556,794  1996 - 2005  2006 - 2017 | Perinatal outcomes –  TBW, LBW, composite infant health index by well count  Birth records from Oklahoma Health Department | Regression analysis with exposure metric (well count): a continuous variable representing number of UOGD wells within 4 buffers zones for two time periods (before and after increased UOGD activity). | Maternal age, education, ethnicity/mother’s race (white African-American, Asian, Hispanic, other)  Birth records from Oklahoma Health Department | TBW β Coefficient:  -0.084 for 0-1 km, *p<*0.01  -0.078 for 1-5 km, *p<*0.05  -0.048 for 5-10 km  -0.025 for 10-20 km  LBW β Coefficient:  0.056 for 0-1 km, *p<*0.01  0.046 for 1-5 km, *p<*0.01  0.039 for 5-10 km, *p<*0.10  0.014 for 10-20 km  Infant health index β Coefficient:  -0.067 for 0-1 km, *p<*0.01  -0.062 for 1-5 km , *p<*0.01  -0.053 for 5-10 km, *p<*0.05  -0.022 for 10-20 km, *p<*0.10  No significant effect of distance on outcomes for yrs where there was less fracking and for conventional drilling exposure. | Decreasing TBW (up to 5 km exposure), higher risk of LBW, and poorer infant health index associated with maternal proximity to higher number of fracking wells (all buffer zones).  There is a unidirectional relationship between fracking activities and three alternative indexes of infants’ health at birth, as well as a significant impact of fracking on infants’ health indicators. | |
| 2 | Cairncross et al., 2022 | Maternal-infant pairs living in rural Alberta  Canada (Alberta)  26,193  2013 - 2018 | Perinatal outcomes - spontaneous PTB, indicated PTB, SGA, major congenital anomalies, severe infant morbidity and/or mortality by distance to nearest well and well density  Alberta Health Services – Discharge Abstract Database, National Ambulatory Care Reporting System, physician claims data | Poisson regression with sandwich variance for binary outcomes, generalized estimating equations (GEE) for more than one pregnancy by same mother, examination of multiple exposure windows separately and together, by trimester, and by pre and post conception.  Association of outcomes with three exposure measures 1) within or outside 10 km (yes/no), 2) distance to nearest well, 3) well 3density within 10 km divided into quintiles. | Maternal age at delivery, multiple births, infant sex, obstetric comorbidities (composite measure), postal code level Pampalon index for maternal and social deprivation,  Discharge Abstract Database, physician billing data, National Ambulatory Care Reporting System, postal code of maternal address | RR (95% CI) exposure to a hydraulic fracturing site within 10 km in preconception or pregnancy vs unexposed (yes/no)  SGA: 1.12 (1.03, 1.23)  Major congenital anomaly: 1.31 (1.01, 1.69)  RR (95% CI) for pregnant women living within 10 km of 1 or more wells during preconception and pregnancy by increasing exposure level: ref = 1-24 wells  Spontaneous PTB:  25-49 wells: 0.88 (0.69, 1.13)  50-74 wells: 0.98 (0.72, 1.33)  75-99 wells: 0.92 (0.59, 1.43)  >100 wells: 1.64: (1.04, 2.60)  SGA:  25-49 wells: 1.07 (0.98, 1.30)  50-74 wells: 1.07 (0.85, 1.35)  75-99 wells: 0.67 (0.45, 1.01)  >100 wells: 1.65 (1.10-2.48)  No significant effect for indicated PTB. | SGA and major congenital anomalies were significantly higher for individuals who lived within 10 km of at least 1 hydraulically fractured well after adjusting for maternal age at delivery, multiple births, infant sex, obstetric comorbidities, and area-level socioeconomic status.  Risk of spontaneous PTB and SGA were significantly increased in those with 100 or more wells within 10 km.  Results suggest that individuals who were exposed to hydraulic fracturing within pregnancy may be at higher risk of several adverse birth outcomes. These results may be relevant to health policy regarding legislation of UOGD in Canada and internationally. | |
| 3 | Caron-Beaudoin et al., 2021 | Maternal-infant pairs in Fort St. John, British Columbia  Canada (British Columbia)  5,018  2006 - 2016 | Perinatal outcomes –  PTB, TBW, SGA, head circumference.  Northern Health Authority of British Columbia perinatal data registry & prenatal registration questionnaire. | Linear and logistic regression for IDW variable on outcomes for 3 buffer zones by IDW quartile., trend tested | Maternal age, parity, infant’s biological sex, and smoking status.  Northern Health Authority of British Columbia perinatal data. registry & prenatal registration questionnaire | Average difference (95% CI) of TBW (in grams (g)):  5 km buffer:  2^nd^ quartile: −40.87 (−78.01, −3.73)  3^rd^ quartile: −42.01 (−79.15, −4.87)  4^th^ quartile: −1.89 (−38.75, 34.98)  10 km buffer:  2^nd^ quartile: −29.22 (−66.28, 7.84)  3^rd^ quartile: −47.28 (−84.30, −10.25)  4^th^ quartile: −10.51 (−47.30, 26.27)  OR (95% CI) of PTB in 2.5 km buffer:  2^nd^ quartile: 1.60 (1.30, 2.43)  3^rd^ quartile: 1.34 (0.90, 2.08)  4^th^ quartile: 1.18 (0.76, 1.86)  No association of hydraulic fracturing exposure measure and head circumference, SGA | A negative association was found between postal code well density/proximity and birthweight for infants born to women in the 2nd quartile of the 10 km buffer (β (95% CI) −47.28 g (−84.30, −10.25)), and in the 2nd (β (95% CI) −40.87 g (−78.01, −3.73)) and 3rd (β (95% CI) −42.01 g (−79.15, −4.87)) quartiles of the 5 km buffer.  Increased odds of PTB were observed among women in the 2nd quartile of the 2.5 km buffer (OR (95% CI) 1.60 (1.30, 2.43)).  Results show inconsistent patterns of association between hydraulic fracturing, PTB and reduced birthweight, and effect estimates did not match expected dose-response relationships. | |
| 4 | Casey et al., 2016 | Newborns  US (Pennsylvania)  10,496  2005 - 2013 | Perinatal outcomes –  Apgar, PTB, SGA, TBW by IDW quartile.  Geisinger Health System electronic health system including labor and delivery notes and a separate labor and delivery database. | Multilevel linear and logistic regression models to examine associations between UODG activity index quartile and outcomes. | Sex, gestational age, season and year of birth, maternal age, race/ethnicity, Geisinger primary care provider status, smoking status during pregnancy, pre-pregnancy BMI, parity, antibiotic orders during pregnancy, and receipt of medical assistance.  Geisinger Health System electronic health system. | Adjusted TBW difference in g (95% CI) (ref = 1^st^ UNGD activity quartile) UNGD activity quartile:  2^nd^ quartile: −21 (−46, 5)  3^rd^ quartile: −9 (−35, 16)  4^th^ quartile: −31 (−57, 5)  This model lost significance once model adjusted for year of birth PTB:  2^nd^ quartile: 1.2 (0.9, 1.6)  3^rd^ quartile 1.3 (1.0, 1.7)  4^th^ quartile: 1.4 (1.0, 1.9)  There were no associations of UNGD with Apgar score, and SGA.  In post hoc analysis, UNGD associated with physician-recorded high-risk pregnancy identified from the problem list (4^th^ vs 1^st^ quartile) 1.3 (1.1, 1.7). | In adjusted models, there was an association between unconventional natural gas development activity and PTB that increased across quartiles, with a fourth quartile OR (95% CI) of 1.4 (1.0, 1.9).  There were no associations of activity with Apgar score, SGA, or TBW (after adjustment for year).  In a post-hoc analysis, there was an association with physician-recorded high-risk pregnancy identified from the problem list (fourth vs. first quartile, 1.3 (1.1, 1.7)).  Prenatal residential exposure to UNGD activity was associated with two pregnancy outcomes, adding to evidence that UNGD may impact health. | |
| 5 | Currie et al., 2017 | Newborns  US (Pennsylvania)  1,125,748  2004 - 2013 | Perinatal outcomes – LBW, TBW, composite infant health index comprised of a combination of birthweight, indicators for LBW, prematurity, congenital anomalies, and any other abnormal condition of the newborn by proximity to wells.  Birth certificate data. | Difference in difference analysis comparing outcomes of mothers living at different distances from active fracking sites both before and after drilling was initiated at each site, using county fixed effects. | Child’s sex, maternal race and ethnicity, mother's age, mother's education, marital status, child parity.  Birth certificate data. | β estimates for entire cohort, interaction effect of spudded well proximity X “after” time period:  LBW:  0-1 km: 0.016, *p<*0.05  1-2 km: 0.006+  2-3 km: 0.009, *p<*0.01  TBW (g):  0-1 km: −38.654, *p<*0.05  1-2 km: −3.534  2-3 km: −7.092  Infant health index:  0-1 km: −0.054, *p<*0.01  1-2 km: −0.020, *p<*0.05  2-3 km: −0.028, *p<*0.01 | There was a significant association of proximity to UOGD and LBW, decreased TBW and poorer infant health index. The largest impacts were seen within 1 km of fracking sites.  There is little evidence for health effects beyond 3 km, suggesting that health impacts are highly local. | |
| 6 | Cushing et al., 2020 | Newborns born to mothers living in highly fracked areas in rural Texas  US (Texas)  23,487  2012 - 2015 | Perinatal outcomes – PTB, SGA, gestational age (continuous variable), TBW by exposure to flaring and number of wells.  Birth records from the Texas Department of State Health Services. | Multivariate logistic and linear regression models used to estimate associations between exposure to a low or high number of nightly flare events and number of wells vs no exposure, while controlling for covariates.  Stratified analysis for Hispanic vs non-Hispanic white women | Maternal age, race/ethnicity, nativity, educational attainment, pre-pregnancy BMI, smoking, insurance based on primary source of expected payment, parity, high-risk pregnancy, sex of infant, adequacy of prenatal care, year and season of birth, rural vs small town. gestational age (weeks) for TBW model  Secondary analysis included number of wells within 5 km as additional covariate  Birth records from the Texas Department of State Health Services. | OR (95% CI) for proximity to low and high flaring within a 5 km buffer, (ref) = no flaring:  PTB:  Low flaring: 0.82 (0.61, 1.04)  High flaring: 1.50 (1.23, 1.83)  Hispanic women, adjusting for # wells:  Low flaring: 0.72 (0.52, 0.99)  High flaring (1.61 [1.25, 2.08]  Gestational age (days):  Low flaring: 0.1 (-1.9, 1.0)  High flaring: -1.9 (-0.9, -2.8)  Hispanic women, adjusting for # wells:  Low flaring: 0.6 (-0.7, 12.0)  High flaring: -2.2 (-3.4, -0.9)  OR (95% CI) for residential proximity to number of wells within a 5 km buffer, (ref)= no flaring:  PTB :  Low: 1.19 (1.06, 1.35)  Med: 1.35 (1.19, 1.53)  High: 1:31 (1.14, 1.49)  Gestational age (days):  Low: -1.0 (-1.5, -0.5)  Med: -1.8 (-2.3, -1.2)  High: -1.3 (-1.9, -0.8)  TBW (g):  Low: 0.4 (-15.7, 16.6)  Med: -18.6 (-35.5, -1.7)  High: -19.4 (-36.7, -2.0) | Exposure to a high number of nightly flare events was associated with 50% higher odds of PTB (OR (95% CI) 1.50 (1.23, 1.83)) and shorter gestation (mean difference (95% CI) −1:9 (−2:8, −0:9) days) compared with no exposure.  Flaring and fetal growth outcomes (TBW, SGA) were not significantly associated.  Effect estimates were slightly reduced after adjustment for the number of wells within 5 km. In stratified models these associations were present only among Hispanic women.  Women exposed to a high number of wells (fourth quartile, ≥27) vs. no wells within 5 km had a higher odds of PTB OR (95% CI) 1:31 (1.14, 1.49)), shorter gestation −1:3 (−1:9, −0:8) days, and lower average birthweight −19:4 (−36:7, −2:0) g.  The study suggests exposure to flaring from OGD is associated with an increased risk of PTB. | |
| 7 | Hill, 2018 | All births  US (Pennsylvania)  21,610  2003 - 2010 | Perinatal outcomes – Apgar, LBW, PTB, TGW, SGA, gestation period, infant health index by distance to nearest well and well density.  Pollution (CO, NO_x_, PM_2.5_, SO_x_, VOC) by #wells, and production at zip code level  Vital statistics natality and mortality data, Pennsylvania.  PADEP Air Emissions Inventory for Unconventional Natural Gas Operations 2011-2015 for pollution. | Difference in difference analysis looking at outcomes comparing outcomes of mothers living within 2.5 km of active shale gas well compared to mothers living within 2.5 km of permitted wells both before and after drilling was initiated (within 2.5 km*post-drilling).  Association of well number and 10 reported air pollutants also examined. | Race, education, age, marital status, WIC status, insurance type, previous risky pregnancy, whether the mother smoked during her pregnancy, month of birth, year of birth, and sex of the child.  Vital statistics natality and mortality data, Pennsylvania. | Adjusted β estimate among infants born to mothers living within 2.5 km radius of shale gas well compared to mothers living within 2.5 km of permitted wells:  LBW:  Residence proximal to any well within:  <2.0 km: 0.0127, *p<*0.05  < 2.5 km: 0.0136, *p<*0.05  <3.0 km: 0.0115, *p<*0.10  <3.5 km: 0.00912, *p<*0.05  Effect estimates for other outcomes of residence within <2.5 km of any well:  TBW: -49.58, *p<*0.01  PTB: 0.000354  Birth weight: -47.02, *p<*0.01  APGAR <8: 0.0251, *p<*0.05  Gestation: -0.0143  SGA: 0.0181, *p<*0.05  Congenital anomaly: -0.00193  Summary index: 0.0264, *p<*0.05  Effect estimates of well density on prematurity based on number of wells within:  <2.0 km: 0.00366, *p<*0.10  <2.5 km: 0.00257, *p<*0.05  <3.0 km: 0.00212, *p<*0.05  <3.5 km: 0.000889 | The introduction of drilling (living near any active well) increased LBW and decreased TBW on average among mothers living within 2.5 km of a well compared to mothers living within 2.5 km of a permitted well. Adverse effects were also detected using measures such as SGA and Apgar scores, while no effects on gestation periods were found.  In the intensive margin (well density), an additional well is associated with a 7 percent increase in LBW, a 5 gram reduction in TBW and a 3 percent increase in premature birth.  These results are robust to other measures of infant health, many changes in specification and falsification tests.  Findings suggest that shale gas development poses significant risks to human health.  Each additional well contributes an average of 0.5 tons of CO, 2 tons of NO_x_, 0.07 tons of PM_2.5_, 0.03 tons of SO_x_, and 0.17 tons of VOCs per year.  The top tertile (14–213 wells) of zip codes experience an average of 28 tons of CO, 90 tons of NO_x_, 2.6 tons of PM_2.5_, 1.8 tons of SO_x_, and 9 tons of VOCs per year. | |
| 8 | Hill and Ma, 2022 | Maternal-infant pairs living in residences within 10 km of any drilling.  US (Pennsylvania)  325,439  2003 - 2015 | TBW, gestation length, LBW, PTB by number of shale gas wells drilled at different distances from mother’s CWS during pregnancy.  Birth records from the Pennsylvania Department of Health. | Difference in difference analysis. | Individual: mother’s age, race, education, enrollment in Medicaid & WIC; smoking; previous live births; previous dead births, pre-gestational risks (including diabetes, poor outcome for a previous birth, a previous PTB; infertility risk; and had any risks during the current pregnancy (including gestational diabetes and vaginal bleeding). Also control for the gender of the infant and birth order fixed effects.  In utero exposure to the number of well bores drilled within 1 km of the maternal residence during gestation.  Environmental: average gestational temperature and precipitation near the maternal residence; changes in water quality of the mother’s water system that is not related to shale gas development; the number of permitted well bores during an infant’s gestation; ambient air quality at the Census block-group-by-year level; distance between maternal address and the closest Pennsylvania state-owned and maintained public road.  Month-by-year fixed effects and a fixed effect for each CWS.  Pennsylvania Department of Health records. | Adjusted β estimate among infants born to mothers living where wells drilled within 1 km radius of CWS compared to mothers living where wells drilled within 5 – 10 km of CWS source:  CWS fixed effects:  TBW: − 24.92, *p<*0.01  LBW: 0.00850, *p<*0.10  Gestational length: − 0.152, *p<*0.01  Premature: 0.0104, *p<*0.05  Mother fixed effects:  TBW: − 26.10, *p<*0.10  LBW: 0.0123. *p<*0.10  Gestational length: − 0.157, *p<*0.01  Premature: 0.0152, *p<*0.05  Log result shale gas development chemical detection in community water systems associated with drilled well bores within:  < 0.5 km: 0.0133, *p<*0.05  0.5 – 1 km: 0.00907, *p<*0.05  1-2 km: -0.000347  Log results shale gas development chemical detection in community water systems associated shale gas development uphill vs downhill of CWS:  <1 km: 0.0101, *p<*0.05 | Each drilled well decreases birth weight by 24.9 g (𝑝<0.01) when controlling for CWS fixed effects and by 26.1 g with the inclusion of mother fixed effects (𝑝<0.1).  Each gas well drilled within 1 km of an infant’s water source during pregnancy reduces gestation length by between 0.13 weeks (maternal fixed effects, 𝑝<0.05) to 0.15 weeks (CWS fixed effects, 𝑝<0.01).  11–13 percent increase in chance of PTB and/or LBW, using both models (CWS or mother fixed effects) shale gas development-related contamination increases 90 days after the drilling of the well (spud date). The contamination continues for up to 270 days after drilling and then returns to baseline.  These results lead us to believe that unconventional drilling has had an independent impact on birth outcomes through contamination of public drinking water.  Each additional well drilled during gestation within 1 km of a public water source increases the risk of LBW by about 0.71 percentage point and any well drilled within 1 km of the residence increases LBW by 0.68 percentage point. | |
| 9 | Janitz et al., 2019 | Newborns  US (Oklahoma)  476,600  1997 - 2009 | Birth defects – NTD, CCHD, and oral clefts by IDW tertile.  Oklahoma Birth Defects Registry. | Used modified Poisson regression with robust error variance to calculate prevalence proportion ratios and 95% CI for children with CCHD by UNGD activity tertiles in 2-mile buffer of birth residence compared to those with CCHDs and no UNGD activity within 2-mile buffer of birth residence. | Year of birth, sex, race/ethnicity, gestational age at delivery in weeks, birth weight in g, urban/rural status of census block at delivery, maternal age at delivery in yrs, marital status, prenatal care, parity, tobacco use during pregnancy, education were included in sensitivity model but maternal education only included in main model.  Race/ethnicity (non-Hispanic white, non-Hispanic African American, non-Hispanic American Indian/Alaska Native, non-Hispanic Asian/Pacific Islander, and Hispanic as reported by the mother).  Oklahoma Birth Defects Registry. 2000 US Census | Prevalence proportion ratio (95% CI) for NTD associated with IDW within 2-mile buffer:  1^st^ tertile: 0.89 (0.58, 1.37)  2^nd^ tertile: 1.34 (0.93, 1.93)  3^rd^ tertile: 1.20 (0.82, 1.75) | Researchers observed an increased prevalence of NTDs among children with natural gas activity compared to children with no wells.  Specific CCHDs were increased but not statistically significant  No association with oral cleft or total CCHDs  Researchers observed an increased, though imprecise, prevalence of NTDs among children with natural gas activity compared to children with no wells (2nd tertile prevalence proportion ratio (95% CI): 1.34 (0.93, 1.93); 3rd tertile: 1.20 (0.82, 1.75). Researchers observed no association with CCHD or oral clefts overall. Specific CCHDs of common truncus, transposition of the great arteries, pulmonary valve atresia and stenosis, tricuspid valve atresia and stenosis, interrupted aortic arch, and total anomalous pulmonary venous connection were increased among those living in areas with natural gas activity compared to those living in areas without activity, though not statistically significant. | |
| 10 | Li et al., 2022 | Individuals 65+ yrs  US (regions with UOGD)  15,198,496 (136,215,059 person yrs)  2001 - 2015 | Mortality.  Medicare beneficiary data. | Analyzed data using Cox proportional hazards and 2 difference in difference (DiD) designs.  Used robust sandwich variance estimators to account for the potential correlation of observations within the zip code in both models  Cluster-robust sandwich estimator used to account for the serial autocorrelation between repeated within-person measurements for the D - fitted a fixed-effects linear regression model that accounts for individual-level time-variant and time-invariant factors, a temporal trend in mortality, and zip-code-level time-variant covariates. | Age, sex, race, Medicaid eligibility, zip code level - median household income, average property value, % of population below the poverty line, % of population without high-school diplomas, population density and home ownership rate (time varying)  Conventional oil and gas exposure; annual PM_2.5_ concentrations; annual land cover data & yearly zip-code specific % of land surface covered by vegetation (to reflect enviro variation driven by changes in land use).  Medicare data 2000 and 2010 US Census & the American Community Survey behavioural risk variables from the Behavioral Risk Factor Surveillance System Enverus National spatiotemporal modeling  & land cover data from US geological survey. | Hazard Ratio (95% CI) for IDW proximity metric:  Low: 1.008 (1.004, 1.011)  Med-low: 1.013 (1.009, 1.017)  Med-high: 1.018 (1.014, 1.022)  High: 1.025: (1.021, 1.029)  Hazard Ratio (95% CI) for combined proximity and downwind metric:  Low, downwind: 1.010 (1.005, 1.014)  Low, upwind: 1.006 (1.001, 1.012)  Med-Low, upwind: 1.024 (1.018, 1.030)  Med-High, downwind: 1.013 (1.008, 1.018)  Med-High, upwind: 1.027 (1.022, 1.033)  High, upwind: 1.022 (1.017, 1.028)  High, downwind: 1.031 (1.025, 1.037) | We found evidence of a statistically significant higher mortality risk associated with living in proximity to and downwind of unconventional oil and gas wells.  We conclude that residential exposure to UOGD is positively associated with an elevated risk of all-cause mortality in the Medicare population, and airborne contaminants represent a key exposure pathway. | |
| 11 | McKenzie et al., 2014 | Newborns in rural Colorado (cities <50,000)  US (Colorado)  124,842  1996 - 2009 | Birth defects prevalence rates, CHDs, NTDs, oral clefts and perinatal outcomes – PTB, TBW, LBW by IDW tertile.  Colorado Responds to Children with Special  Needs birth registry, hospital records, the Newborn Genetics Screening Program, the Newborn Hearing Screening Program, laboratories, physicians, and genetic, developmental, and other specialty clinics, all included. | Multiple logistic and linear regression analysis. | Maternal age, education, tobacco use, ethnicity, and alcohol use, as well as parity at time of pregnancy and infant sex. Gestational age was also included in the analysis of TBW. Elevation of maternal residence by zip code and the year 1998 (year of introduction of folic acid fortification regulations) also was considered.  Covariate data from same source as outcome data. | OR (95% CI) for highest exposure tertile compared with absence of any gas wells within a 10-mile radius:  CHD prevalence:  1^st^ tertile : 1.1 (0.93, 1.3)  2^nd^ tertile : 1.2 (1.0, 1.3)  3^rd^ tertile: 1.3 (1.2, 1.5)  NTD:  1^st^ tertile : 0.65 (0.25, 1.7)  2^nd^ tertile : 0.80 (0.34, 1.9)  3^rd^ tertile: 2.0 (1.0, 3.9)  PTB:  1^st^ tertile : 0.96 (0.89, 1.0)  2^nd^ tertile : 0.93 (0.87, 1.0)  3^rd^ tertile: 0.91 (0.85, 0.98)  LBW:  1st tertile : 1.0 (0.9, 1.1)  2^nd^ tertile : 0.86 (0.77, 0.95)  3^rd^ tertile: 0.9 (0.9, 1.0)  Mean difference (95% CI) in TBW:  1^st^ tertile: +5 (–2.2, 13)  2^nd^ tertile: +24 (17, 31)  3^rd^ tertile: + 22 g (15, 29)  Positive linear trend (tested) for CHDs & NTDs.  Inverse linear (tested) for PTB, TBW and LBW. | Prevalence of CHDs increased with exposure tertile, with an OR (95% CI): 1.3 (1.2, 1.5); NTD prevalence was associated with the highest tertile of exposure (OR (95% CI): 2.0 (1.0, 3.9), n=59), compared with the absence of any gas wells within a 10-mile radius.  OR (95% CI) for ventricular septal defects 1.5 (1.1, 2.1) pulmonary artery and valve defects 1.6 (1.1, 2.2), and tricuspid valve defects 4.2 (1.3, 13) also associated with 3^rd^ tertile of IDW exposure.  No association between exposure and oral clefts.  Exposure was negatively associated with PTB and positively associated with fetal growth.  In this large cohort, we observed an association between density and proximity of natural gas wells within a 10-mile radius of maternal residence and prevalence of CHDs and possibly NTDs. | |
| 12 | Stacy et al., 2015 | All live births in 3 counties in Pennsylvania: Butler, Washington & Westmoreland)  US (Pennsylvania)  15,451  2007 - 2010 | Perinatal outcomes –PTB, SGA, TBW.  Pennsylvania Department of Health Bureau of Vital Statistics | Outcomes were modeled using multivariate linear regression (continuous birth weight) or logistic regression (SGA and prematurity) where 18% of total gas wells located. | Sex of the child, mother's age, education, pre-pregnancy weight, prenatal care, smoking status, gestational diabetes, WIC, African American and parity.  Race (% African American).  Pennsylvania Department of Health Bureau of Vital Statistics | Standardized β coefficient for TBW (ref = 1^st^ quartile)  Low: 0.01, *p=*0.27  Med: 0.00, *p=*0.96  High: -0.02, *p=*0.02  OR for SGA by quartile not available, but forest plots consistent with increasing OR across quartiles and % SGA across quartiles (ref = 1^st^ quartile)  2^nd^ quartile: 5.2%  3^rd^ quartile: 5.6%  4^th^ quartile: 6.5% | Comparing most to least exposed groups:  Significantly lower TBW (3323 +/- 558 g vs 3344 +/- 544 g).  Higher incidence of SGA (6.5% vs 4.8%); OR (95% CI) 1.34 (1.10, 1.63).  No association with PTB (“prematurity”).  A small but significant association between proximity to UGD and decreased birth weight was noted after accounting for a large number of contributing factors available from birth certificate data in Southwest Pennsylvania. | |
| 13 | Tran et al., 2020 | All births in the Sacramento Valley, San Joaquin Valley, South Central Coast, and South Coast air basins  US (California)  2,918,089  2006 - 2015 | Perinatal outcomes – LBW, PTB, TBW, SGA.  Birth records from the California Department of Public Health | Used generalized estimating equations to examine associations between overall and trimester-specific OGD exposures and outcomes by urban and rural populations. | Infant covariates: sex, month and year of birth.  Maternal covariates: age, race/ethnicity, educational attainment, smoking status, Kotelchuk index of prenatal care & parity, and gestational age for the TBW model.  Birth records from the California Department of Public Health  Area-level variables: indicators for air basin and census tract-based urban/rural status, modeled nitrogen dioxide concentrations, and a measure of income concentration.  California Air Resources Board, US, Census Bureau 2010, Index of Concentration at the Extremes | In rural areas only, production volume metric (barrels of oil equivalent) OR for overall pregnancy exposure of: OR (95% CI):  LBW: ref = Low exposure  Med:1.11 (0.97, 1.27)  High: 1.40 (1.14, 1.71)  SGA: ref = Low exposure  Med: 1.07 (0.97, 1.19)  High: 1.22 (1.02, 1.45)  PTB: ref = Low exposure  High: 1.03 (0.91, 1.18)  TBW (mean difference g, 95% CI): ref = Low  Med: 3 g (-11, 18)  High:−36 g (−54, −17) | In rural areas, increasing production volume associated with stronger adverse effect estimates. High (>100 barrels of oil equivalent / day) vs. no production throughout pregnancy was associated with increased odds of LBW (OR (95% CI): 1.40 (1.14, 1.71) & SGA (OR (95% CI): 1.22 (1.02, 1.45), & decreased TBW (mean difference (95% CI): −36 g (−54, −17)), but not with PTB (OR (95% CI): 1.03 (0.91, 1.18)).  Proximity to higher production OGD in California was associated with adverse birth outcomes among mothers residing in rural areas.  Proximity to higher production OGD in California was associated with adverse birth outcomes among mothers residing in rural areas. | |
| 14 | Tran et al., 2021 | Exposed individuals with at least 1 well hydraulically fractured within 1 km of their residence during pregnancy in 8 counties where fracking takes place  US (California)  979,961  2006 - 2015 | Perinatal outcomes – TBW, LBW, SGA PTB.  Birth records from the California Department of Public Health | Used generalized estimating equation to account for clustering within census tracts; separate models for each of our four birth outcomes to assess the association between prenatal exposure to hydraulic fracturing and odds of lower TBW, LBW, SGA, PTB.  Stratified models by urban and rural. | Individual covariates: sex, month and year of conception, maternal age, self-reported race, educational attainment, index of prenatal care, and parity; and for TBW added mean-centered and mean-centered squared gestational age (continuous) to allow for nonlinearity.  Area-level covariables: California Air Resources  Board designated air basins, census-tract based urban-rural classification, dioxide (NO_2_ pollution; Modeled annual average nitrogen as proxy for traffic-related air concentrations and Index of Concentration at the Extremes; measure of neighborhood-level relative deprivation or affluence. | Rural population adjusted models OR (95% CI):  TBW: -73 g (-131, -15)  LBW: 1.74 (1.10, 2.75)  SGA: 1.68 (1.42, 2.27)  PTB: 1.17 (0.64, 2.12) | Fewer than 1% of mothers (N=1,192) were exposed to hydraulic fracturing during pregnancy.  Among rural mothers, hydraulic fracturing exposure was associated with increased odds OR (95% CI) of LBW 1.74, (1.10, 2.75), SGA 1.68 (1.42, 2.27) and PTB 1.17 (0.64, 2.12), and lower TBW (mean difference (95% CI) –73 g (–131, –15)).  Among urban mothers, hydraulic fracturing exposure was positively associated with SGA (OR (95% CI): 1.23 (0.98, 1.55)), inversely associated with LBW (0.83 (0.63, 1.07)) and PTB (0.65 (0.48, 0.87)), and not associated with TBW (mean difference (95% CI): –2 g (–35, 31)).  Conclusion: hydraulic fracturing proximity was associated with adverse birth outcomes, particularly among rural Californians. | |
| 15 | Whitworth et al., 2017 | Newborns or fetal deaths that occurred in 24 counties in the Barnett Shale region of Texas  US (Texas)  158,894  2010 - 2012 | Perinatal outcomes – PTB, SGA, TBW, fetal death.  Texas Department of State Health Services birth records. | Used logistic or linear regression with generalized estimating equations to assess associations between UGD-activity and PTB, SGA, fetal death, or birthweight. | Fetal death and TBW outcomes adjusted for maternal age, education, parity, smoking during pregnancy, race/ethnicity, pre-pregnancy BMI, infant sex, and previous poor pregnancy outcome. PTB & SGA adjusted for the above but excluded parity and previous poor outcomes respectively.  Constructed the Adequacy of Prenatal Care Utilization Index which captures timing of first prenatal visit and frequency of visits.  Texas Department of State Health Services birth records.  Dichotomous variable for distance between maternal residence and nearest major roadway.  ArcMap | OR (95% CI) within ½-mile buffer, ref = 0 wells < 10-mile  PTB:  1^st^ tertile: 1.18 (1.08, 1.29)  2^nd^ tertile: 1.21 (1.09, 1.33)  3^rd^ tertile: 1.14 (1.03, 1.25)  OR (95% CI) within 2-mile buffers:  PTB:  1^st^ tertile: 1.11 (1.04, 1.19)  2^nd^ tertile: 1.16 (1.09, 1.24)  3^rd^ tertile: 1.14 (1.07, 1.22)  Fetal death:  1^st^ tertiles: 1.14 (0.83, 1.56)  2^nd^ tertile: 1.56 (1.16, 2.11)  3^rd^ tertile: 1.16 (0.86, 1.58)  OR (95% CI) within 10-mile buffer:  PTB:  1^st^ tertile: 1.02 (0.96, 1.08)  2^nd^ tertile: 1.13 (1.06, 1.20)  3^rd^ tertile: 1.15 (1.08, 1.22)  Fetal death:  1^st^ tertile:: 1.26 (0.99, 1.60)  2^nd^ tertile: 1.22 (0.95, 1.57)  3^rd^ tertile: 1.34 (1.04, 1.72) | Odds (OR (95% CI)) of PTB in highest exposure tertile, by distance to well:  Within ½ mile: 1.14 (1.03, 1.25)  Within 2 miles: 1.14 (1.07, 1.22)  Within 10 miles: 1.15 (1.08, 1.22)  Risk (OR (95% CI)) of fetal death, by distance to well:  Within 2 miles - 2^nd^ tertile: 1.56 (1.16, 2.11)  Within 10 miles - highest tertile: 1.34 (1.04, 1.72)  No association of exposure and SGA, TBW.  Results are suggestive of an association between maternal residential proximity to UGD-activity and PTB and fetal death. | |
| 16 | Willis et al., 2021 | Maternal-infant pairs living <10 km from an oil or gas site  US (Texas)  2,598,025  1996 - 2009 | Perinatal outcomes –SGA, TBW.  Birth-certificate data from Texas Department of State Health Services. | Difference in difference linear and logistic-regression models with robust standard errors, examined the interaction between indicator for residential location within a buffer (0-1 km, 1-2 km, & 2-3 km) of one or more drilling sites and indicator for delivery date after drilling began. | Individual covariates: infant sex. maternal: age, race, education level, weight gain during pregnancy, nulliparity, gestational hypertensions, eclampsia, diabetes, prenatal care, smoking during pregnancy, county of residence at time of delivery, rural residence, nearest highway to residence  Birth-certificate data from Texas Department of State Health Services  Neighbourhood level: median household income, unemployment, total white population, total population.  .US Census Bureau 2000 | Difference in difference estimate in g (95% CI) for TBW, for during active vs future drilling sites with 3-10 km as ref group:  0-1 km: −7:3 (−11:6, −3:0)  1-2 km: −8:9 (−13:1, −4:8)  2-3 km: −9:3 (−14:1, −4:5)  TBW inverse association stronger for conventional vs. unconventional drilling in all models.  Difference in difference estimate (95% CI) for SGA  0-1 km: 1.02 (0.98, 1.06)  1-2 km: 1.02 (0.99, 1.06)  2-3 km: 1.02 (0.98, 1.07) | Adjusted mean difference (95% CI) in TBW for mothers living 0–1 vs. 3–10 km from a current or future drilling site was –7:3 g (–11:6, –3:0) for births during active vs. future drilling and for SGA was 1.02 (0.98, 1.06).  Negative associations with TBW were observed for the 1–2 and 2–3 km near groups, with no consistent differences identified by type of drilling activity.  Larger, though imprecise, adverse associations were found for infants born to Hispanic women, women with the lowest educational attainment, and women living in cities.  Residing near oil and gas drilling sites during pregnancy was associated with a small reduction in TBW but not SGA.  No consistent differences were identified by type of drilling activity. | |
| 17 | Willis et al., 2022 | All pregnant women in Texas where mothers reside <10 km from an active or future UOG drilling site  US (Texas)  2,845,144  1996 - 2009 | Gestational hypertension, eclampsia.  Birth-certificate data from Texas Department of State Health Services. | Difference in difference logistic-regression models with robust standard errors, examined the interaction between indicator for residential location within a buffer (0-1 km, 1-2 km, & 2-3 km) of one or more drilling sites and indicator for delivery date after drilling began | Maternal covariates: age, weight gain during pregnancy, race, ethnicity, educational attainment, birth month, year of birth  Neighbourhood level for: % white population, median household income  Sourced from birth certificate, census tract. | OR (95% CI) for women living proximal to active vs future drilling sites:  Gestational hypertension:  0-1 km 1.05 (1.00, 1.10)  1-2 km 0.99 (0.95, 1.04)  2-3 km 1.00 (0.94, 1.05)  Eclampsia:  0-1 km 1.26 (1.05, 1.51)  1-2 km 1.10 (0.92, 1.32)  2-3 km 0.97 (0.78, 1.21) | Among pregnant women residing 0–1 km from an active oil or gas extraction site, estimated 5% increased odds (95% CI) of gestational hypertension (1.00, 1.10) and 26% increased odds (95% CI) of eclampsia (1.05, 1.51) in adjusted models. This association dissipates in the 1- to 3 km buffer zones. In restricted models, we find elevated OR among maternal ages 35 yrs at delivery, maternal non-Hispanic White race, 30 lbs gained during pregnancy, nulliparous mothers and maternal educational attainment beyond high school.  Living within 1 km of an oil or gas extraction site during pregnancy is associated with increased odds of hypertensive conditions during pregnancy. | |
| 18 | Willis et al., 2023 | All births  US (Texas)  2,234,138 births, 86,315 identified with an anomaly  1999 - 2009 | Any congenital anomaly, 1+ congenital anomaly, cardiac & circulatory, central nervous system, eye & ear, gastrointestinal, musculoskeletal system, oral cleft, respiratory, chromosomal congenital anomalies.  Texas Vital Statistics database and Birth Defects Registry. | Logistic regression with spatial ref group (distance within 9-19 km of active well vs more proximal divided into 1 km group) & with temporal ref group (IDW with 5km buffer vs maternal residences with future (but no active) drilling sites within 5 km as a comparison group. | Birth year, county of maternal residence at delivery infant sex gestational age birth weight, maternal age maternal race & ethnicity, maternal education, smoking prenatal care initiated, distance to nearest highway, unemployment rate median household income, % white population .  Texas Vital Statistics database & census tract data. | Model with ref group of maternal residence w/future but no active drilling sites within 5 km (spatial ref)  Any congenital OR (95% CI)  Site count:  1^st^ tertile: 1.19 (1.15,1.22)  2^nd^ tertile: 1.24(1.19, 1.28)  3^rd^ tertile: 1.25 (1.21, 1.30)  Model with ref group active drilling sites at 5-10 km (temporal ref)  Any congenital anomaly:  1^st^ tertile: 1.01 (0.98, 1.03)  2^nd^ tertile: 1.04 (1.02, 1.06)  3^rd^ tertile: 1.05 (1.03, 1.08)  Model with ref group of maternal residence w/future but no active drilling sites within 5 km  IDW Oil production  1^st^ tertile: 1.10 (1.07, 1.13)  2^nd^ tertile: 1.08 (1.05, 1.11)  3^rd^ tertile: 1.08 (1.04, 1.12)  IDW gas productions:  1^st^ tertile: 1.14 (1.11, 1.18)  2^nd^ tertile: 1.13 (1.10, 1.17)  3^rd^ tertile: 1.20 (1.17, 1.23)  IDW produced water:  1^st^ tertile: 1.14 (1.11, 1.17)  2^nd^ tertile: 1.14 (1.10, 1.17)  3^rd^ tertile: 1.17 (1.14, 1.20)  Similar but attenuated result for models using spatial comparator.  Cardiac and circulatory defects, were consistently associated with exposures related to oil and gas extraction. | Using the temporal comparison group, increased odds of any congenital anomaly in the highest tertile exposure group for site count (OR (95% CI): 1.25 (1.21, 1.30)), oil production (1.08 (1.04, 1.12), gas production (1.20 (1.17, 1.23), and produced water (1.17 (1.14, 1.20).  Associations did not follow a consistent exposure-response pattern across tertiles.  Associations are highly attenuated, but still increased, with the spatial comparison group in the highest tertile exposure group.  Cardiac and circulatory defects are strongly and consistently associated with all exposure metrics.  Increased odds of congenital anomalies, particularly cardiac and circulatory defects, were associated with exposures related to oil and gas extraction in this large population-based study. | |
|  | ***Case Control Studies*** | | | | | | | |
| 19 | Clark et al., 2022 | All children ages 2-7 yrs diagnosed with ALL in Pennsylvania between 2009 & 2017, and controls matched on birth year.  US (Pennsylvania)  405 cases  2080 controls  2009 - 2017 | Childhood cancer diagnosis of ALL.  Children ages 2-7 yrs diagnosed with ALL identified from the Pennsylvania state cancer registry.  Five control children randomly selected from live births in the Pennsylvania birth records with frequency-matching on birth year. | Minimally, parsimoniously and highly adjusted non conditional logistic models to assess association of 2 exposure measures, 2 exposure windows - 3 months prior to conception to 1 year prior to diagnosis (primary) and 3 months prior to conception to birth (perinatal) with outcome. | Depending on model, adjustment for: year of birth, maternal race and collection of WIC.  Other covariates include: sex, gestational age, birthweight, mode of delivery, maternal ethnicity, educational achievement, and marital status census tract level median household income, % cropland within 500 m of home, and census tract centroid annual PM_2.5_ (only included in minimal and parsimonious modeling if OR changed by less than 10%), social vulnerability index  Birth records, cancer registry, US Environmental Protection Agency, Agriculture National Agricultural Statistics Service CropScape, US 2000 and 2010 Decennial Census, Centers for Disease Control and Prevention/Agency for Toxic Substances and Disease Registry Social Vulnerability Index. | Primary Exposure (3 months to one year) prior to diagnosis) window OR (95% CI) for ALL associated with ID^2^W well counts:  Within 2 km:  1.98 (1.06, 3.69) (minimally adjusted)  1.74 (0.93, 3.27) (parsimoniously adjusted)  Within 5 km:  1.33 (0.88,2.00) (minimally adjusted)  1.18 (0.78,1.78) (parsimoniously adjusted)  Within 10 km:  1.14 (0.84,1.55) (minimally adjusted)  1.03 (0.76,1.41) (parsimoniously adjusted)  Perinatal exposure (3 months pre-conception to birth) window: OR (95% CI) for ALL associated with ID^2^W well counts:  Within 2 km:  2.80 (1.11, 7.05) (minimally adjusted)  2.35 (0.93, 5.95) (parsimoniously adjusted)  Within 5 km:  1.54 (0.90, 2.63) (minimally adjusted)  1.38 (0.80, 2.37) (parsimoniously adjusted)  Within 10 km:  1.42 (0.99, 2.04) (minimally adjusted)  1.31 (0.91, 1.89) (parsimoniously adjusted)  The OR using the water pathway-specific metric were similar in magnitude to aggregate metric. | Children with at least one UOG well within 2 km of their birth residence during the primary window had 1.98 times the odds of developing ALL in comparison with those with no UOG wells (95% CI: 1.06, 3.69). Children with at least one vs. no UOG wells within 2 km during the perinatal window had 2.80 times the odds of developing ALL (95% CI: 1.11, 7.05). These relationships were slightly attenuated after adjusting for maternal race and socio-economic status (OR =1:74 (95% CI: 0.93, 3.27) and OR=2:35 (95% CI: 0.93, 5.95)), respectively). The OR produced by models using the water pathway-specific metric were similar in magnitude to the aggregate metric.  Study including a novel UOGD metric found UOGD to be a risk factor for childhood ALL.  Results suggest that preconception to birth is an important etiological window for exposure to UOG and the development of ALL.  Given the similar results observed across both exposure windows, findings suggest that UOG-related environmental exposures may contribute to both prenatal and postnatal insults leading to the development of ALL. | |
| 20 | Elser et al., 2021 | All prevalent migraine cases identified in Sutter Health Electronic Records, frequency matched to controls in 3:1 ratio on age, sex, year of entry into Sutter primary care & primary care follow-up time  US (N. California)  89,575 cases  270,564 controls  2014 - 2018 | Migraine (yes/no) and migraine severity score.  Sutter Health Electronic Records. | Case control: general linear models with logit link with county-specific random intercepts for potential within-county clustering.  Case case: negative binomial mixed models & logistic mixed models with random intercepts for county.  4 separate models examined association between migraine status and: methane super-emitters, active oil and gas wells, PM_2.5_, NO_2_. | Individual level: age, sex, year of entry into Sutter primary care, length of primary care follow-up: race/ethnicity, Medicaid use, number of primary care visits per year.  Block group-level population density and poverty.    Sensitivity analyses also adjusted for BMI category and marital status.  Sutter Health Electronic Records American Community Survey. | OR (95% CI) for continuous environmental exposure and migraine diagnosis:  Per each 100,000 kilogram/hour increase in IDW methane emissions: 1.04 (1.00, 1.08)  Per 1,000 unit increase in IDW sum of active oil & gas wells: 0.99 (0.99, 1.00)  Per 5 µg/m^3^ increase in PM_2.5_ levels: 1.00 (0.97, 1.04)  Per OR corresponds to a 5 ppb increase in NO_2_ levels: 1.02 (1.00, 1.05)  RR (95% CI) for continuous exposures and migraine severity measures:  PM_2.5_:  Triptan Rx: 1.01 (0.99, 1.02)  Neuro visit: 1.18 (1.09, 1.29)  Urgent care visit: 3.09 (2.28, 4.18)  ED visits: non linear  Migraine Probability Algorithm (MPA) score: 1.14 (1.07, 1.22)  NO_2_:  Triptan Rx: 1.01 (0.98, 1.06)  Neuro visit: 0.99 (0.94, 1.05)  Urgent care visit: 1.22 (1.02, 1.46)  ED visits: 1.16 (1.05, 1.29)  MPA score: 1.00 (0.96, 1.05) | In adjusted analyses, for each 5 ppb increase in NO_2_, we observed 2% increased odds of migraine case status (95% CI: 1.00, 1.05) and for each 100,000 kg/hour increase in IDW methane emissions, the odds of case status also increased (OR (95% CI): 1.04 (1.00, 1.08).  We found no association between PM_2.5_ or oil and gas wells and migraine case status. PM_2.5_ was linearly associated with neurology visits, migraine-specific urgent care visits, and MPA score > 100, but not triptans or ED visits. NO_2_ was associated with migraine-specific urgent care and ED visits, but not other severity measures. We observed limited or null associations between continuous measures of methane emissions and proximity to oil and gas wells and migraine severity.  Our findings illustrate the potential role of long-term exposure to multiple ambient air pollutants for prevalent migraine and migraine severity. | |
| 21 | Koehler et al., 2018 | All primary care patients within the Geisinger health system in PA with a diagnosis of asthma frequency matched to patients with asthma with no exacerbations by age, sex and year of encounter  US (Pennsylvania)  35,508  2005 - 2012 | Mild asthma exacerbations based on new oral corticosteroid medication order.  Geisinger Health System. | Used multilevel logistic regression with random intercept for patient and community to account for multiple events per patient and patient clustering within communities to assess the association of activity metrics and the mild asthma exacerbation  Used principal components analysis on UNGD activity metrics to find components, linear combinations of the different variables in the dataset that explain the highest proportion of the variability in the dataset. | Age, sex, race/ethnicity, family history of asthma, smoking status, season, medical assistance, overweight/obesity status, distance and distance-squared to nearest major and minor arterial roads, maximum temperature and maximum temperature-squared on the day prior to the event, & community socioeconomic deprivation.  See Rasmussen, 2016 | OR (95% CI) for distance to nearest drilled well (DNDW) measurement:  >2 km: 1.0 (ref)  1-2 km: 1.13 (0.76, 1.69)  <1 km: 1.83 (1.03, 3.25)  OR (95% CI) for IDW1 measurement:  No wells within 10-mile (ref)  1^st^ tertile: 0.96 (0.83, 1.13)  2^nd^ tertile: 1.21 (1.03, 1.42)  3^rd^ tertile: 1.19 (1.01, 1.41)  OR (95% CI) for IDW24C measurement:  1^st^ quartile: (ref)  2^nd^ quartile: 1.31 (1.16, 1.48)  3^rd^ quartile: 2.20 (3.16, 4.30)  4^th^ quartile: 3.69 (3.16, 4.30)  OR (95% CI) for IDW2P measurement:  1^st^ quartile: (ref)  2^nd^ quartile: 1.28 (1.13, 1.46)  3^rd^ quartile: 2.15 (1.87, 2.47)  4^th^ quartile: 4.43 (3.75, 5.22)  Did not include impoundments in the final principal components analysis model used to assign the UNGD activity to subjects because impoundment data were not available for all of the yrs. | The three metrics had varying magnitudes of association with mild asthma exacerbations, although the highest category of each metric (vs the lowest) was associated with the outcome.  IDW24C (composite metric of approximately equal mix of the metrics for compressors, and four phases of well development (pad preparation, drilling, stimulation, and production).) metric was most strongly associated with mild asthma exacerbations (with a magnitude of association twice that of the previously reported IDW1 and distance to nearest drilled well (DNDW metric) and the IDW1 metric was the least strongly associated.  Association was not as strong for the IDW24C, as we previously found for a metric that only incorporated the production phase.  A principal component analysis identified a single component that was approximately an equal mix of the metrics for compressors, impoundments, and four phases of well development (pad preparation, drilling, stimulation, and production).  Given the small potential benefits of including flaring and impoundment data in UNGD activity metrics, it does not seem to be worth the time and effort required to obtain the data. We recommend that researchers use the IDW24C metric, when possible. | |
| 22 | McAlexander et al., 2020 | All patients with a diagnosis of heart failure  US (Pennsylvania)  12,330  2008 - 2015 | Incident hospital admission for heart failure.  Geisinger Health System. | Used multilevel logistic regression models estimating the odds of hospitalization, comparing cases to controls, by quartile of UNGD activity. Used patient (to account for correlation within individuals over time who were included in analysis more than once as control then as case) and community (to account for the correlation of measures for persons clustered in communities). Retained variables in the model if they changed the effect estimates for any of the 4 UNGD activity metrics by more than 5%.  Evaluated effect modification by heart failure phenotype (HFpEF: reduced ejection fraction, HFrEF: reduced ejection fraction) and severity. | Initially included a priori: sex (female vs. male), age category, smoking status, race/ethnicity (non-Whites, White), BMI, and medical assistance.  Additional models evaluated, in stepwise fashion, year of hospitalization or control selection date; geographic region; year and region; season, duration of health care contact; distance to nearest hospital or clinic; and distance to major and minor roads.  Geisinger Health System  Census tract data  2010 – 2014 Census American Community Survey  National Aeronautics & Space Administration satellite data | OR (95% CI) for UOGD hospital admission, by well phase (1^st^ quartile as reference):  Pad prep:  2^nd^ quartile: 1.19 (1.01, 1.40)  3^rd^ quartile: 1.63 (1.35, 1.97)  4^th^ quartile: 1.70 (1.35, 2.13)  Spud:  2^nd^ quartile: 1.01 (0.82, 1.25)  3^rd^ quartile: 1.07 (0.85, 1.35)  4^th^ quartile: 0.97 (0.75, 1.27)  Stimulation:  2^nd^ quartile: 1.03 (0.81, 1.31)  3^rd^ quartile: 1.56 (1.19, 2.04)  4^th^ quartile: 1.80 (1.35, 2.40)  Drilling:  2^nd^ quartile: 0.87 (0.64, 1.19)  3^rd^ quartile: 1.10 (0.75, 1.60)  4^th^ quartile: 1.62 (1.07, 2.45) | We identified 9,054 patients with heart failure with 5,839 hospitalizations (mean age 71.1 +/- 12.7 years; 47.7% female). Comparing 4th to 1st quartiles, adjusted odds ratios (95% confidence interval) for hospitalization were 1.70 (1.35 to 2.13), 0.97 (0.75 to 1.27), 1.80 (1.35 to 2.40), and 1.62 (1.07 to 2.45) for pad preparation, drilling, stimulation, and production metrics, respectively. We did not find effect modification by HFrEF or HFpEF status. Associations of most UNGD metrics with hospitalization were stronger among those with more severe heart failure at baseline.  Three of 4 phases of UNGD activity were associated with hospitalization for UOGD in a large sample of patients with UOGD in an area of active UNGD, with similar findings by HFrEF versus HFpEF status, although HFrEF demonstrated higher magnitudes of effect estimates. And associations were stronger in patients with more severe hydraulic fracturing. Older patients with UOGD seem particularly vulnerable to adverse health impacts from UNGD activity. | |
| 23 | McKenzie et al., 2017 | All children from 0-24 yrs living in rural Colorado who were diagnosed with cancer. Cases were diagnosed with ALL and non-Hodgkin lymphoma, controls were diagnosed with other cancers  US (Colorado)  137 cases  528 controls  1991 - 2013 | Childhood hematological cancers –ALL and non-Hodgkin lymphoma.  Colorado Central Cancer Registry (CCCR) [23] held at the Colorado Department of Public Health and Environment (CDPHE) | Used logistic regression to study associations between case-control status and IDW well count tertiles. Conducted tests to evaluate linear trends in binominal proportions with increasing IDW well count by treating the categorical IDW well count variable as ordinal. | Age, race, gender, elevation of residence, socioeconomic status, year of diagnosis.  Elevation of residence; zip-code level socioeconomic status  Colorado Central Cancer Registry (CCCR) [23] held at the Colorado Department of Public Health and Environment (CDPHE) | OR (95% CI) for ALL and exposure with 0 wells within 16.1 km as the reference group for children 5-24 yrs:  Low: 2.9 (0.80, 11)  Med: 3.4 (0.99, 12)  High: 4.6 (1.2, 18)  No significant association of exposure and ALL for total study population or for 0-4 yrs.  No significant association of exposure and non-Hodgkin lymphoma.  The ALL cases were more likely than controls to be < 10 yrs of age, male, a non-white race, to live at an elevation > 2743 meters, and in the highest zip code median income quintile.  Positive linear trend (tested) for ALL in children 5-24 yrs. | For ages 5–24 yrs, ALL cases were 4.3 times as likely to live in the highest tertile, compared to controls (95% CI: 1.1 to 16), with a monotonic increase in risk across tertiles (trend p-value = 0.035). While our study benefited from the ability to select cases and controls from the same population, use of cancer-controls, the limited number of ALL and non-Hodgkin lymphoma aggregation of ages into five-year ranges, may have biased our associations toward the null. | |
| 24 | McKenzie et al., 2019a | All live singleton births in 34 Colorado counties with 20 or more wells drilled  US (Colorado)  469 cases  2,860 controls  2004 - 2011 | Total CHDs, an aortic artery and valve (AAVD), pulmonary artery and valve, conotruncal, or tricuspid valve defect  Colorado Department of Public Health and Environment's (CDPHE) Center for Health and Environmental Data; Colorado Responds to Children with Special  Needs birth defects registry: cases with CHD; birth certificate data: controls | For each of the five months between three months prior to conception through the second month of gestation, we used unconditional logistic regression to evaluate associations between each dichotomous outcome (combined CHDs, AAVD, pulmonary artery and valve defect, cardiovascular disease, and tricuspid valve defect) and intensity-adjusted inverse distance weighted group with the low group as the referent.  Conducted tests to evaluate linear trends in binominal proportions with increasing intensity-adjusted inverse distance weighted level | Maternal age, maternal race, socioeconomic status, parity, maternal smoking, and sex.; O&G facilities other than wells; air pollution sources not associated w/ O&G facilities  Tested for effect modification of residence in rural zip code, infant sex and 2-mile buffer.  Colorado Responds to Children with Special  Needs birth defects registry: birth certificate data: controls  Colorado  Oil and Gas Information System; Toxic Release Inventory and Solid Waste, and Wastewater Treatment Facility; Colorado Oil and Gas Information System; US Geological Survey National Mines Information Center; CDPHE's Concentrated Animal Feeding Operations, and Composting, Solid Waste, and Wastewater Treatment Facility data | OR (95% CI) for medium and high exposure vs low (ref) within 10-mile radius of maternal residence in 2^nd^ month of pregnancy, total population:  Any CHD:  Med: 1.4 (1.0, 2.0)  High: 1.7 (1.1, 2.6)  Pulmonary artery and valve defect:  Med: 1.4 (0.87, 2.3)  High: 1.7 (0.87, 3.2)  In rural areas only:  AAVD:  Med: 1.8 (0.97, 3.3)  High: 2.6 (1.1, 6.1)  Conotruncal defect  Med: 2.1 (0.96, 4.5)  High: 4.0 (1.4, 12)  Tricuspid valve defect  Med: 3.4 (0.95, 12)  High: 4.6 (0.81, 27)  Positive linear trend (tested) for CHD (total population), CHD, AAVD, conotruncal defects (rural population). | CHDs were 1.4 (1.0, 2.0) and 1.7 (1.1, 2.6) times more likely than controls in the medium and high intensity groups, respectively, compared to the low intensity group.  In rural areas, AAVDs, conotruncal defects, and tricuspid valve defects were 1.8 (0.97, 3.3) and 2.6 (1.1, 6.1); 2.1 (0.96, 4.5) and 4.0 (1.4, 12); and 3.4 (0.95, 12) and 4.6 (0.81, 26) times more likely than controls in the medium and high intensity groups.  This study provides further evidence of a positive association between maternal proximity to O&G well site activities and several types of CHDs, particularly in rural areas. | |
| 25 | Rasmussen et al., 2016 | Individuals aged 5-90 yrs with asthma living in highly fracked regions of the Marcellus Shale  US (Pennsylvania)  35,508 cases + controls  Matched ~4:1, 6:1, 1:1 for severe, moderate and mild exacerbations respectively.  2005 - 2012 | Individuals with asthma who did (cases) or did not experience (controls) mild (new prescription for oral corticosteroid), moderate (emergency department visit) or severe (hospital admission) exacerbations.  Electronic health records from Geisinger clinic | Used multilevel logistic regression with random intercept for patient and community to assess the association of 4 UNGD activity metrics and 3 types of asthma exacerbations. | Age, sex, race/ethnicity, family history of asthma, smoking status, season, medical assistance, obesity, type 2 diabetes, community socioeconomic deprivation, distance to nearest arterial road, maximum temperature of day prior to assessment.  Electronic health records from Geisinger clinic  Federal Highway Administration data  Weather station and census tract data | OR (95% CI)  Severe exacerbation (hospital admission): Ref = lowest quartile  Pad preparation::  Low: 1.26 (1.06, 1.50)  Med: 1.37 (1.15, 1.64)  High: 1.45 (1.21, 1.73)  Drilling (Spud):  Low: 1.16 (0.98, 1.37)  Med: 1.26 (1.05, 1.50)  High: 1.64 (1.38, 1.97)  Stimulation:  Low: 1.13 (0.96, 1.33)  Med: 1.31 (1.10, 1.57)  High: 1.66 (1.38, 1.98)  Productions:  Low: 1.10 (0.92, 1.30)  Med: 1.16 (0.97, 1.38)  High: 1.74 (1.45, 2.09)  Mild exacerbation (oral corticosteroid Rx):  Pad metric:  Low: 1.54 (1.37, 1.74)  Med: 1.66 (1.47, 1.87)  High: 1.59 (1.41, 1.81)  Spud:  Low: 1.45 (1.29, 1.63)  Med: 1.98 (1.75, 2.24)  High: 1.99 (1.75, 2.26)  Stimulation:  Low: 1.23 (1.09, 1.39)  Med: 2.22 (1.95, 2.53)  High: 3.00 (2.60, 3.45)  Productions:  Low: 1.28 (1.13, 1.46)  Med: 2.15 (1.87, 2.47)  High: 4.43 (3.75, 5.22) | There was an association between the highest group of the activity metric for each UNGD phase compared with the lowest group for 11 of 12 UNGD-outcome pairs: OR (95% CI) ranged from 1.5 (1.2, 1.7) for the association of the pad metric with severe exacerbations to 4.4 (3.8, 5.2) for the association of the production metric with mild exacerbations.  Six of the 12 UNGD-outcome associations had increasing OR across quartiles.  Residential UNGD activity metrics were statistically associated with increased risk of mild, moderate, and severe asthma exacerbations.  Patients with moderate exacerbations were more likely to be on medical assistance and of black race than patients with the other 2 outcomes. | |
| 26 | Tang et al., 2021 | Cases from all pregnancy outcomes (live births, spontaneous fetal deaths, & pregnancy terminations) with a confirmed diagnosis of a birth defect made prenatally or within one year after delivery.  Controls from spatially stratified random sampling controls from all live births in 1:2 ratio.  US (Texas)  695,354  1999 - 2011 | Selected NTDs, CHDs, and orofacial clefts primarily as individual birth defects, and gastroschisis stratified by age.  Texas Birth Defects Registry. | Used logistic regression models to calculate crude and adjusted OR and 95% CI for the association between each birth defect outcome group and UNGD exposure for a given radius, controlling for maternal characteristics and neighborhood factors | Maternal smoking status, plurality of birth, maternal age, maternal race/ethnicity, education status, neighborhood median household income at maternal address block group, urban city, and average daily vehicle miles traveled for all trucks by county.  Center for Health Statistics at the Texas Department of State Health Services.  US Census American Community Survey. | OR (95% CI), ref = 0 wells  Anencephaly, 1 km buffer  1^st^ tertile: 0.72 (0.41, 1.29)  2^nd^ tertile: 1.02 (0.66, 1.59)  3^rd^ tertile: 2.94 (1.83, 4.75)  with similar pattern for 3 km & 7.6 km buffers  Spina bifida, 1 km buffer:  1^st^ tertile: 1.37 (1.01, 1.86)  2^nd^ tertile: 0.94 (0.69, 1.29)  3^rd^ tertile: 2.09 (1.47, 2.99)  with similar pattern for 3 km & 7.6 km buffers  Gastroschisis (maternal age>24 yrs), 1 km buffer:  1^st^ tertile: 2.21 (1.00, 4.92)  2^nd^ tertile: 1.54 (0.87, 2.73)  3^rd^ tertile: 3.19 (1.77, 5.73)  3 km buffer:  1^st^ tertile: 1.07 (0.81, 1.41)  2^nd^ tertile: 1.52 (0.93, 2.46)  3^rd^ tertile: 0.98 (0.50, 1.91)  Aortic valve stenosis, 1 km buffer:  1^st^ tertile: 1.37 (0.96, 1.96)  2^nd^ tertile: 1.51 (1.06, 2.15)  3^rd^ tertile: 1.90 (1.3, 2.71)  Hypoplastic left heart syndrome, 1 km buffer:  1^st^ tertile: 0.97(0.63, 1.50)  2^nd^ tertile: 1.81 (1.29, 2.55)  3^rd^ tertile: 2.00 (1.39, 2.86)  Pulmonary valve atresia/ stenosis, 1 km buffer:  1^st^ tertile: 1.30 (1.08, 1.56)  2^nd^ tertile: 1.49 (1.25, 1.78)  3^rd^ tertile: 1.36 (1.10, 1.66)  with similar pattern for 3 km & 7.6 km buffers  Positive linear trend (tested) for many outcomes within and across buffers (anencephaly, 1km buffer; atrial septal defect, aortic valve stenosis, hypoplastic left heart syndrome, pulmonary valve stenosis, all buffers). | Positive association s were observed between the highest tertile of UNGD density within 1 km of maternal address and risk of anencephaly (OR: 2.44, 95% CI: 1.55, 3.86), spina bifida (OR: 2.09, 95% CI: 1.47, 2.99), gastroschisis among older mothers (OR: 3.19, 95% CI: 1.77, 5.73), aortic valve stenosis (OR: 1.90, 95% CI: 1.33, 2.71), hypoplastic left heart syndrome (OR: 2.00, 95% CI: 1.39, 2.86), and pulmonary valve atresia or stenosis (OR: 1.36, 95% CI: 1.10, 1.66). For CHD subtypes, results did not differ substantially by distance from maternal address or when residual confounding was considered, except for atrial septal defects.  We did not observe associations with orofacial clefts. Our results suggest that UNGDs were associated with some CHDs and possibly NTDs. In addition, we identified temporal trends and observed presence of spatial residual confounding for some CHDs. | |
| 27 | Walker Whitworth and Symanski, 2018 | All singleton births in the Barnett Shale region in Texas  US (Texas)  81,294  2010 - 2012 | PTB cases were matched to term births for age and race/ ethnicity in a 1:5 ratio.  Perinatal outcomes – phase and trimester specific PTB (extremely–trimester 1, very–trimester 2, and moderately–trimester 3 preterm).  Texas Department of State Health Services, Center for Health Statistics birth records | Used conditional logistic regression to examine the association between UGD drilling and production metrics and PTB during the entire pregnancy and for each trimester. | Matched on age and race/ethnicity.  Adjusted for pre-pregnancy BMI, maternal education, smoking, adequacy of Prenatal Care Utilization index, previous poor pregnancy outcome, and infant sex.  Race/ethnicity (non-Hispanic black, Hispanic, non-Hispanic white, and other).  Birth records.,Texas Department of State Health Services, Center for Health Statistics | OR (95% CI), ref = 0 wells  Preterm, drilling activity:  1^st^ tertile: 1.03 (0.90, 1.18)  2^nd^ tertile: 1.03 (0.90, 1.18)  3^rd^ tertile: 1.20 (1.06, 1.37)  Preterm, production activity:  1^st^ tertile: 1.07 (0.97, 1.17)  2^nd^ tertile: 1.13 (1.02, 1.24)  3^rd^ tertile: 1.15 (1.05, 1.26)  Preterm trimester 1, production activity:  1^st^ tertile: 1.03 (0.95, 1.12)  2^nd^ tertile: 1.20 (1.09, 1.33)  3^rd^ tertile: 1.18 (1.02, 1.37)  Preterm trimester 2, production activity:  1^st^ tertile: 1.02 (0.94, 1.11)  2^nd^ tertile: 1.19 (1.08, 1.31)  3^rd^ tertile: 1.14 (0.99, 1.31)  Extreme preterm, drilling activity:  1^st^ tertile: 1.00 (0.56, 1.81)  2^nd^ tertile: 0.66 (0.35, 1.22)  3^rd^ tertile: 2.00 (1.23, 3.24)  Extreme preterm, production activity:  1^st^ tertile: 1.22 (0.86, 1.74)  2^nd^ tertile: 1.14 (0.80, 1.63)  3^rd^ tertile: 1.53 (1.03, 2.27)  Positive linear trend (tested) for drilling and production phase for all preterm, moderately preterm and extremely preterm (production phase). | We found increased odds of PTB in the third tertile of the UGD drilling OR (95% CI): 1:20 (1.06, 1.37) and UGD-production: 1:15 (1.05, 1.26) metrics. Among women in the third tertile of UGD-production, associations were strongest in trimesters one: 1:18 (1.02, 1.37), and two: 1:14 (0.99, 1.31).  The greatest risk was observed for extremely PTB (third tertile: UGD drilling: 2.00 (1.23, 3.24); UGD production: 1.53 (1.03–2.27).  We found evidence of differences in phase- and trimester-specific associations of UGD and PTB and indication of particular risk associated with extremely PTB. | |
|  | **Cross Sectional Survey Studies** | | | | | | | |
| 28 | Blinn et al., 2020 | Convenience sample of residents  US (Pennsylvania)  104  2012 - 2017 | Reported health symptoms: general; lung and heart; skin; eyes, ears, nose, and throat; gastrointestinal; nerves and muscle; reproductive; blood system; and psychological. | De-identified health assessments were reviewed retrospectively by a team of health-care providers, including a board-certified occupational-health physician and at least one nurse practitioner.  Those symptoms that could be plausibly explained by co-occurring medical conditions, medical history, or work and/or social history were excluded.  Used generalized linear modeling following Poisson distribution, used AIC to determine symptoms counts and estimated UOGD exposure, used Threshold Index Taxa Analysis (TITAN) TITAN to identify association between individual symptoms and estimated UOGD exposure. | Age, sex, smoking status, and water source  Race/ethnicity not measured  De-identified self-reported health assessments | Adjusted β Coefficient for total symptoms by exposure:  Cumulative Well Density (CWD)): 0.840, *p<*0.001  IDW: 0.015, *p<*0.001  Annual Emissions Concentrations (AEC): 5.74 x10^-6^, *p<*0.05 | When controlling for age, sex, and smoker status, each exposure estimate predicted total number of reported symptoms (CWD, p<0.001; IDW, p<0.001; AEC, p<0.05).  Two groups of symptoms (i.e., eyes, ears, nose, throat; neurological and muscular) constituted 50% of reported symptoms across exposures, suggesting these groupings of symptoms may be more likely reported by respondents when UOGD intensity increases.  10 most frequent symptoms: sore throat, headache, difficulty speaking, cough, itchy or burning eyes, stress, shortness of breath/ difficulty breathing, anxiety/worry, fatigue, sinus infection.  Our results do not confirm that UOGD was the direct cause of the reported symptoms but raise concern about the growing number of wells around residential areas. Our approach presents a novel method of quantifying exposures and relating them to reported health symptoms. | |
| 29 | Brown et al., 2019 | Self-selected individuals who completed standardized health assessment  US (PA)  104 records  2012-2017 | Presence or absence of 5 respiratory symptoms (cough, shortness of breath, sinus problems, sore throat and wheeze, any respiratory problem)    Self-report, convenience sample | Dichotomous outcomes regressed against two types of predictors: 1) direction of emissions (based on air modeling) and 2) direction of sources | Gender, age, and household water source (well water versus municipal water) | OR (95% CI) for any respiratory symptom:  North emissions: 1.00 (0.999, 1.001)  East emissions: 1.00 (0.999, 1.00)  South emissions: 1.001 (1, 1.002)  West emissions: 1.00 (0.999, 1.00)  North Sources: 0.772 (0.578, 1.03)  South Sources: 0.629 (0.428, 0.923)  West Sources: 1.508 (1.104, 2.06)  East Sources: 1.355 (0.947, 1.94)  OR (95%CI) of exposure measures and cough  North EM: 1 (0.999, 1.001)  East EM: 0.999 (0.998, 1.00)  South EM: 1.00 (1.00, 1.001)  West EM: 0.999 (0.998, 1.00)  North Sources: 0.974 (0.79, 1.201)  South Sources: 0.766 (0.575, 1.02)  West Sources: 1.232 (1.009, 1.505)  East Sources: 1.09 (0.886, 1.341)  Water Sources: 9.186 (2.403, 35.125) |  | |
| 30 | Elliott et al., 2018 | Sample of individuals living in highly fracked areas, volunteer respondents  US (Ohio)  66 households  2016 | Five categories of health symptoms were assessed in questionnaire. | Analysis of drinking water for presence of 13 unconventional oil and gas-related VOCs (e.g., benzene, disinfection by-products, gasoline-range organics, and diesel-range organics. | Age, employment status (dermal symptoms), smoking status and BMI  Race measured however only two respondents were non-white  Self-report questionnaire  Direct measurement of drinking water samples | OR (95% CI) for general symptoms related to IDW of UO&G wells within 5 km around the home:  1.52 (1.02, 2.26)  OR (95% CI) for detection as distance to nearest well increased:  Bromoform 0.28 (0.076, 1.0)  Dibromchloromethane 0.29 (0.080, 1.0)  Significant odds of detection in surface water vs. ground water for: Toluene, Bromodichloromethane, Bromoform, Chloroform, Dibromochloromethane  Spearman correlation (p value) between presence of organize compounds in water and distance to nearest UOG well:  Ground water:  Gasoline-range organics -0.40, *p=*0.008  Toluene -0.44, *p=*0.003  Surface Water:  Bromoform -0.55, *p=*0.03  Dibromochloromethane -0.48, *p=*0.03 | Those with higher inverse-distance-squared-weighted unconventional oil and gas (UOG) well counts within 5 km around the home were more likely to report experiencing general health symptoms (e.g. stress, fatigue) (OR: 1.52, 95%CI: 1.02–2.26). Odds of detection of bromoform and dibromochloromethane in surface water decreased significantly as distance to nearest UO&G well increased (OR: 0.28–0.29 per km). Similarly, distance to nearest well was significantly negatively correlated with concentrations of gasoline-range organics and toluene in ground water (rSpearman: −0.40 to −0.44) and with concentrations of bromoform and dibromochloromethane in surface water (rSpearman: −0.48 to −0.50)  This exploratory study, though limited by small sample size and self-reported health symptoms, suggests that those in closer proximity to multiple unconventional oil and gas (UOG) wells may be more likely to experience environmental health impacts. Further, presence of brominated disinfection by-products (linked to UOG wastewater) raises the question of whether UOG activities are impacting drinking water sources in the region. | |
| 31 | Johnston et al., 2021 | Residents living <1000 m from two oil wells (one active, one idle) in the Las Cienagas oil field, in California  US (CA)  972 residents  (from 488 distinct addresses)  January 2017  August 2019 | Self-reported respiratory symptom survey and forced expiratory volume during first second (FEV1), forced vital capacity (FVC) measured by trained research assistant | Logistic regression used for presence of acute respiratory symptoms (yes/no). generalized linear models used for relationship between lung function and proximity to oil wells  A random effect for household (based on address) was included to account for multiple participants from the same residential address. | Sex (male/female), age group race/ethnicity; residential distance to freeway; season baseline asthma status; ever smoker; reported indoor environmental tobacco smoke recent flu or cold symptoms & neighborhood; interactions between age and height, and age and sex.  Self-report survey | OR (95% CI) for recent wheeze:  Living near active well (vs inactive):  2.58 (1.19, 5.59)  Living within 200 m (vs farther):  1.20 (0.69, 2.13)  Reference upwind, and farther):  Downwind, near: 2.26 (1.14, 4.49)  Downwind, farther: 2.41 (1.11, 5.25)  Upwind, near: 1.71 (0.7, 4.17)  Living near active well (vs inactive):  FEV1: -111 (-213, -10.2)  FVC: -128 (-251.7, -5.0)  Reference upwind, and farther:  Downwind, near :  FEV1: -236.4 (-424.9, -48.0)  FVC: -296.0 (-524.8, -67.2)  Downwind, farther;  FEV1: -206.9 (-313.9, -99.8)  FVC: -253.4 (-383.8, -123.1)  Upwind, near:  FEV1: -195.9 (-314.9, -76.7)  FVC: -228 (-373, -83.1)  Residents living downwind and less than 200 m from oil operations have, on average, −414 mL lower FEV1 (95% CI: −636, −191) and −400 mL lower FVC (95% CI: −652, −147) compared to residents living upwind and more than 200 m from the wells. | Among 747 valid spirometry tests, we observe that living near (less than 200 m) oil operations was associated with, on average, −112 mL lower FEV1 (95% CI: −213, -10) and −128 mL lower FVC (95% CI: −252, −5) compared to residents living more than 200 m from the sites after adjustments for covariates, including age, sex, height, proximity to freeway, asthma status and smoking status. When accounting for predominant wind direction and proximity, we observe that residents living downwind and less than 200 m from oil operations have, on average, −414 mL lower FEV1 (95% CI: −636, −191) and −400 mL lower FVC (95% CI: −652, −147) compared to residents living upwind and more than 200 m from the wells.  Living nearby and downwind of urban OGD sites is associated with lower lung function among residents, which may contribute to environmental health disparities. | |
| 32 | Mayer et al., 2021 | Sample of individuals surveyed in 3 communities: no UNGO; a lot of UNGO; permitted wells but with little active drilling.  US (Colorado)  890  2015 - 2016 | Cross-sectional, ecologic survey.  Self-rated health with and without the inclusion of self-reported stress caused by UNGO activity in the model. | Ordinal logistic regression. Estimated two models and used Akaike Information Criterion and Bayesian Information Criteria to compare model fit. | Age, sex, income, race, education, trust in regulators.  Self-report survey | OR (95% CI) for reporting satisfied with health (ref = town with high UNGO activity):  No UNGO (Fort Collins): 1.81 (1.16, 2.83)  Permitted UNGO with few active wells (Windsor): 1.88 (1.19, 2.97)  2^nd^ model includes effect of self-reporting a belief that UNGO causes stress:  No UNGO (Fort Collins): 1.87 (1.19, 2.95)  Permitted UNGO with few active wells (Windsor): 2.05 (1.28, 3.27) | Results from ordinal logistic regression models suggest that living in a community that hosts extraction is associated with lower self-rated health and that self-reported stress from local oil and gas operations is associated with lower self-rated health. To some degree, trust in regulators seems to improve self-rated health. | |
| 33 | McKenzie et al., 2019b | Sample of non-smoking adults living in Northeastern Colorado  US (Colorado)  97  2015 - 2016 | Augmentation index (a measure of arterial stiffness as a marker of cardiovascular risk), systolic + diastolic blood pressure, lab testing for 3 interleukin (IL) factors and tumour necrosis factor (TNF)  In-person visits where participants completed questionnaire, underwent biometric testing and collection of blood samples. | Log-10-transformed the systemic inflammation concentrations prior to statistical analysis. The augmentation index, systolic blood pressure, and disinfection by-products measurements met all assumptions for linear regression and were not transformed.  Used separate linear mixed models with random intercepts for  each participant to evaluate the association between each health measurement (augmentation index, systolic blood pressure, disinfection by-products, IL-1β, IL-6, IL-8 and TNF –α) and categorized intensity of O&G well activity (intensity-adjusted inverse distance weighted measure - IA-IDW) within 16 km of each participant's home (low (reference) medium & high). | Age, sex, race/ethnicity, BMI, education, income and employment status  Self-report questionnaire | OR of mean difference in cardiovascular marker by intensity-adjusted IDW (ref = 1^st^ tertile)  Augmentation index (% at heart rate 75):  Med: 5.1 (−0.1, 10.4)  High: 6.0 (0.6, 11.4)  Systolic blood pressure for those NOT taking Rx:  Med: 1 (-6, 8)  High: 6 (0.1, 13)  IL-1β adjusted means:  Med: 0.557 (0.481, 0.642)  High: 0.610 (0.527, 0.701)  TNF-α.for those NOT taking Rx:  Med: 4.82 (3.99, 5.78)  High: 5.03 (4.15, 6.04) | Adjusted mean augmentation index differed by 6.0% (95% CI: 0.6, 11.4) and 5.1% (95% CI: −0.1, 10.4) between high and medium, respectively, and low exposure tertiles.  The greatest mean IL-1β, and α-TNF plasma concentrations were observed for participants in the highest exposure tertile.  IL-6 and IL-8 results were consistent with a null result.  For participants not taking prescription medications, the adjusted mean systolic blood pressure differed by 6 and 1 mm Hg (95% CIs: 0.1, 13 mm Hg and −6, 8 mm Hg) between the high and medium, respectively, and low exposure tertiles.  For participants taking prescription medications, systolic blood pressure and disinfection by-products results were consistent with a null result.  Results support associations between O&G activity and augmentation index, systolic blood pressure, disinfection by-products, IL-1β, and TNF-α. | |
| 34 | Rabinowitz et al., 2015 | Random selection of 20 geographic points from 38 contiguous townships in the study county with a ground-fed well water supply  US (Pennsylvania)  492  2013 | Self-report of upper and lower respiratory, dermal, neurological, cardiovascular, and gastrointestinal symptoms.  Survey administered by trained personnel. | Used generalized linear mixed model analysis (analysis used maximum likelihood estimation with adaptive quadrature methods including a random effect for household to account for the clustering of individuals within a household. | Age, sex, education, smoker present in household, awareness of environmental hazard nearby, employment type, whether animals were present in the home or backyard, and awareness of environmental risk.  Self-report survey | Mean (SD) total health symptoms per individual by proximity to nearest gas well:  <1 km: 3.27 +/- 3.72  1-2 km: 2.56 +/- 3.26  >2 km: 1.60 +/- 2.14  OR (95% CI), ref = >2 km  Reported skin conditions:  <1 km: 4.13 (1.38, 12.3)  1-2 km: 1.44 (0.42, 4.9)  Reported upper respiratory symptoms:  <1 km: 3.10 (1.45, 6.65)  1-2 km: 1.76 (0.81, 3.76)  No difference for other respiratory, neurological, cardiovascular or gastrointestinal symptoms. | The number of reported health symptoms per person was higher among residents living < 1 km (mean ± SD, 3.27 ± 3.72) compared with > 2 km from the nearest gas well (mean ± SD, 1.60 ± 2.14; p = 0.0002).  In a model that adjusted for age, sex, household education, smoking, awareness of environmental risk, work type, and animals in house, reported skin conditions were more common in households < 1 km compared with > 2 km from the nearest gas well (OR = 4.1; 95% CI: 1.4, 12.3; p = 0.01).  Upper respiratory symptoms were also more frequently reported in persons living in households < 1 km from gas wells (39%) compared with households 1–2 km or > 2 km from the nearest well (31 and 18%, respectively) (p = 0.004). No equivalent correlation was found between well proximity and other reported groups of respiratory, neurological, cardiovascular, or gastrointestinal conditions.  Although these results should be viewed as hypothesis generating, and the population studied was limited to households with a ground-fed water supply, proximity of natural gas wells may be associated with the prevalence of health symptoms including dermal and respiratory conditions in residents living near natural gas extraction activities. | |
| 35 | Steinzor et al., 2013 | Convenience sample of residents living in non-urban communities in areas with shale gas development.  US (Pennsylvania)  108  2011 - 2012 | Health symptoms and outcomes related to 13 health categories.  Self-report through health survey. | Community-based participatory research, surveyed participants living in “gas patches” about health symptoms. Testing conducted at the homes of a subset of respondents – 34 air tests and 9 water tests in 35 households for VOCs/benzene, toluene, ethylbenzene and xylene, and alpha/beta radiation, radon, and radium.  Bivariate tests of association between symptom prevalence and nearest facility within 1500 ft.(vs more than 1500 ft) and descriptive stats of match between known health effects of chemicals and symptoms reported by county | Age, sex, smoking status, occupation.  Self-report survey | Significantly higher rates of following symptoms among those with residence within 1500 feet vs more than 1500 feet.  p < 0.001: Throat irritation, frequent nose bleeds  *p<*0.01: sinus problems, eye burning, skin rashes, loss of sense of smell, persistent cough  p>0.05: nasal irritation, severe headaches, swollen painful joints  Health symptom reporting by the individual living in a home where testing occurred matched the know health effects of chemicals detected at an overall rate of 68% (range 33-100%).  Youngest respondents had highest reporting rate of nosebleeds and severe headaches.  More than half water samples contained methane. | Many symptoms showed a clearly identifiable pattern: as the  distance from facilities increases, the percentage of respondents reporting the symptoms generally decreases. For example, when a gas well, compressor station, and/or impoundment pit were 1500-4000 feet away, 27 percent of participants reported throat irritation; this increased to 63 percent at 501-1500 feet and to 74 percent at less than 500 feet. At the farther distance, 37 percent reported sinus problems; this increased to 53 percent at the middle distance and 70 percent at the shortest distance. Severe headaches were reported by 30 percent of respondents at the farther distance, but by about 60 percent at the middle and short distances.  In sum, while these data do not prove that living closer to oil and gas facilities causes health problems, they do suggest a strong association since symptoms are more prevalent in those living closer to facilities than those living further away. | |
| 36 | Tustin et al., 2017 | Adults in the Geisinger clinic health system living in Pennsylvania who responded to 23,700 mailed self-administered surveys  US (Pennsylvania)  7,785  2014 | Population-based survey of self-reported health symptoms of adult patients with chronic rhinosinusitis, migraine headache and higher levels of fatigue.  Case defined as having one or more of the three primary outcomes  Controls defined as those having no or minimal disease.  Self-reported symptom survey | Used weighted logistic regression to evaluate associations between UNGD activity and symptoms while adjusting for confounding variables. | Sex, race/ethnicity, age, receipt of medical assistance, smoking status, Charlson comorbidity index, residential place type obtained from electronic health record, education status, marital status, household income, hay fever, nasal polyps, age at onset of nasal/sinus symptoms, history of sinus surgery and current use of sinusitis medications from survey self-report  Community socioeconomic deprivation in townships boroughs and cities linked to census tract info  Self-report symptom survey; electronic health records (Geisinger Health System); US census data | OR (95% CI) (ref = 1^st^ quartile IDW):  Current chronic rhinosinusitis plus migraine:  2^nd^ quartile: 0.82 (0.43, 1.57)  3^rd^ quartile: 0.74 (0.38, 1.47)  4^th^ quartile: 1.49 (0.78, 2.85)  Current chronic rhinosinusitis plus higher levels of fatigue:  2^nd^ quartile: 1.06 (0.62, 1.80)  3^rd^ quartile: 0.94 (0.53, 1.66)  4^th^ quartile: 1.88 (1.08, 3.25)  Migraine plus higher levels of fatigue:  2^nd^ quartile: 1.06 (0.63, 1.78)  3^rd^ quartile: 0.80 (0.49, 1.31)  4^th^ quartile: 1.95 (1.18, 3.21)  All 3 outcomes:  2^nd^ quartile: 1.05 (0.63, 1.78)  3^rd^ quartile: 0.73 (0.42, 1.27)  4^th^ quartile: 1.84 (1.08, 3.14)  Significant OR for models stratified by symptom onset after 2006 for 2^nd^ and fourth quartile for chronic rhinosinusitus. | The response rate was 33%.  Of 7,785 study participants, 1,850 (24%) had current chronic rhinosinusitus symptoms, 1,765 (23%) had migraine headache, and 1,930 (25%) had higher levels of fatigue.  Among individuals who met criteria for two or more outcomes, adjusted OR for the highest quartile of UNGD activity compared to the lowest were [OR (95% CI)] 1.49 (0.78, 2.85) for chronic rhinosinusitis plus migraine, 1.88 (1.08, 3.25) for chronic rhinosinusitus plus fatigue, 1.95 (1.18, 3.21) for migraine plus fatigue, and 1.84 (1.08, 3.14) for all three outcomes together.  Significant associations were also present in some models of single outcomes.  This study provides evidence that UNGD is associated with nasal and sinus, migraine headache, and fatigue symptoms in a general population representative sample.  Significant associations were also present in some models of single outcomes.  Absence of significant effect associated with 2^nd^ and 3^rd^ quartile exposure groups suggests threshold effect for the outcomes examined. | |
|  | ***Ecological Studies*** | | | | | | |  |
| 37 | Apergis et al., 2021 | Individuals in 76 counties  US (Oklahoma)  590,780  1998 - 2017 | i) life expectancy  ii) mortality rate  iii) number of deaths from all types of cancer /100,000  iv) number of deaths from cardiac failures /100,000  v) number of deaths from respiratory disease/100,000.  Institute for Health Metrics and Evaluation (IHME) at the University of Washington (life expectancy, mortality); Institute for Health Metrics and Evaluation (IHME) at the University of Washington; America's Health Rankings analysis of CDC WONDER Online Database (Cardiac); National Center for Health Statistics (from the US Center for Disease Control and Prevention (respiratory) | Panel time-series approach using employ Common Correlated Effects (CCE) estimator to estimate the long-run relationship between the health profile and its determinants | County-level:  (i) annual non-fracking (conventional) activity (ii) economic factors, (iii) metro or non-metro area  (iv) average age (v) % adults who smoke cigarettes / year (vi) % of non-white (vii) a variable that explicitly considers the year 2006 where unconventional activities became widespread in Oklahoma (viii) electricity grid access (ix) transportation infrastructure (x) the air pollution levels originating from the manufacturing sector xi) % of people with a high school degree or higher    Institute for Health Metrics and Evaluation (IHME) at the University of Washington; Oil and Gas Division of the Oklahoma Corporation Commission ;Bureau of Economic Analysis (BEA) database; Bureau of Labour Statistics; American Community Survey (ACS) database provided by the US Census Bureau; Kaiser Family Foundation analysis of the Centers for Disease Control and Prevention (CDC)'s Behavioral Risk Factor Surveillance System (BRFSS); U.S. Department of Energy’s Energy Information Administration; Centre of Budget and Policy Priorities; US Environmental Protection Agency | Common correlated effects mean group (CCE-MG) estimates and p values: 1998–2017  Log(fracking) common correlated mean group estimates):  Life expectancy: −0.041 p ≤ 0.01  Mortality rates:  All cause; 0.064 p ≤ 0.01  Cancer; 0.075 p ≤ 0.01  Heart diseases ; p ≤ 0.01[0.00]  Respiratory diseases; p ≤ 0.01 | On average, a 1% increase in the number of fracking wells leads to a 4.2% reduction in life expectancy. Similar trends are observed with the remaining health indicators, where a 1% increase in the number of fracking wells leads to a 6.8% increase in the mortality rate, a 7.9% increase in cancer diseases, a 7.3% increase in cardiac diseases, and, finally, a 5.9% increase in respiratory diseases.  These findings provide evidence that UNGD activities pose significant risks to the public health profile across the Oklahoma population. Such findings are expected to have substantial implications for the national debate on the regulation of UNGD | |
| 38 | Busby and Mangano, 2017 | Newborns in 67 counties, 2 time periods  US (Pennsylvania)  100,680  2003 - 2010 | County-level analysis – 10 most fracked counties in the Northeast and Northwest respectively compared to 57 counties in Pennsylvania in before/after design  Perinatal outcomes – early infant mortality (0-28 days)  Commonwealth of Pennsylvania, Department of Health for yrs 1999-2014 | Calculated the RR of early infant mortality rates (per 1000 live births) between the non-fracked and fracked periods as: RR = early infant mortality (2007-2010)/early infant mortality (2003-2006) for each county, for the entire State of Pennsylvania and for the aggregate of all Pennsylvania counties less the 10 most fracked counties.& mapped the result RR to compare this with a map of fracking well density by county  Commonwealth of Pennsylvania | No covariate adjustment. | RR (95% CI) early infant mortality in period before and after fracking:  Northeast counties: 1.67 (1.10, 2.51)  Southwest counties: 1.18 (0.95, 1.46)  All 10 fracked counties vs rest of Pennsylvania: 1.29 (1.05, 1.55)  Increased risk associated with exposure to groundwater:  RR (95% CI) All North East States with heavy fracking: 1.67 (1.10, 2.51), *p=*0.014  All 10 fracked states: 1.29 (1.05, 1.55), *p=*0.011  Significant association of well water exposure and RR for early infant mortality in the North East group but not the South West group. | Whilst early infant deaths decreased by 2.4% in the State over the period, in the 82,558 births in the 10 fracked counties there was a significant increase in mortality (238 vs 193; RR = 1.29; 95% CI 1.05, 1.55; p = 0.011). For the five north east fracked counties Bradford, Susquehanna, Lycoming, Wyoming and Tioga the combined early infant mortality increased from 34 deaths to 60 (RR 1.66; 1.05, 2.51; p = 0.014), whereas in the south western 5 counties Washington, Westmoreland, Fayette, Butler and Greene the increase was modest, 157 to 178 (RR 1.18; 0.95, 1.46; p = 0.13). Increased risk was associated with exposure to groundwater, expressed as the county ratio of water wells divided by the number of births.  Fracking appears to be associated with early infant mortality in populations living in counties where the process is carried out. There is some evidence that the effect is associated with private water well density and/or environmental law violations.  Fracking appears to be associated with early infant mortality, there is some evidence that this effect is associated with private well water density and/or environmental law violations | |
| 39 | Bushong et al., 2022 | All counties reporting asthma hospital admission rates (62 counties)  US (Pennsylvania)  Population not specified  2001 - 2014 | Age-adjusted hospital admission rates for asthma  The Pennsylvania Department of Health (PADOH) | Two multiple linear regression models with fixed year effects - one for urban and rural counties and the other restricted to rural counties  hospital admission rates were log transformed | Median income, % urban population, annual average PM_2.5_ emissions, county smoking, % white population, % people over 25 yrs rural or urban based on population density above or below the population density of the whole state and Census data.  Small Area Health Insurance estimates, American Community Survey (ACS) five-year estimates for 2005-2009 and 2010-2014, 2010 Census Urban and Rural Classifications. Centers of Disease Control and Prevention. | Associated % change in asthma hospital admission rates per 0.01 wells/square mile increase (95% CI) for:  62/67 counties reporting asthma hospital admission rates:  +3.0% (0.95%, 7.21%)  Rural counties only:  +3.31% (1.14%, 7.70%) | We found a significant positive association between the asthma hospital admission rates and annual well density for all the counties in the state (3% increase in hospital admission rates attributable to hydraulic fracturing, *p<*0.001). For a sensitivity analysis, we excluded urban counties (urban counties have higher asthma exacerbations) and focused on rural counties for the yrs 2005–2014 and found a significant association (3.31% increase in hospital admission rates attributable to hydraulic fracturing in rural counties, *p<*0.001)  An even stronger association was found between asthma hospitalization admission rates (HAR) and PM2.5 levels (7.52% increase in HAR attributable to PM2.5, p<0.001). As expected, asthma HAR was significantly higher in urban compared to rural counties and showed a significant racial disparity. We conclude that publicly available data at the county-level supports an association between an increase in asthma HAR and UNGD in rural counties in Pennsylvania | |
| 40 | Denham et al., 2019 | 2 X 12-year panels:67 counties (panel 1) and 54 non-metropolitan counties ( panel 2)  US (Pennsylvania)  Population not specified  2003 - 2014 | Total hospitalizations, and within one of 16 major health categories (determined by International Classification of Disease ICD-9 grouping)  Inpatient discharge records from the Pennsylvania Health Care Cost Containment Council, all included | County level analysis –, 2 X 12-year panels, 67 Pennsylvania counties and 54 counties that are not large metropolitan  Multivariate regressions with county and year fixed effects to examine the association of exposure measures (well count, cumulative well density, & contemporaneous wells) and hospitalization | County demographics distribution for sex, race, age group, % in poverty, % unemployed, median income, total number of hospital beds and proportion of uninsured  Population count estimates from the Surveillance, Epidemiology, and End Results (SEER) program; small Area Income and Poverty Estimates (SAIPE); Bureau of Labor Statistics. Area Health Resource File (HRSA, 2021); | Positive adjusted association between cumulative well density for genitourinary hospital admission rates.  β Coefficient (95% CI) for all counties:  Kidney infections: 6.19 (2.20, 14.68)  Renal calculus: 7.91 (2.63,13 .20)  Urinary tract infections 7.16 (2.52, 11.80)  β Coefficient (95% CI) for non-metropolitan counties for same outcomes:  Kidney infections: 8.44 (2.20, 14.68)  Renal calculus: 8.21 (1.82, 14.61)  Urinary tract infections: 9.70 (4.22, 15.19)  Skin-related hospitalization rates also associated with cumulative well count and density. Coefficient.: 0.008 p = 0.007, (95% CI: 0.002, 0.01 [hospitalizations/10,000])  Unconventional gas wells drilled in a year not significantly associated with any outcomes. | After correcting for multiple comparisons, we found a positive association of cumulative well density (per km2) with genitourinary hospitalization rates.  When large metropolitan counties were excluded, this relationship persisted, and positive associations of skin-related hospitalization rates with cumulative well count and well density were observed. The association with genitourinary hospitalization rates is driven by females in 20-64 yrs group, particularly for kidney infections, calculus of ureter, and urinary tract infection.  Contemporaneous wells drilled were not significantly associated with hospitalizations after adjustment for multiple comparisons.  Our study shows that long-term exposure to unconventional gas development may have an impact on prevalence of hospitalizations for certain diseases in the affected populations and identifies areas of future research on unconventional gas development and health. | |
| 41 | Denham et al., 2021 | Hospital admissions for myocardial infarct comparing counties in Pennsylvania (47 counties) vs New York (24 counties)  US (Pennsylvania & New York)  Population not specified  2005-2014 | Hospital admissions with principal diagnosis of AMI and decedents with AMI as the cause of death, groups into age/ sex categories  Population estimates are from the Surveillance, Epidemiology, and End Results (SEER) program. | County level analysis n=2840 county year quarters  Longitudinal panel analysis, with time-varying country characteristics, model includes quarter-year fixed effects, which account for time trends in the outcomes, and county fixed effects, which adjust for county-specific time-invariant characteristics. Additionally, adjusted for state-specific time trends by including a state-by-year interaction. | Adjustment for county level age, sex, racial and ethnic composition, hospitalization rates, economic and health care factors: unemployment rate, poverty rate, uninsured rates for younger adults (45-54, 55- 64), sex-specific rates for adults 40 – 64 yrs, median household income, and the total number of hospitals.  Surveillance, Epidemiology, and End Results (SEER) program; Small Area Income and Poverty Estimates (SAIPE); Bureau of Labor Statistics. Area Health Resource File (HRSA, 2021); Small Area Health Insurance Estimates (SAHIE) Program. | Between 2005 and 2014, average county-year-quarter AMI hospitalization and AMI mortality rates exhibited decreasing trends, in both states, with consistently higher rates in Pennsylvania  Unadjusted rate of hospitalization (95% CI) associated with county-level cumulative well counts (100 wells):  Females:  65-74 yrs: 0.47 (0.18, 0.77), *p=*0.002  75+ yrs: 1.11 (0.39, 1.82), *p=*0.003  Males:  45-54 yrs: 0.26 (0.07,0.46), *p=*0.009  65-75 yrs: 0.40 (0.09, 0.71), *p=*0.011  Cumulative well density, per square mile:  Females:  75+ yrs: 9.67 (1.92, 17.42), *p=*0.015  Males:  45-54 yrs: 2.63, (0.67, 4.59), *p=*0.009  Unadjusted rate of mortality:  Males:  45-54 yrs: 0.09 (0.01, 0.17), *p=*0.021  NO evidence of contemporaneous wells and AMI hospitalizations.  . | A hundred cumulative wells is associated with: 0.26 more hospitalizations per 10,000 males 45-54 yrs (95% CI 0.07,0.46),  0.40 more hospitalizations per 10,000 males 65-74 yrs (95% CI 0.09,0.71),  0.47 more hospitalizations per 10,000 females 65-74 yrs (95% CI 0.18, 0.77) and 1.11 more hospitalizations per 10,000 females 75+ yrs (95% CI 0.39, 1.82), translating into 1.4–2.8% increases.  One additional well per square mile is associated with 2.63 more hospitalizations per 10,000 males 45-54 yrs (95% CI 0.67, 4.59) and 9.7 hospitalizations per 10,000 females 75+ yrs (95% CI 1.92, 17.42), 25.8% and 24.2% increases, respectively.  A hundred cumulative wells is associated with an increase of 0.09 deaths per 10,000 males 4554 yrs (95% CI 0.02, 0.16), a 5.3% increase.  Cumulative UNGD is associated with increased AMI hospitalization rates among middle-aged men, older men and older women as well as with increased AMI mortality among middle-aged men.  Our findings lend support for increased awareness about cardiovascular risks of UNGD and scaled-up AMI prevention as well as suggest that bans on hydraulic fracturing can be protective for public health. | |
| 42 | Erickson et al., 2022 | Maternal infant pairs in 5 counties  US (Colorado)  252,505  1999 - 2019 | Birthweight, prematurity defined as a live birth delivered before 37 weeks  Colorado Dept of Public Health & Environment’s Vital Birth Statistics | County level analysis  Generalized linear models used to model annual county well density and production on birthweight and prematurity, birthweight outcome used Gaussian distribution and adjusted for prematurity; prematurity model used Poisson distribution. | Adjustment for county level: population, population density; age; sex; race; education; household income; percentage of population at poverty level matched to closest census year of birth.  US Census data | Birthweight model parameter estimate:  Well density: 186.9 grams, *p*=0  Well production: 14.3 grams, *p*=4.36 E-.09)  Well density*production: -9.3 grams, *p*=0  Prematurity parameter estimate: Well density:0.0241, *p*=0.30  Well production: 0.0162, *p*=5.8E-07)  Well density*production: -0.0014, *p*=0.24 | Our modeling approach showed an interesting effect where hydraulic fracturing exposure metrics have a mixed effect directional response. This effect was detected on birth weight when well density, production and their interaction are accounted for. The interaction effect provides an additional interpretation to discrepancies reported previously in the literature. Our approach only detected a positive association to prematurity with increased production.  Our findings demonstrate two main points: First, the effect of hydraulic fracturing is detectable by using countywide unidentified data. Second, the effect of hydraulic fracturing can be complicated by the number of operations and the intensity of the activities in the area. | |
| 43 | Finkel, 2016 | Residents in 6 counties  US (Pennsylvania)  1,031,953  Time periods:  2000 – 2004  (before fracking onset)  2004 - 2008 (commencement of fracking)  2008 – 2012 (most fracking activity and all PA counties) | Cancer incidence (thyroid, bladder, and leukemia) and mortality  Pennsylvania Department of Health (DOH) Bureau of Health Statistics and Research's Pennsylvania Cancer Registry (PCR).25 | County level analysis, 2 with highest, two with little/no and two with moderate UGD activity.  Pennsylvania Cancer Registry generated SIRs for leukemia, thyroid and bladder cancer based on strong relationship of these cancers to environmental pollutants by age and sex. | Age, sex stratified, no county level adjustments for covariate.  Pennsylvania Department of Health (DOH) Bureau of Health Statistics and Research's Pennsylvania Cancer Registry (PCR). | Urinary bladder cancers SIR (95% CI) in highly fracked counties:  Males:  Washington:  2000-2004 115.2 (14.5, 130.8)  2004-2008 131.6 (15.3, 148.4)  2008-2012 128.6 (14.7, 145.7)  Westmoreland,  2000-2004 114.0 (10.5, 135.3)  2004-2008 131.7 (126, 137.4)  2008-2012 127.3 (116.6, 138)  Urinary bladder cancers SIR (95% CI) in highly fracked counties:  Females:  Washington:  2000-2004 122.9 (98.1, 147.7)  2004-2008 135.0 (108.5, 161.5)  2008-2012 130.7 (104.8, 156.6)  Westmoreland:  2000-2004 101.4 (84.8,118)  2004-2008 98.4 (81.9, 114.9)  2008-2012 147.1 (127.1, 167.2) | The number of urinary bladder cancer cases higher was than expected among both men and women in highly fracked counties. This was already high before onset of fracking and increased over time. In counties with the fewest number of producing wells, the increase was essentially non-existent.  The number of observed cases of thyroid cancer increased substantially among both sexes over the time period in all counties regardless of the number of wells drilled. The pattern for leukaemia was mixed among males and females and among the counties regardless of the extent of shale gas development activities.  Potential risk factors other than shale gas development must be taken into account to explain the higher than expected cancer cases in counties with and without shale gas wells before and during unconventional shale gas activity. | |
| 44 | Fryzek et al., 2013 | Children living in all 67 counties  US (Pennsylvania)  67 counties  Population not specified  1990 - 2009 | All childhood cancers, leukemia, and central nervous system tumors incidence  Pennsylvania Department of Health (DOH) Bureau of Health Statistics and Research's Pennsylvania Cancer Registry | County level analysis.  SIRs were calculated to compare the observed number of cancers by county with that expected on the basis of rates of cancer in the general population for childhood cancers, childhood leukemia, and central nervous system tumors 1990 to 2009, stratified by 5-year age groups, race and sex. Indirectly standardized. | Age, sex and race  Pennsylvania Department of Health (DOH) Bureau of Health Statistics and Research's Pennsylvania Cancer Registry | SIR (95% CI) childhood central nervous system tumours:  Before drilling:  0 wells = ref  1-500 wells = 0.90 (0.79–1.02)  501 – 1,000 wells = 0.93 (0.58–1.41)  1,001-2,000 wells = 0.82 (0.53–1.22)  >2,001 wells = 0.83 (0.59–1.14)  Total for all counties with wells = 0.89 (0.79–0.99)  After drilling:  0 wells = ref  1-500 wells = 1.22 (1.07–1.37)  501 – 1,000 wells = 0.81 (0.52–1.20)  1,001-2,000 wells = 0.88 (0.61–1.21)  >2,001 wells = 1.15 (0.90–1.44)  Total for all counties with wells = 1.13 (1.02–1.25) | The total number of childhood cancers observed was close to expected both before drilling began (SIR = 0.94; 95% CI, 0.90 to 0.99) and after drilling (SIR = 1.02; 95% CI, 0.98 to 1.07) for counties with oil and natural gas wells.  Analyses for childhood leukemia were also unremarkable (SIR for leukemia before drilling = 0.97 [95% CI, 0.88 to 1.06]; SIR for leukemia after drilling = 1.01 [95% CI, 0.92 to 1.11]).  A slightly elevated SIR was found for central nervous system tumors after drilling (SIR = 1.13; 95% CI, 1.02 to 1.25). This was because of a slight excess in those counties with the fewest number of wells.  This study offers comfort concerning health effects of UOGD on childhood cancers. | |
| 45 | Hu et al., 2022 | US Population living in states where there is fracking  US (49 states with fracking activity)  2010 - 2018 | Mortality from stroke  Centres for Disease Control and Prevention | Geographical and temporal weighted regression with fracking annualized loss expectancy (ALE) as the modelled fracking exposure variable and after Akaike Information Criterion testing of 3 models. Used variance inflation factor (VIF) to test for collinearity and if greater than 10, variables were removed | Disease comorbidity (diabetes, cardiovascular, overdose); behavioural risk factors (tobacco use, high cholesterol, PAI; socioeconomic risk factors (mean income, marital rate, employment rate).  Centres for Disease Control and Prevention | Average effect of fracking in geographical and temporal weighted regression (GTWR) model for 65+ stroke mortality:  Minimum: -0.327  LQ : 0.041  Med: 0.116  UQ: 0.154  Maximum: 0.394  Average: 0.094 | Fracking modelled exposure variable (ALE) is moderately correlated with stroke mortality at ages over 65 in most states of fracking, in addition to cardiovascular disease and drug overdose being positively correlated with stroke mortality. Furthermore, the correlations between fracking ALE and stroke mortality in men appear to be higher than in women near the Marcellus Shale, including Ohio, Pennsylvania, West Virginia, and Virginia, while stroke mortality among women is concentrated in the Great Plains, including Montana, Wyoming, New Mexico, and Oklahoma. Lastly, within two kilometers of the fracking mining activity, the level of benzene in the air was found to be significantly correlated with the fracking activity in Colorado. | |
| 46 | Jemielita et al., 2015 | Inpatient discharges in 3 counties (67 zip codes): 2 with UOGD activity and 1 with no UOGD activity  US (Pennsylvania)  Population 157,526  2007 - 2011 | Inpatient prevalence rate of hospital admission for 25 different health categories and overall inpatient prevalence rates.  Pennsylvania Health Care Cost Containment Council. | Zip code level analysis, for 3 counties with large increases in active wells over time period studied.  Association of inpatient prevalence rates with number of wells per zip code and, separately, with wells per km2 (separated into quantiles and defined as well density) were estimated using fixed effects Poisson models. To account for multiple comparisons, a Bonferroni correction with associations of *p<*0.00096 was considered statistically significant. | Age, sex, insurance coverage, year of inpatient record.  The Nielsen Company statistics for every zip code in the US. Nielsen bases their estimates on products of the United States Census Bureau, including the 2010 Census Summary File 1.  Pennsylvania Health Care Cost Containment Council. | RR for number of wells/zip code/year:  Cardiology: 1.0007, *p=*0.0007  Dermatology: 1.0010, *p=*0.039  Neurology: 1.0006, *p=*0.037  Oncology: 1.0015, *p=*0.004  Urology: 1.0010, *p=*0.012  RR for wells/km^2^ and cardiology inpatient prevalence (ref = 0 wells/km^2^)  1^st^ quantile: 1.021, *p=*0.667  2^nd^ quantile: 1.142, *p=*0.018  3^rd^ quantile: 1.27, *p=*0.001  Neurology:  1^st^ quantile: 0.922, *p=*0.344  2^nd^ quantile: 1.157, *p=*0.048  3^rd^ quantile 1.188, *p=*0.062  RR for number of wells per zip code per year:  Cardiology, *p=*0.0007  Neurology, *p<*0.00096 | Cardiology inpatient prevalence rates were significantly associated with number of wells per zip code (*p<*0.00096) and wells per km^2^ (*p<*0.00096).  Neurology inpatient prevalence rates were significantly associated with wells per km^2^ (*p<*0.00096).  Furthermore, evidence also supported an association between well density and inpatient prevalence rates for the medical categories of dermatology, neurology, oncology, and urology.  These data suggest that UGOD wells, which dramatically increased in the past decade, were associated with increased inpatient prevalence rates within specific medical categories in Pennsylvania. | |
| 47 | Ma et al., 2016 | Newborns  US (Pennsylvania)  1,401,813  2003 - 2012 | Birth defect prevalence rate (total, structural and functional/ developmental)  Pennsylvania Birth Certificate | Zip code level analysis.  A segmented regression analysis of interrupted time-series method used to estimate the changes in the level and trend in the birth defects prevalence rate while controlling for maternal characteristics. The binary outcome of birth defects (Yes/No) was the dependent variable and secular trend without UNGD, post-UNGD level, post-UNGD trend, area with/ without UNGD, unconventional well density and maternal characteristics were the independent variables in the final model. | Maternal smoking status, highest educations level, self-designated race, age at delivery, pre-pregnancy BMI, primary payer for delivery, mother on WIC during pregnancy, diabetes, hypertension (pre- or during pregnancy), infection during pregnancy.  Mather’s self-designated race: American Indian or Alaska Native/ Asian/Pacific islander/ Black/ Other/ White.  Pennsylvania Birth Certificate. | Area with/ without UNGD associated with any birth defects OR (95% CI)  Yes (ref = no): 1.22 (1.13, 1.32)  Secular trend without UNGD: 0.99 (0.99, 0.99)  Post-UNGD level: 0.97 (0.81, 1.17)  Post-UNGD trend (per year): 1.00 (0.99, 1.00)  Unconventional (well density per square kilometer): 0.93 (0.85, 1.01)  Similar pattern for structural and functional/developmental birth defects outcomes. | Among areas with UNGD, birth defects prevalence rate was 6.3/1,000 live births before UNGD and 5.0/1,000 live births after UNGD, a 20.6% drop with *p<*0.01, while birth defects prevalence rate in zip code areas without UNGD was 4.7/1,000 live births.  After controlling for maternal characteristics and secular trend, the adjusted OR for annual post UNGD birth defects trend did not change (OR=1.00, *p=*0.29) and post-UNGD birth defects level decreased but was not statistically significant (OR =0.97, *p=*0.12).  The OR for unconventional well number per square kilometer was 0.93, *p=*0.10.  We conclude UNGD was not associated with birth defects prevalence rate trend and level changes. Further studies are needed to address why birth defects prevalence rate in UNGD areas were consistently 22% higher than in non-UNGD areas. | |
| 48 | Makati et al., 2022 | US (VA)  76  1990 – 2019 | Chart Review    West Virginia University Health System | Prevalence rates of all ANCA associated vasculitis, PR3-ANCA and MPO-ANCA before and after increase in natural gas drilling sites | None | Prevalence Rates/1,000,000 people before and after 2010 for:  Overall ANCA associated vasculitis: 64.8 (n=21); 141.9 (n-55), p = 0.0001  PR3-ANCA: 41.9 (m=10); 42 (n=13), p = 1.00  MPO-ANCA: 42(n=8); 101.7 (m=39), p = 0.028 | The increase in prevalence of AAV was primarily due to an increase in MPO-AAV (43 vs 101.7 cases per million before and after 2010, respectively, p = 0.028). During this time, the production of natural gas through fracking increased, rising more than tenfold after 2010 (p-value < 0.001). Heat mapping reveals that the increase in cases of AAV occurred in areas of increased fracking activity.  There was an increase in the prevalence of patients who were newly diagnosed with AAV over time in north central West Virginia. Further studies are required to ascertain the potential role of environmental exposure in the pathophysiology of AAV. | |
| 49 | Peng et al., 2018 | Residents aged >5 yrs in 39 counties with unconventional wells drilled & 28 counties without drilling  US (Pennsylvania)  67 counties  2001 - 2013 | Hospitalizations for pollution sensitive conditions - AMI, upper respiratory infection, asthma, pneumonia and chronic obstructive pulmonary disease.  Pennsylvania Health Care Cost Containment Council's compilation of all inpatient hospital admission records database. | County level analysis.  Difference in difference panel design, with clustering of within-county standard errors clustered at county level, adjusting p values for multiple comparisons. | County-level characteristic for average age, the share of different types of insurance, the share of male patients, the share of different race and ethnicity groups, the share of different types of admission, average Charlson index, unemployment rate, poverty rate, annual quartiles of median household income, log of population density, log of annual coal production, log of number of conventional wells, log of conventional output, and the entire county‐level age distribution.  U.S. Census Bureau, Small Area Income and Poverty Estimates (SAIPE). | Adjusted β Coefficient for county-level hospitalization rates for:  Pneumonia for age group 65+ yrs associated with county having an unconventional well last year (vs current year)1.509, *p<*0.01 (representing an increase of 1.5 admissions/1000 people); 0.995, *p<*0.01, *p<*0.05 after adjustment for county specific linear trends  (representing 1 admission additional admission/1000)  AMI:  All ages associated with 1^st^ lag of log of unconventional well’s output:  0.0.019, *p<*0.01 and 0.011, *p<*0.05 (adjusted for linear trends)  Age 20 – 44 years associated with county having an unconventional well last year (vs current year):  0.102, *p*<0.05, 0.113, *p* <0.05 after adjustment for county specific linear trends  Age 65+ associated with 1^st^ lag of log of unconventional well’s output::  0.070 , *p*<0.05, no effect when adjusted for county linear trend  Asthma for age grp 20-44 yrs:  0.099, *p<*0.05 and 0.146, *p<*0.01 (adjusted for county linear trends)  Significant association of county-level emissions of CO, NO_x_, PM_10_, PM_2.5_, SO_x_, VOC and number of new unconventional wells. | We find a significant association between shale gas development and hospitalizations for pneumonia among the elderly, which is consistent with higher levels of air pollution resulting from UNGD.  Significant association of well drilled in the year before hospitalization and hospitalization for AMI among those aged 45 and above, and for asthma and chronic obstructive pulmonary disease among those aged 20-44. | |
| 50 | Schuele et al., 2022 | Maternal-infant pairs in counties of 28 states with natural gas development  US (28 states)  33,849,409  (singleton births)  2005 - 2018 | TBW, LBW, SGA, PTB, gestational age, and interaction effect of racialized groups.  Birth records linked to exposure data (natural gas production) by country of birth.  Birth records data. | Linear regression models for TBW and gestational age, and probit models for the dichotomous outcomes of LBW, PTB, and SGA. Further examined interactions between women’s race/ethnicity and Natural gas development (NGD) production. | Race/ ethnicity, education, age, nativity, marital status, parity, prenatal care initiation, prenatal smoking, infant sex, and gestational age (except for outcomes of gestational age and PTB).  Birth records data. | Semi-elasticities (95% CI) of the impact of prenatal exposure to a 10% increase in NGD production activity (averaged over 9 months) on birth outcomes overall  TBW: -1.48 (−2.60, − + 0.37)  Gestational age: 0.01 (0.00, 0.02)  LBW: 0.0008 (0.0006, 0.0010)  SGA: 0.0018 (0.0015, 0.0022)  PTB: −0.0008 (−0.0012, −0.0005) | A 10% increase in NGD production in a county was associated with a decrease in mean birth weight by 1.48 g (95% CI = −2.60, −0.37), with reductions of 10.19 g (−13.56, −6.81) for infants born to Black women and 2.76 g (−5.05, −0.46) for infants born to Asian women. A 10% increase in NGD production in a county was associated with an increased risk of infants born LBW (0.0008; 95% CI = 0.0006, 0.0010) or small-for-gestational age (0.0018; 95% CI = 0.0015, 0.0022), particularly among infants born to Black women. | |
| 51 | Willis et al., 2018 | Children aged 2-18 yrs living in 29 counties in rural Pennsylvania located on the Marcellus shale  US (Pennsylvania)  15,837 hospital admissions  2003 - 2014 | Binary indicator of whether there is a pediatric asthma hospitalization in a specific zip code quarter-year, stratified into three age-specific categories (2-6, 7-12, and 13-18 yrs).  Pennsylvania Healthcare Cost Containment Council data (PHC4). | Zip code (571 zip codes) level analysis.  Fit mixed effects logistic regression models with a random intercept for zip code and fixed effects for year and quarter. | Sex, race, ethnicity, year, quarter, insurance type, zip code respiratory hazard index, county median household index, county unemployment, county poverty under 18 yrs old, and county log population density by zip code.  Exposure covariate for conventional oil and gas development to adjust for potential co-exposure effects; composite metric for non-UNGD respiratory hazards.  Pennsylvania Healthcare Cost Containment Council data (PHC4)  2010 Population Census  Pennsylvania Department of Environmental Protection.  2011 National Air Toxics Assessment (NATA) respiratory hazard index (RHI). | Tertile exposure metrics with no UOGD exposure as the ref:  OR (95% CI):  All ages:  Low exposure: 1.15 (0.97, 1.36)  Med exposure: 1.12 (0.94, 1.34)  High exposure: 1.12 (0.94, 1.34)  2-6 yrs:  Low exposure: 1.24 (1.00, 1.54)  Med exposure: 1.27 (1.01, 1.59)  High exposure: 1.73 (1.34, 2.23)  7-12 yrs:  Low exposure: 1.06 (0.84, 1.34)  Med exposure: 1.00 (0.79, 1.27)  High exposure: 1.11 (0.84, 1.47)  13-18 yrs:  Low exposure: 1.28 (1.06, 1.55)  Med exposure: 1.25 (1.03, 1.53)  High exposure: 1.35 (1.08, 1.70)  OR (95% CI) for newly spudded well (yes/no):  All ages: 1.25 (1.07, 1.47)  2-6 yrs: 1.44 (1.18, 1.75)  7-12 yrs: 1.03 (0.83, 1.29)  13-18 yrs: 1.34 (1.13, 1.60)  Ever-spudded well (yes/no):  All ages: 1.19 (1.04, 1.36)  2-6 yrs: 1.35 (1.14, 1.60)  7-12 yrs: 1.05 (0.88, 1.25)  13-18 yrs: 1.29 (1.11, 1.49)  Significant associations of log-sum for some emissions (e.g. 2,2,4-trimethylpentane, formaldehyde, x-hexane) and pediatric asthma hospitalizations. | We observed consistently elevated odds of hospitalizations in the top tertile of pediatric patients exposed to unconventional drilling compared with their unexposed peers.  During the same quarter a well was drilled, we find a 25% increase (95% CI: 1.07, 1.47) in the odds of being hospitalized for asthma.  Ever-establishment of an UNGD well within a zip code was associated with a 1.19 (95% CI: 1.04, 1.36) increased odds of a pediatric asthma hospitalization.  Our results further demonstrate that increasing specific air emissions from UNGD sites are associated with increased risks of pediatric asthma hospitalizations (e.g. 2,2,4-trimethylpentane, formaldehyde, x-hexane). These results hold across multiple age groups and sensitivity analyses.  Community-level UNGD exposure metrics were associated with increased odds of pediatric asthma-related hospitalization among young children and adolescents. | |
| 52 | Willis et al., 2020 | Children aged 1-17 yrs living in zip codes fully located on a shale play or basin  US (Texas)  54,956 observations:, 24,333 (unexposed) & 48,589 (exposed) hospital admissions  2000 – 2010  (11 yrs quarterly data) | Binary indicator of pediatric asthma hospitalization in a specific zip code quarter-year, stratified into three age-specific categories (1-4, 5-9, 10-14, 15-17 yrs).  Inpatient Data File from Texas Department of State Health Service. | Zip code (1249 zip codes) level analysis.  Logistic regression at the zip code level with the exposure metrics (any drilling, conventional, unconventional, flaring, production) and additional community characteristics (listed above) (Model 1); additional models with year and quarter fixed effects (Model 2) and year, quarter and county fixed effects (Model 3). | e Exposure covariates include cumulative number of unconventional sites  drilled in that observation; zip code level community characteristics: historical drilling activity in 1990–99, 2005 NATA total respiratory hazard, area of zip code in square kilometers, 2000 population density of people <18 yrs old by zip code, 2000 census percent Hispanic by zip code, 2000 census percent non White by zip code, annual county poverty percent <18 yrs old, annual county unemployment rate and annual county median household income; temporal (year and quarter)  2000 Census data, National Air Toxics Assessment data (NATA), small Area Income and Poverty Estimates data. | OR (95% CI), Model 3 compared to ref (no drilling):  All drilling:  1^st^ tertile: 1.22 (1.12, 1.32)  2^nd^ tertile: 1.51 (1.40, 1.64)  3^rd^ tertile: 1.27 (1.16, 1.39)  Conventional drilling:  1^st^ tertile: 1.20 (1.11, 1.31)  2^nd^ tertile: 1.23 (1.13, 1.34)  3^rd^ tertile: 1.10 (0.99, 1.22)  Unconventional drilling:  1^st^ tertile: 1.24 (1.13, 1.35)  2^nd^ tertile: 1.59 (1.46, 1.73)  3^rd^ tertile: 1.49 (1.36, 1.64)  Flaring volume:  1^st^ tertile: 1.25 (1.02, 1.54)  2^nd^ tertile: 1.13 (0.93, 1.38)  3^rd^ tertile: 0.68 (0.55, 0.84)  Production volume:  1^st^ tertile: 1.23 (1.13, 1.33)  2^nd^ tertile: 1.45 (1.34, 1.47)  3^rd^ tertile: 1.53 (1.38, 1.69) | We observed increased odds of 1 paediatric asthma hospitalization in a zip code per quarter associated with increasing tertiles of natural gas development NGD exposure and show that spatiotemporal variation impacts results.  Increasing production volumes are associated with increased paediatric asthma hospitalizations in an exposure–response relationship, whereas associations with flaring volumes are inconsistent  We found evidence of associations between paediatric asthma hospitalizations and NGD, regardless of drilling type. Practices related to production volume may be driving these positive associations | |

Abbreviations: AAVD = aortic artery and valve defect; AEC = annual emissions concentration; ALE = annualized loss expectancy; ALL = acute lymphocytic leukemia; AMI = acute myocardial infarction; BMI = body mass index; CCHD = critical congenital heart defects; CHD = congenital heart defects; CI = confidence interval; CO = carbon monoxide; CWS = community water source; DNDW = distance to the nearest drilled well; ED = emergency department; FEV1 = forced expiratory volume during first second; FVC = forced vital capacity; g = grams; HFpEF = heart failure preserved ejection fraction; HFrEF = heart failure reduced ejection fraction; IA-IDW = intensity-adjusted inverse distance weighted measure; IDW = inverse distance weighted metric; ID^2^W = inverse distance weighted squared metric; IL = interleukin; km = kilometres; LBW = Low birthweight; m = metres; Med = medium; mm Hg = millimetres of Mercury; MPA = Migraine Probability Algorithm; NGD = natural gas development; NO_x_ = nitrogen oxides; NTD = neural tube defects; O&G = oil and gas; OGD = oil and gas development; OR = Odds ratio; PADEP = Pennsylvania Department of Environmental Protection; PM = particulate matter; ppb = parts per billion; PTB = Preterm birth; Ref = reference; RR = Risk ratio; Rx = prescription; SD = standard deviation; SGA = Small for gestational age; SIR = Standardized incidence ratio; SO_x_ = sulphur oxides; TBW = Term birthweight; TNF = tumour necrosis factor; µg = microgram; UGD = unconventional gas development; UNGD = Unconventional natural gas development; UOG = unconventional oil and gas; UOGD = unconventional oil and gas development; US = United States; VOCs = volatile organic compounds; vs. = versus; w/ = with; WIC = Special Supplemental Food Program for Women, Infants and Children; yrs = years
Main effect estimates are those reported by the authors in the abstract and do not include sensitivity analyses or subgroup analyses not reported in the abstract.

# **Online Resource 4:** Further synthesis of health outcomes

## Other infant outcomes

One cohort (McKenzie et al., 2014) and one case-control study (Tang et al., 2021) found increased odds of neural tube, but not oral cleft deformities, associated with maternal proximity to UOGD activity (Table 2). Tang et al. reported relatively large effect magnitudes (OR 2.94, 95% CI 1.83-4.75 and 2.09, 95% CI 1.47-2.99) for anencephaly and spina bifida, respectively (Tang et al., 2021). The associations were stronger among Hispanic women verses non-Hispanic women. Two of the studies reported significantly positive linear tests for trend consistent with a linear dose-response relationship (McKenzie et al., 2014; Tang et al., 2021). Cairncross et al. (Cairncross et al., 2022) reported higher risk of any major congenital anomalies (RR 1.31, 95% CI 1.01-1.69) among infants born to mothers living in rural Alberta within 10 km of at least one HF well during preconception and pregnancy.

An ecologic study in Pennsylvania reported no effect of UOGD on a combined measure of all congenital birth defects (Ma et al., 2016). The study also found that the prevalence of birth defects post-drilling was lower than the prevalence before any drilling occurred in areas with UOGD. However, a retrospective cohort study of over two million births in Texas implemented a difference in difference analysis, and reported an increased risk of all congenital abnormalities in the highest exposure tertile across various exposure metrics (by site count, or monthly production of oil, gas, and water) (Willis et al., 2023).

Additionally, two studies reported associations of UOGD with lower composite infant health index (Apergis et al., 2019; Currie et al., 2017). Others found evidence of an association of UOGD activity exposure with fetal death (Whitworth et al., 2017) and infant mortality (Busby and Mangano, 2017).

## Cancer outcomes

Two case-control studies examined acute childhood lymphocytic leukemia (Clark et al., 2022; McKenzie et al., 2017) (Table 2). In Colorado, McKenzie et al. found children with acute lymphocytic leukemia were 4.3 times more likely to live in the highest UOGD activity tertile compared to controls (OR 4.3, 95% CI 1.1-16), with significantly increasing magnitudes of effect estimates across tertiles. In Pennsylvania, associations between UOGD exposure were only significant within a 2 km radius in children aged 2-7 years and when considering a perinatal exposure window (OR 2.80; 95 % CI 1.11-7.05), but not within 5 or 10 km, or when the exposure window was set to childhood exposure (Clark et al., 2022). The study suggests that exposure during preconception to birth is an important etiological window.

Several ecologic studies have also reported an association of UOGD and higher inpatient rates of oncology (Jemielita et al., 2015), adult bladder cancer (Finkel, 2016) and childhood central nervous system tumours (Fryzek et al., 2013), but were all subject to bias due to weaker study designs.

## Cardiovascular outcomes

A case-control study with 12,330 participants in Pennsylvania (McAlexander et al., 2020) reported a significant association with heart failure hospitalizations. A strength of the study was its incorporation of different exposure metrics that accounted for pad preparation, spuds, stimulation, and drilling phases. Although linear increases of risk were identified for all phases (except spuds), the association between UOGD exposure and heart failure was strongest during the stimulation phase (Q4 versus Q1 OR 1.80; 95% CI 1.35-2.40). One Colorado cross-sectional study (McKenzie et al., 2019b) reported an association with laboratory and clinical cardiovascular risk markers and a few ecological studies reported significant association of UOGD activity exposure with a variety of adverse cardiovascular outcomes (Apergis et al., 2021; Denham et al., 2021; Hu et al., 2022; Jemielita et al., 2015; Peng et al., 2018) (Table 1).

## Self reported symptoms

Cross-sectional survey studies from Ohio, Colorado, and Pennsylvania examined self-reported health symptoms and residential UOGD proximity (Blinn et al., 2020; Elliott et al., 2018; Mayer et al., 2021; Rabinowitz et al., 2015; Steinzor et al., 2013; Tustin et al., 2017). UOGD was associated with significantly higher rates of upper- and/or lower-respiratory tract symptoms (Blinn et al., 2020; Rabinowitz et al., 2015; Steinzor et al., 2013), skin irritation (Rabinowitz et al., 2015; Steinzor et al., 2013), fatigue (Blinn et al., 2020; Elliott et al., 2018), and headache (Blinn et al., 2020; Steinzor et al., 2013). A case-control study in California examined the association between the IDW for both methane gas super-emitters and active oil and gas wells within 10 km of residence and average block-group level methane, NO_2_, and PM_2.5_ and migraine diagnosis (Elser et al., 2021). NO_2_ and IDW methane emission measures were associated with increased odds of case status. No effect was observed with oil and gas wells exposure.

One study conducted direct environmental testing of air and water at 70 respondents’ homes for VOCs in air, and VOCs, alpha/beta radiation, radon, and radium in water. Respondents reported 50–73% concordance of symptoms with detection of these chemicals (Steinzor et al., 2013). UOGD proximity was also associated with significantly lower self-reported satisfaction with health after adjusting for a range of potential confounders including trust in regulators and a belief that UOGD activity caused stress (Mayer et al., 2021).

## Mortality

Evidence suggests increased mortality rates among populations living proximal to various UOGD exposure measures (Apergis et al., 2019; Denham et al., 2021; Hu et al., 2022; Li et al., 2022). One retrospective cohort study of 136 million seniors living in regions with UOGD activity from 2001 – 2015, reported the association was stronger when combining the IDW with downwind metrics (High IDW, upwind Hazard Ratio (HR) 1.022 (95% CI 1.017-.028); High IDW, downwind HR 1.031 (95% CI 1.025-1.037)) (Li et al., 2022). In Oklahoma, an ecological study detected a 4.2% reduction in life expectancy associated with a 1% increase in the number of UOGD wells in a county, with life expectancy reductions driven mostly by increases in cancer and cardiac disease (Apergis et al., 2021). Cerebrovascular mortality was also associated with UOGD exposure in another ecological study across 49 states in the US (Hu et al., 2022).

# **References:**

Apergis, N., Hayat, T., Saeed, T., 2019. Fracking and infant mortality: fresh evidence from Oklahoma. Environmental science and pollution research international 26, 32360–7.

Apergis, N., Mustafa, G., Dastidar, S.G., 2021. An analysis of the impact of unconventional oil and gas activities on public health: New evidence across Oklahoma counties. Energy Economics 97, 105223. https://doi.org/10.1016/j.eneco.2021.105223

Blinn, H.N., Utz, R.M., Greiner, L.H., Brown, D.R., 2020. Exposure assessment of adults living near unconventional oil and natural gas development and reported health symptoms in southwest Pennsylvania, USA. PLoS One 15.

Brown, D.R., Greiner, L.H., Weinberger, B.I., Walleigh, L., Glaser, D., 2019. Assessing exposure to unconventional natural gas development: using an air pollution dispersal screening model to predict new-onset respiratory symptoms. J Environ Sci Health A Tox Hazard Subst Environ Eng 54, 1357–1363. https://doi.org/10.1080/10934529.2019.1657763

Busby, C., Mangano, J.J., 2017. There’s a World Going on Underground—Infant Mortality and Fracking in Pennsylvania. Journal of Environmental Protection 8, 381–393. https://doi.org/10.4236/jep.2017.84028

Bushong, A., McKeon, T., Regina Boland, M., Field, J., 2022. Publicly available data reveals association between asthma hospitalizations and unconventional natural gas development in Pennsylvania. PLoS One 17, e0265513. https://doi.org/10.1371/journal.pone.0265513

Cairncross, Z.F., Couloigner, I., Ryan, M.C., McMorris, C., Muehlenbachs, L., Nikolaou, N., 2022. Association Between Residential Proximity to Hydraulic Fracturing Sites and Adverse Birth Outcomes. JAMA Pediatrics Jun;176(6):585-92.

Caron-Beaudoin, Whitworth, K.W., Bosson-Rieutort, D., Wendling, G., Liu, S., Verner, M.-A., 2021. Density and proximity to hydraulic fracturing wells and birth outcomes in Northeastern British Columbia, Canada. J Expo Sci Environ Epidemiol 31, 53–61. https://doi.org/10.1038/s41370-020-0245-z

Casey, J.A., Savitz, D.A., Rasmussen, S.G., Ogburn, E.L., Pollak, J., Mercer, D.G., 2016. Unconventional Natural Gas Development and Birth Outcomes in Pennsylvania, USA. Epidemiology 27, 163–72.

Clark, C.J., Johnson, N.P., Soriano, M., Warren, J.L., Sorrentino, K.M., Kadan-Lottick, N.S., Saiers, J.E., Ma, X., Deziel, N.C., 2022. Unconventional Oil and Gas Development Exposure and Risk of Childhood Acute Lymphoblastic Leukemia: A Case-Control Study in Pennsylvania, 2009-2017. Environ Health Perspect 130, 87001. https://doi.org/10.1289/EHP11092

Currie, J., Greenstone, M., Meckel, K., 2017. Hydraulic fracturing and infant health: New evidence from Pennsylvania. Science advances 3.

Cushing, L.J., Vavra-Musser, K., Chau, K., Franklin, M., Johnston, J.E., 2020. Flaring from Unconventional Oil and Gas Development and Birth Outcomes in the Eagle Ford Shale in South Texas. Environ Health Perspect 128. https://doi.org/10.1289/EHP6394

Denham, A., Willis, M., Zavez, A., Hill, E., 2019. Unconventional natural gas development and hospitalizations: evidence from Pennsylvania, United States, 2003–2014. Public Health 168, 17–25.

Denham, A., Willis, M.D., Croft, D.P., Liu, L., Hill, E.L., 2021. Acute myocardial infarction associated with unconventional natural gas development: A natural experiment. Environ Res 195.

Elliott, E.G., Ma, X., Leaderer, B.P., McKay, L.A., Pedersen, C.J., Wang, C., 2018. A community-based evaluation of proximity to unconventional oil and gas wells, drinking water contaminants, and health symptoms in Ohio. Environmental Research 167, 550–7.

Elser, H., Morello-Frosch, R., Jacobson, A., Pressman, A., Kioumourtzoglou, M.-A., Reimer, R., Casey, J.A., 2021. Air pollution, methane super-emitters, and oil and gas wells in Northern California: the relationship with migraine headache prevalence and exacerbation. Environmental Health 20, 45. https://doi.org/10.1186/s12940-021-00727-w

Erickson, C.L., Barron, I.G., Zapata, I., 2022. The effects of hydraulic fracturing activities on birth outcomes are evident in a non-individualized county-wide aggregate data sample from Colorado. Journal of Public Health Research 11, jphr.2021.2551. https://doi.org/10.4081/jphr.2021.2551

Finkel, M.L., 2016. Shale gas development and cancer incidence in southwest Pennsylvania. Public Health 141, 198–206.

Fryzek, J., Pastula, S., Jiang, X., Garabrant, D.H., 2013. Childhood cancer incidence in Pennsylvania counties in relation to living in counties with hydraulic fracturing sites. Journal of occupational and environmental medicine 55, 796–801.

Hill, E.L., 2018. Shale gas development and infant health: Evidence from Pennsylvania. Journal of Health Economics 61, 134–50.

Hill, E.L., Ma, L., 2022. Drinking water, fracking, and infant health. Journal of Health Economics 82, 102595. https://doi.org/10.1016/j.jhealeco.2022.102595

Hu, C., Liu, B., Wang, S., Zhu, Z., Adcock, A., Simpkins, J., Li, X., 2022. Spatiotemporal Correlation Analysis of Hydraulic Fracturing and Stroke in the United States. Int J Environ Res Public Health 19, 10817. https://doi.org/10.3390/ijerph191710817

Janitz, A.E., Dao, H.D., Campbell, J.E., Stoner, J.A., Peck, J.D., 2019. The association between natural gas well activity and specific congenital anomalies in Oklahoma, 1997-2009. Environ Int 122, 381–8.

Jemielita, T., Gerton, G.L., Neidell, M., Chillrud, S., Yan, B., Stute, M., 2015. Unconventional Gas and Oil Drilling Is Associated with Increased Hospital Utilization Rates. PLoS One 10.

Johnston, J.E., Enebish, T., Eckel, S.P., Navarro, S., Shamasunder, B., 2021. Respiratory health, pulmonary function and local engagement in urban communities near oil development. Environ Res 197, 111088. https://doi.org/10.1016/j.envres.2021.111088

Koehler, K., Ellis, J.H., Casey, J.A., Manthos, D., Bandeen-Roche, K., Platt, R., 2018. Exposure Assessment Using Secondary Data Sources in Unconventional Natural Gas Development and Health Studies. Environmental Science & Technology 52, 6061–9.

Li, L., Dominici, F., Blomberg, A.J., Bargagli-Stoffi, F.J., Schwartz, J.D., Coull, B.A., Spengler, J.D., Wei, Y., Lawrence, J., Koutrakis, P., 2022. Exposure to unconventional oil and gas development and all-cause mortality in Medicare beneficiaries. Nat Energy 1–9. https://doi.org/10.1038/s41560-021-00970-y

Ma, Z.Q., L, L., LH, K., 2016. Time Series Evaluation of Birth Defects in Areas with and without Unconventional Natural Gas Development. Journal of Epidemiology and Public Health Reviews 1.

Makati, D., Akers, J., Aljuhani, M., Pellegrino, B., Schmidt, R., Shawwa, K., Kannabhiran, D., 2022. Prevalence of ANCA-associated vasculitis amid natural gas drilling sites in West Virginia. J Nephrol 35, 1185–1192. https://doi.org/10.1007/s40620-021-01243-3

Mayer, A., Malin, S., McKenzie, L., Peel, J., Adgate, J., 2021. Understanding Self-Rated Health and Unconventional Oil and Gas Development in Three Colorado Communities. Society & Natural Resources 34, 60–81.

McAlexander, T.P., Bandeen-Roche, K., Buckley, J.P., Pollak, J., Michos, E.D., McEvoy, J.W., 2020. Unconventional Natural Gas Development and Hospitalization for Heart Failure in Pennsylvania. Journal of the American College of Cardiology 76, 2862–74.

McKenzie, L.M., Allshouse, W.B., Byers, T.E., Bedrick, E.J., Serdar, B., Adgate, J.L., 2017. Childhood hematologic cancer and residential proximity to oil and gas development. PLoS One 12.

McKenzie, L. M., Allshouse, W., & Daniels, S. (2019a). Congenital heart defects and intensity of oil and gas well site activities in early pregnancy. *Environ Int, 132*, 104949. doi:10.1016/j.envint.2019.104949

McKenzie, L.M., Crooks, J., Peel, J.L., Blair, B.D., Brindley, S., Allshouse, W.B., 2019b. Relationships between indicators of cardiovascular disease and intensity of oil and natural gas activity in Northeastern Colorado. Environmental Research 170, 56–64.

McKenzie, L.M., Guo, R., Witter, R.Z., Savitz, D.A., Newman, L.S., Adgate, J.L., 2014. Birth outcomes and maternal residential proximity to natural gas development in rural Colorado. Environ Health Perspect 122, 412–7.

Newcomb, P., Li, J., 2019. Predicting admissions for adult asthma exacerbations in North Texas. Public Health Nurs 36, 779–786. https://doi.org/10.1111/phn.12654

Peng, L., Meyerhoefer, C., Chou, S.Y., 2018. The health implications of unconventional natural gas development in Pennsylvania. Health Econ 27, 956–83.

Rabinowitz, P.M., Slizovskiy, I.B., Lamers, V., Trufan, S.J., Holford, T.R., Dziura, J.D., 2015. Proximity to natural gas wells and reported health status: results of a household survey in Washington County, Pennsylvania. Environ Health Perspect 123, 21–6.

Rasmussen, S.G., Ogburn, E.L., McCormack, M., Casey, J.A., Bandeen-Roche, K., Mercer, D.G., 2016. Association Between Unconventional Natural Gas Development in the Marcellus Shale and Asthma Exacerbations. JAMA Intern Med.

Schuele, H., Baum, C.F., Landrigan, P.J., Hawkins, S.S., 2022. Associations between proximity to gas production activity in counties and birth outcomes across the US. Prev Med Rep 30, 102007. https://doi.org/10.1016/j.pmedr.2022.102007

Stacy, S.L., Brink, L.L., Larkin, J.C., Sadovsky, Y., Goldstein, B.D., Pitt, B.R., 2015. Perinatal outcomes and unconventional natural gas operations in Southwest Pennsylvania. PLoS One 10.

Steinzor, N., Subra, W., Sumi, L., 2013. Investigating links between shale gas development and health impacts through a community survey project in Pennsylvania. New Solut 23, 55–83.

Tang, I.W., Langlois, P.H., Vieira, V.M., 2021. Birth defects and unconventional natural gas developments in Texas, 1999-2011. Environ Res 194, 110511. https://doi.org/10.1016/j.envres.2020.110511

Tran, K.V., Casey, J.A., Cushing, L.J., Morello-Frosch, R., 2021. Residential proximity to hydraulically fractured oil and gas wells and adverse birth outcomes in urban and rural communities in California (2006–2015). Environ Epidemiol 5, e172. https://doi.org/10.1097/EE9.0000000000000172

Tran, K.V., Casey, J.A., Cushing, L.J., Morello-Frosch, R., 2020. Residential proximity to oil and gas development and birth outcomes in California: A retrospective cohort study of 2006-2015 births. Environ Health Perspect Jun;128(6):67001.

Tustin, A.W., Hirsch, A.G., Rasmussen, S.G., Casey, J.A., Bandeen-Roche, K., Schwartz, B.S., 2017. Associations between Unconventional Natural Gas Development and Nasal and Sinus, Migraine Headache, and Fatigue Symptoms in Pennsylvania. Environ Health Perspect Feb;125(2):189-97.

Walker Whitworth, K., Symanski, E, 2018. Drilling and Production Activity Related to Unconventional Gas Development and Severity of Preterm Birth. Environmental health perspectives 126. https://doi.org/doi.org/10.1289/EHP2622

Whitworth, K.W., Marshall, A.K., Symanski, E., 2017. Maternal residential proximity to unconventional gas development and perinatal outcomes among a diverse urban population in Texas. PloS One 12.

Willis, M., Hill, E.L., Kile, M.L., Carozza, S., Hystad, P., 2022. Associations between residential proximity to oil and gas extraction and hypertensive conditions during pregnancy: a difference-in-differences analysis in Texas, 1996-2009. Int J Epidemiol 51, 525–536. https://doi.org/10.1093/ije/dyab246

Willis, M., Hystad, P., Denham, A., Hill, E., 2020. Natural gas development, flaring practices and paediatric asthma hospitalizations in Texas. International Journal of Epidemiology 49, 1883–1896. https://doi.org/10.1093/ije/dyaa115

Willis, M.D., Carozza, S.E., Hystad, P., 2023. Congenital anomalies associated with oil and gas development and resource extraction: a population-based retrospective cohort study in Texas. J Expo Sci Environ Epidemiol 33, 84–93. https://doi.org/10.1038/s41370-022-00505-x

Willis, M.D., Hill, E.L., Boslett, A., Kile, M.L., Carozza, S.E., Hystad, P., 2021. Associations between Residential Proximity to Oil and Gas Drilling and Term Birth Weight and Small-for-Gestational-Age Infants in Texas: A Difference-in-Differences Analysis. Environ Health Perspect 129, 077002. https://doi.org/10.1289/EHP7678

Willis, M.D., Jusko, T.A., Halterman, J.S., Hill, E.L., 2018. Unconventional natural gas development and pediatric asthma hospitalizations in Pennsylvania. Environmental Research 166, 402–8.
